# Molecular mechanisms of bifunctional vitamin D receptor agonist-histone deacetylase inhibitor hybrid molecules in triple-negative breast cancer

**DOI:** 10.1038/s41598-022-10740-9

**Published:** 2022-04-25

**Authors:** Camille Barbier, Ali Mansour, Aiten Ismailova, Fatemeh Sarmadi, David A. Scarlata, Manuella Bouttier, Camille Zeitouni, Catherine Wang, James L. Gleason, John H. White

**Affiliations:** 1 Departments of Physiology, McGill University, Montreal, QC Canada; 2 Departments of Chemistry, McGill University, Montreal, QC Canada; 3 Departments of Medicine, McGill University, Montreal, QC Canada

**Keywords:** Cancer, Chemical biology, Computational biology and bioinformatics, Chemistry

## Abstract

The active form of vitamin D, 1,25-dihydroxyvitamin D (1,25D), and its analogues signal through the nuclear vitamin D receptor (VDR), a ligand-regulated transcription factor, and have been extensively investigated as anticancer agents. 1,25D and its analogs have potential in combination therapies because they exhibit synergistic activities with other anticancer agents such as histone deacetylase inhibitors (HDACi). We have developed a series of hybrid molecules that combine HDACi within the backbone of a VDR agonist and thus represent fully integrated bifunctional molecules. They exhibit anti-tumor efficacy in reducing tumor growth and metastases in an aggressive model of triple-negative breast cancer. However, their solubility is limited by their hydrophobic diarylpentane cores. Our goals here were two-fold: (1) to improve the solubility of hybrids by introducing nitrogen into diarylpentane cores, and (2) to investigate the molecular mechanisms underlying their anti-tumor efficacy by performing comparative gene expression profiling studies with 1,25D and the potent HDACi suberoylanilide hydroxamic acid (SAHA). We found that substituting aryl with pyrydyl rings did not sacrifice bifunctionality and modestly improved solubility. Notably, one compound, AM-193, displayed enhanced potency as a VDR agonist and in cellular assays of cytotoxicity. RNAseq studies in triple negative breast cancer cells revealed that gene expression profiles of hybrids were very similar to that of 1,25D, as was that observed with 1,25D and SAHA combined. The effects of SAHA alone on gene expression were limited and distinct from those 1,25D or hybrids. The combined results suggest that efficacy of hybrids arises from targeting HDACs that do not have a direct role in gene regulation. Moreover, pathways analysis revealed that hybrids regulate numerous genes controlling immune cell infiltration into tumors and suppress the expression of several secreted molecules that promote breast cancer growth and metastasis.

## Introduction

**Figure 1:**
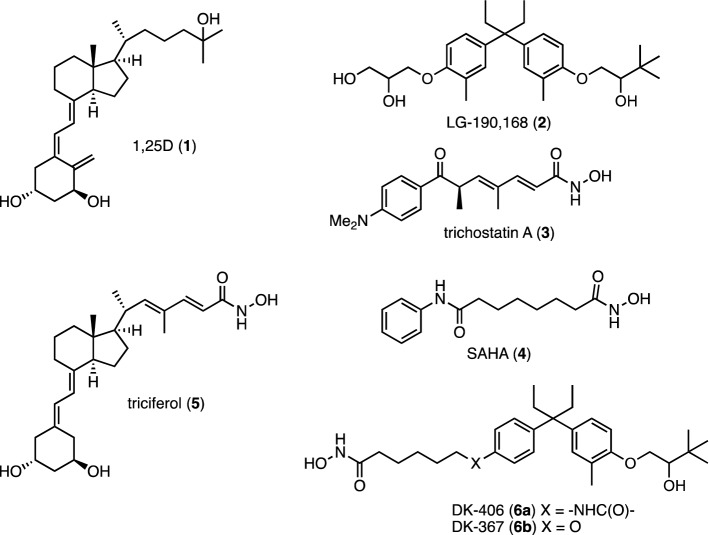
Structures of VDR agonists, HDAC inhibitors and VDR agonist/HDACi hybrids.

Vitamin D is a secosteroid obtained naturally from limited dietary sources and from exposure of skin to sufficient solar ultraviolet B (UVB) irradiation via the photochemical and thermal conversion of 7-dehydrocholesterol. The active form of vitamin D, 1,25-dihydroxyvitamin D (1,25D, **1**, Fig. 1) is produced via sequential hydroxylations and signals through the vitamin D receptor (VDR), a ligand-regulated transcription factor^1,2^. 1,25D has attracted extensive interest because of its “non-classical actions”, including its potential as an anticancer agent^1^. This has spurred the development of numerous secosteroidal and non-secosteroidal analogues of 1,25D, and mechanistic studies have been greatly facilitated by the fact that the VDR is a soluble, well-defined and experimentally tractable target^3^. Several preclinical studies in multiple cancer models have provided evidence for the anti-tumor activities of 1,25D or its analogues^4–6^. In spite of these successes, 1,25D and its analogues have failed as monotherapies in cancer because of acquired tumor resistance, even though vitamin D signaling often remains intact in resistant cells. In addition, results of cancer prevention trials with vitamin D are mixed, although these are notoriously difficult to conduct and interpret because vitamin D is a nutrient. In addition to supplementation, it is obtained in the diet and by cutaneous UVB exposure, and thus there is effectively no placebo wing in such trials. Nonetheless, a meta-analysis of intervention trials suggested that vitamin D supplementation reduced total cancer mortality^7^.

Although they do not respond to 1,25D alone, resistant cancer cells are sensitive to the combination of 1,25D and histone deacetylase inhibitors (HDACi)^8,9^, suggesting that vitamin D analogues may have a role in combination therapies. HDACi are in active development as cancer therapeutics, including as components of combination therapies^10,11^. There are multiple classes of HDACs, and those that are zinc metalloenzymes are mostly class I and class II, along with HDAC11, which is class IV^12^. Although HDACs were initially characterized for their capacity to deacetylate histones, depending on the class, HDACs may be wholly or partially nuclear, or entirely cytoplasmic, and thus have numerous potential biochemical targets.

The synergism between 1,25D and HDACi led us to develop a series of fully integrated bifunctional hybrids that incorporate HDACi into the backbone of VDR agonists^13–16^. Initially, we generated the secosteroidal hybrid triciferol (**5**)^13^, based on the structures of 1,25D and the HDACi trichostatin A (**3**), followed by several non-secosteroidal hybrids based on the easily assembled diarylpentane core of LG-190,178 (**2**)^14,15^. Initially, the 25-hydroxy sidechain of 1,25D was adapted to incorporate a zinc-chelating hydroxamic acid necessary for HDACi activity (e.g. triciferol, **3**)^13^. Substitution of the 25-hydroxy surrogate of nonsecosteroidal analogues failed to produce bifunctional molecules, whereas, remarkably, incorporation of a hydroxamic acid into the 1-hydroxy surrogate, as in DK-406 (**6a**), DK-367 (**6b**) and other compounds, was successful^14,15^. We have found that there exists considerable synthetic latitude in optimizing HDACi activity within non-secosteroidal backbones, notably by modifying hydroxamic acid sidechain lengths, without substantially disrupting VDR agonism^15^.

In an in vitro screen of the 60 human cell line panel of the National Cancer Institute (US), DK-367 and related hybrid DK-366 displayed antiproliferative activities against cell lines derived from a broad range of tumors, including triple-negative breast cancers (TNBCs)^15^. We are interested in further developing hybrids as potential therapeutics for TNBC, which represent ~ 15% of all breast tumors^17,18^. TNBC tumors are so defined because they lack expression of the estrogen or progesterone receptors (ER/PR) found in approximately two-thirds of breast tumors or the cell surface receptor HER2 and are in need of efficacious targeted therapeutics. Notably, HDACs are expressed in TNBC cells and there is interest in the potential of HDACi in combination therapies for TNBC^19^. In previous work we found that, DK-367 and DK-406 reduced tumor growth and metastasis in the aggressive 4T1 mouse model of triple negative breast cancer (TNBC) under conditions where 1,25D and the HDACi SAHA (suberoylanilide hydoxamic acid; Vorinostat, **4**) were inactive alone or in combination^20^, thus providing strong validation for the hybrid concept.

While our initial analyses of efficacy of DK-406 and DK-367 were encouraging, their diarylpentane core limits their aqueous solubility. Thus, we explored the introduction of a nitrogen into the diarylpentane core and examined its effect on solubility and bifunctionality of the hybrids. Furthermore, the mechanism(s) of synergy between 1,25D and HDAC remain unclear. As both VDR signaling and HDACi can modulate gene transcription, we used RNAseq, to compare the transcriptional regulatory profiles of hybrids with those of 1,25D, SAHA, and 1,25D and SAHA together. We found that potent bifunctional hybrids can be produced by nitrogen substitutions into the diarylpentane core, with an accompanying improvement in solubility. We also found that expression profiles in 1,25D- or hybrid-treated cells are remarkably similar, consistent with gene expression is driven largely by VDR agonism, which suggests that HDACi activity of hybrids may target enzyme(s) not implicated in gene regulation. Moreover, hybrids inhibit the expression of genes encoding multiple classes of secreted proteins, including several chemokines implicated in promoting breast tumor growth and metastasis.

## Results and discussion

### Design and synthesis of heterocyclic VDR agonist/HDACi hybrids

**Figure 2:**
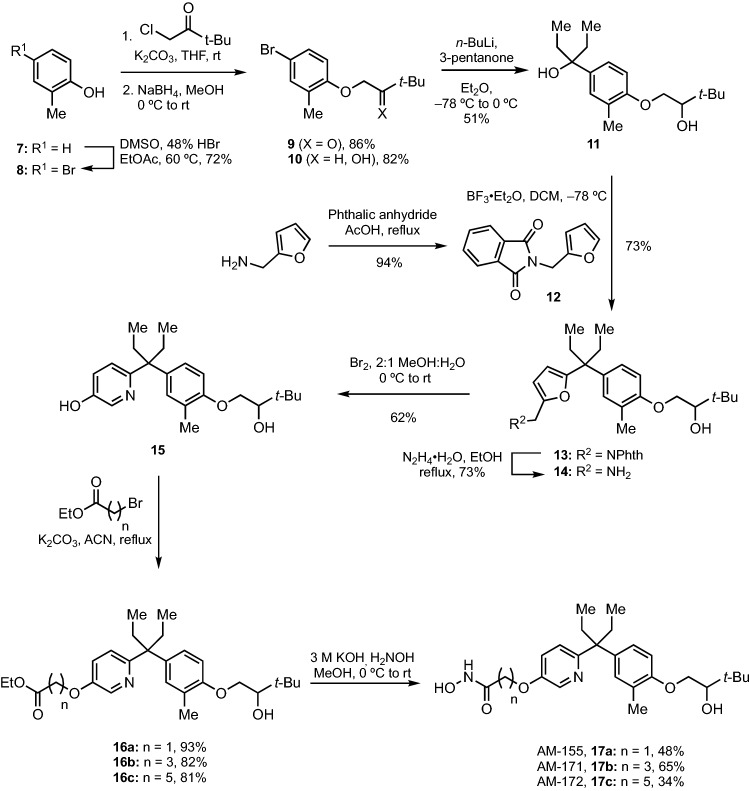
Synthesis of A-ring pyridine hybrids.

**Figure 3:**
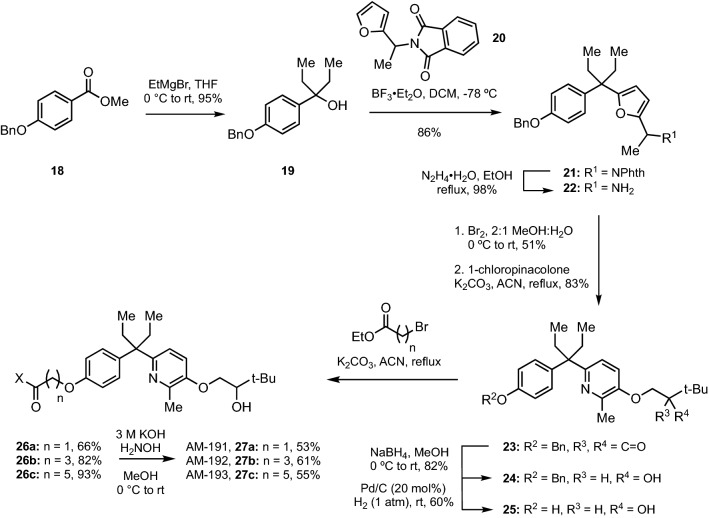
Synthesis of B-ring pyridine hybrids.

Binding of 1,25D or its analogues to the VDR is strongly dependent on interactions of the terminal 1-, 3- and 25-hydroxyl groups or their surrogates to key residues in the receptor ligand binding pocket^21^. The compatibility of the hydroxamic acid as a hydroxyl replacement in 1,25D and its analogs provided a route to introduction of HDAC inhibition into the structures of VDR agonists. This led to the development of the bifunctional analogue DK-406^15,20^. However, due to its hydrophobic 3,3-diarylpentane core, DK-406 possesses limited water solubility, a feature not uncommon among many commercial drugs, including SAHA^22^. A solution of 12.5% ethanol, 37.5% PEG-400 and 50% saline was used to solubilize DK-406 and related hybrids for previous in vivo studies^20^, but it would be desirable to produce compounds with improved water solubility without sacrificing bifunctionality. As a first step, we designed new heterocyclic analogues of DK-406, namely AM-155, -171 and -172 (**17a–c**, Fig. 2), and AM-191–193 (**26a–c**, Fig. 3), where either aromatic ring was replaced with pyridine derivatives, which would be expected to improve solubility in water while not significantly disrupting VDR agonism or HDACi activity. The computed partition coefficient (logP) of heterocyclic hybrids improved from ~ 6.5 for hybrids such as DK-367 to ~ 5.0 with the nitrogen substitution ortho to the 3,3-pentane linker being the more favourable (Fig. S1) bringing them into the normal logP range for drug-like molecules^23^.

The pyridine hybrids were synthesized by two similar routes based on a key aza-Achmatowicz rearrangement to access the core 3-pyridyl-3-arylpentane from a furfurylamine precursor. The synthesis of the A-ring hybrid series began with the mild *para*-bromination of *o*-cresol (**7**) using a system of DMSO and 48% aqueous HBr to afford 4-bromophenol **8** in 72% yield^24^. 4-Bromophenol **8** was subjected to *O*-alkylation with 1-chloropinacolone to form an α-alkoxyketone (**9**) which was then reduced to the corresponding secondary alcohol **10** with NaBH_4_ in 71% over two steps. Metal halogen exchange of **10** with *n*-BuLi followed by addition to 3-pentanone afforded tertiary alcohol **11** in 51% yield. Next, Friedel–Crafts alkylation with furfuryl phthalimide **12** in the presence of BF_3_•OEt_2_ afforded 3-furyl-3-arylpentane **13** in 74% yield. Deprotection using hydrazine liberated free amine **14** in 73% yield. With **14**, the precursor for the aza-Achmatowicz rearrangement in hand we examined the use of *m*-CPBA in dichloromethane, conditions first reported by Lefebvre^25,26^. While these produced the desired product **15**, the yield was disappointing (27%) due to over oxidation leading to oxime and pyridine *N*-oxide by-products. Fortunately, classical conditions for the regular Achmatowicz rearrangement^27^, using bromine in 1:2 H_2_O/MeOH, provided **15** in an acceptable 62% yield. Finally, *O*-alkylation of 3-hydroxypyridine **15** with C2, C4, and C6 alkyl bromide esters followed by treatment with hydroxylamine and KOH afforded A-ring hybrids AM-155 (n = 1, **17a**), AM-171 (n = 3, **17b**), and AM-172 (n = 5, **17c**) in 25–53% yields over two steps.

The synthesis of the B-ring series mirrored that of the A-ring series producing B-ring hybrids AM-191 (n = 1, **27a**), AM-192 (n = 3, **27b**), and AM-193 (n = 5, **27c**) in 9 steps (Fig. 3). As will be seen below, hybrid AM-193 proved to be the most promising of all the hybrids and thus we examined its aqueous solubility. Compared to its benzenoid analog DK-367 (**6b**), AM-193 possessed 27% higher solubility in water (2.43 µg/mL vs. 1.91 µg/mL) after sonication for 30 min.

### Biochemical characterization of heterocyclic VDR agonist/HDACi hybrids

**Figure 4:**
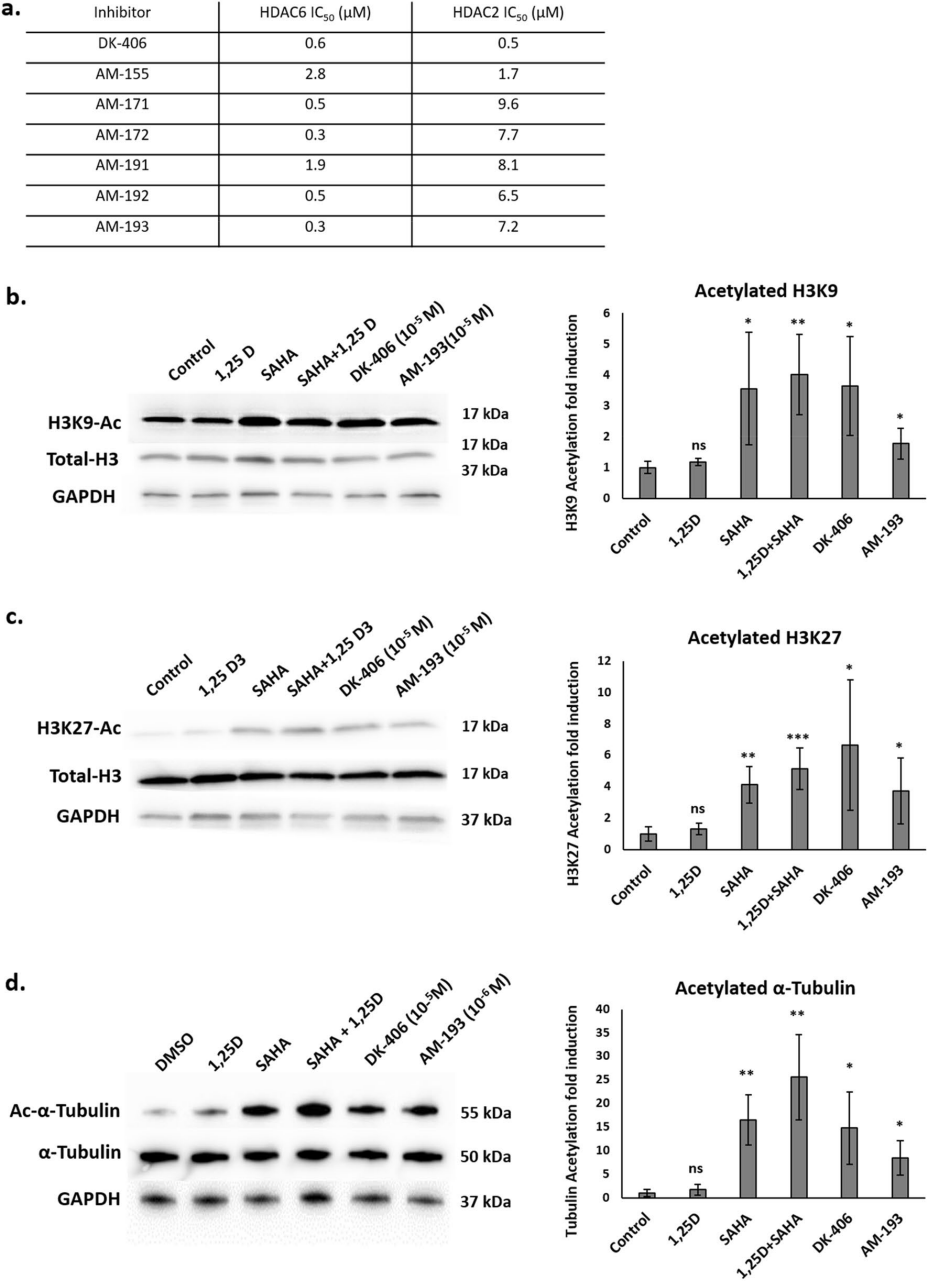
(**a**) Results of in vitro assays for inhibition of purified HDAC2 and HDAC6 by DK-406 and heterocyclic hybrids as measured by inhibition of deacetylation of a fluorometric substrate (see Methods for details). (**b–d**) Left: Western blotting analysis of histone 3 lysine 9 (H3K9; **b**), H3K27 (**c**), or acetylated tubulin (**d**), levels in 4TO7 cells treated with ligands for 6 h. Representative western-blots are shown from at least 3 biological replicates. Right: Graphs representing the average of the replicates, bars are mean ± SD. Each condition was compared to control. *: *p* ≤ 0.05, **: *p* ≤ 0.01, ***: *p* ≤ 0.001, ****: *p* ≤ 0.0001, ns: not significant.

**Figure 5:**
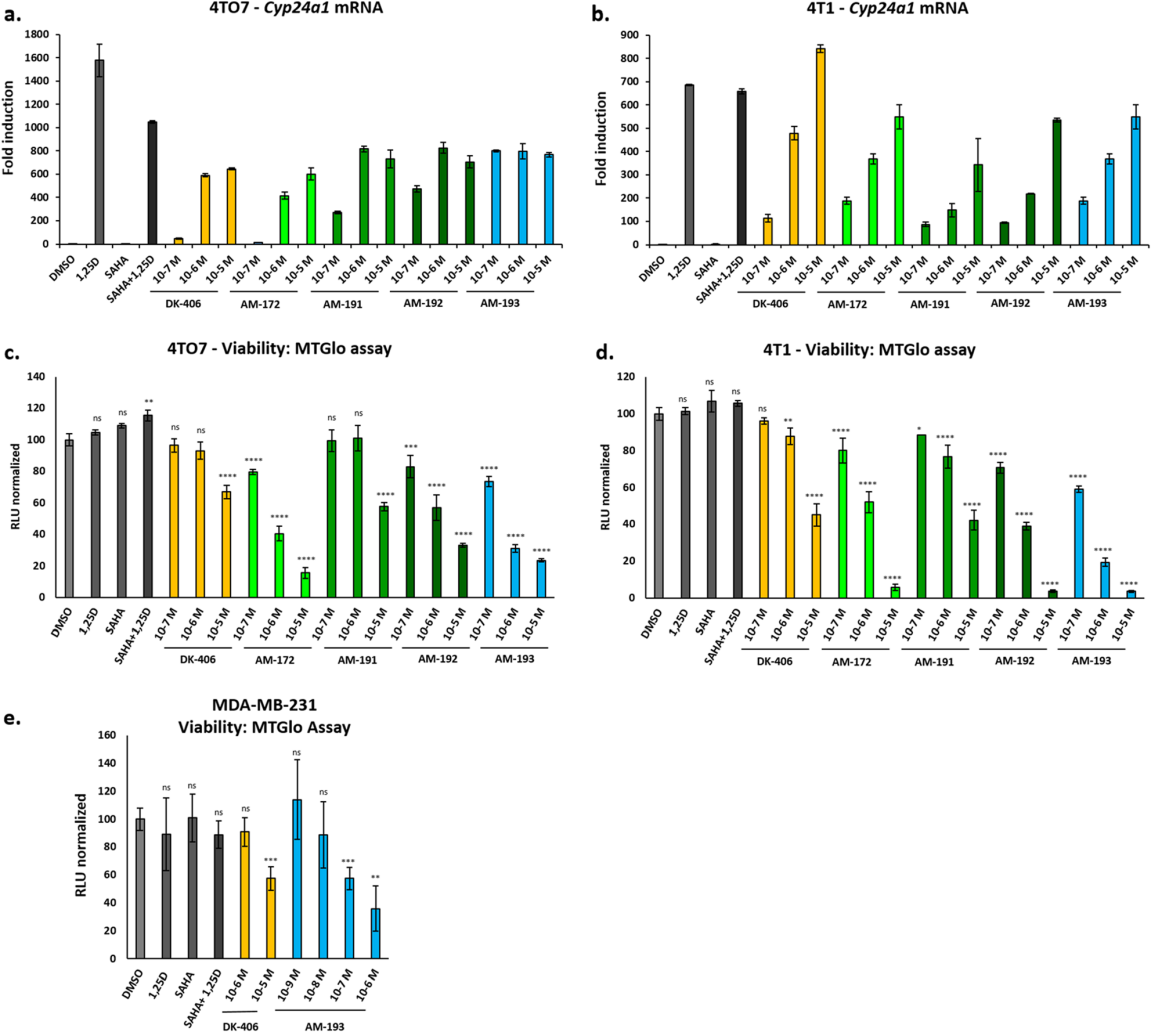
(**a**) and (**b**) Analysis of induced *Cyp24a1* expression in 4TO7 (**a**) and 4T1 (**b**) cells to test for VDR agonism of DK-406 and heterocyclic analogues after 24 h treatment. **c**-**e**. Analysis of the cytotoxicity of hybrid compounds after 24 h using the MTGlo assay (see Methods for details) in 4TO7 (**c**), 4T1 (**d**) and MDA-MB-231 (**e**) cells. 1,25D and SAHA were used at 10^–7^ M. Results are average of triplicates, bars are mean ± SD. Each condition was compared to control. *: *p* ≤ 0.05, **: *p* ≤ 0.01, ***: *p* ≤ 0.001, ****: *p* ≤ 0.0001, ns: not significant.

The hybrids were assessed to determine their HDACi and VDR agonist activity as well as their antiproliferative properties relative to DK-406. HDACi activity was assessed in vitro using an acetylated lysine substrate that releases a fluorescent reporter upon HDACi deacetylation and subsequent trypsin digestion^28^. Increasing sidechain length of pyrimidine hybrids enhanced the potency of HDAC6 inhibition, with several compounds inhibiting HDAC6 with a sub-micromolar IC_50_, similar to that of DK-406 (Fig. 4a). In contrast, pyridyl hybrids were somewhat less potent inhibitors of HDAC2 compared to HDAC6 and the normal dependence on chain length was disrupted. The hydroxamic acid sidechains of AM-193 and DK-406 are comparable in length. Previous studies showed that the relatively long side chain of DK-406 correlated with optimized HDACi activity but came at the cost of a modest loss of potency of VDR agonism^15^. Our current studies with heterocyclic hybrids showed that potency of VDR agonism improved with increasing side chain length. Next, we analyzed the effects of compounds on levels of H3K9 and H3K27 acetylation in 4TO7 cells, which are controlled by class I HDACs^29,30^. Effects of hybrids on H3 acetylation were compared to that of 100 nM SAHA, which produces almost complete inhibition of class I HDAC activity in vitro^31^. DK-406 induced H3 acetylation at K9 and K27 at 10^–5^ M (Fig. 4b,c) that was comparable to that of SAHA. As AM-193 was a less potent inhibitor than DK-406 of class I enzyme HDAC2 (Fig. 4a), we tested its activity at 10^–5^ M. Under these conditions, AM-193 enhanced H3 acetylation, although to a lesser degree than DK-406 (Fig. 4b,c). We also tested the capacity of analogues to induce tubulin acetylation, controlled by class II enzyme HDAC6^32^, in 4TO7 cells. Based on their relative efficacy in cytotoxicity assays (see below, Fig. 5c,d), we used 10^–5^ M DK-406 and 10^–6^ M AM-193 for these studies. In triplicate experiments, both analogues consistently induced tubulin acetylation (Fig. 4d; see Fig. S2 for full-sized blots).

As previously^20^, we were interested in studying the efficacy of hybrids in models of TNBC. We used the 4T1 cell line^20^ as well as the related TNBC cell line 4TO7, which was derived from the same mouse tumor as 4T1 cells in Balb/c mice^33^. 4TO7 cells readily form primary tumors. However, unlike 4T1 cells, which are aggressively metastatic, 4TO7 cells seed other organs but do not successfully establish metastases^33^. These lines also have the advantage that anti-tumor efficacy studies can be performed in Balb/c mice, which have intact immune systems. Compounds were initially tested for VDR agonist activity in 4TO7 cells by screening induction of the VDR target gene *Cyp24a1*. Compounds AM-155 and -171 (**17a** and **17b**) were weakly potent VDR agonists (Fig. S3a,b) and were not studied further. Subsequent experiments confirmed that potency of VDR agonism increased with the length of the hydroxamic acid sidechain and that AM-193 (**27c**) was approximately tenfold more potent in the *Cyp24a1* induction assay than DK-406 in 4TO7 cells (Fig. 5a). The potency of AM-193 was confirmed in related 4T1 cells (Fig. 5b). Notably, AM-193 and DK-406 appeared to be partial agonists in 4TO7 cells, whereas they functioned as full agonists in 4T1 cells (Fig. 5a,b). The pyridyl hybrids were then tested for efficacy in vitro in 4TO7 and 4T1 cells using an MT-Glo assay in which only viable cells can convert a precursor into a substrate for a *luciferase* bioluminescence assay (Fig. 5c,d; see Methods for details). AM-193 was the most potent compound tested and appeared to be at least tenfold more potent than DK-406 (Fig. 5c,d). In the same assay, the combination of a pharmacological concentration of 1,25D (1 µM) and 100 nM of the potent HDACi SAHA was not efficacious (Fig. 5c,d). The potency of AM-193 as a cytotoxic agent was also confirmed in the human TNBC cell line MB-MDA231 (Fig. 5e).

### Gene expression profiling by RNAseq of 1,25D, SAHA, 1,25D + SAHA, DK-406 and AM-193

**Figure 6:**
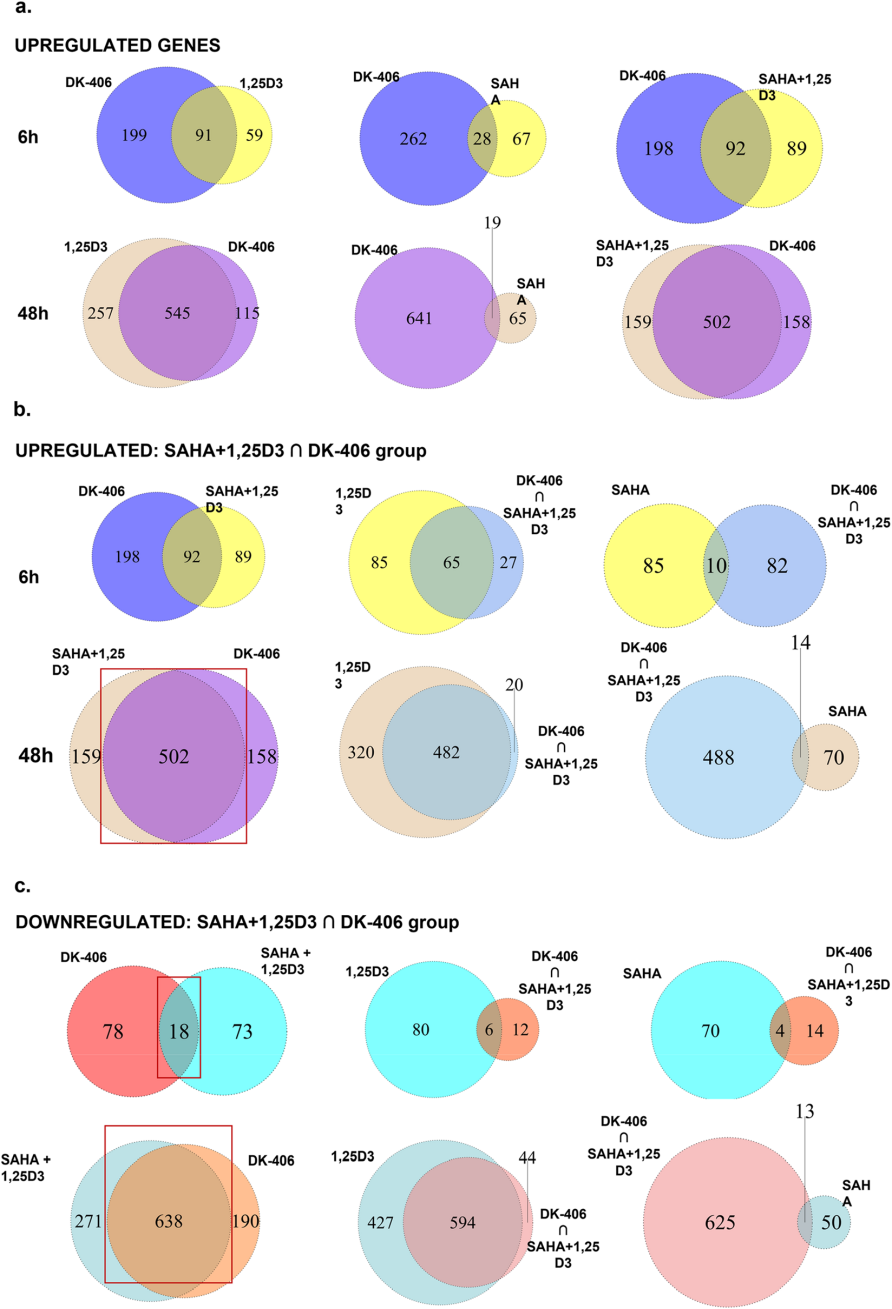
Analysis of the results of comparative RNAseq gene expression profiling studies of 4TO7 cells treated with vehicle, 1,25D, SAHA, 1,25D + SAHA and DK-406 for 6 or 48 h. (**a**) Venn diagrams representing overlaps in genes regulated by 1,25D, 1,25D + SAHA or SAHA alone and DK-406 in cells treated for 6 or 48 h, as indicated. (**b**) Analysis of the overlap between gene expression profiles in cells treated with DK-406 for 6 or 48 h with those treated with 1,25D + SAHA (left). The center and right Venn diagrams show the overlap between common genes regulated in DK-406- and 1,25D + SAHA-treated cells and genes regulated by 1,25D alone (center) or by SAHA alone (right). 1,25D or SAHA. (**c**) Same as in (**b**), except downregulated genes are shown.

To gain further insight into the mechanisms of action of the hybrid molecules we performed gene expression profiling by RNAseq in 4TO7 cells treated with vehicle, 1,25D, SAHA, SAHA + 1,25D, DK-406 and AM-193. 4TO7 cells were chosen for further study because they are less aggressively metastatic in vivo than 4T1 cells, and we are interested in future analyses of the efficacy of hybrids in preventing metastatic seeding the 4TO7 model. Cells were treated for 6 h to focus more on genes directly regulated by the VDR, or for 24 or 48 h to gain insight into longer-term effects on gene expression. An initial pilot study was performed in 4TO7 cells treated for 6 or 48 h with 1,25D and SAHA, alone or in combination, or with DK-406 (Supplemental Table 1). Of the 150 genes regulated by 1,25D after 6 h using the relatively stringent twofold cut-off, 91 overlapped with the 290 genes regulated by DK-406, with a similar degree of overlap observed between genes regulated by 1,25D + SAHA and DK-406 (Fig. 6a). There was a substantially lower degree of overlap between genes regulated by SAHA alone and DK-406 after 6 h (Fig. 6a). Remarkably, the overlap in gene regulatory profiles of cells treated with DK-406 and those exposed to 1,25D alone or with 1,25D + SAHA was substantially greater after 48 h than at 6 h, whereas that between DK-406 and SAHA alone was minimal (Fig. 6a). A similar trend in convergence of gene regulatory profiles at 48 h was observed when comparing the common genes regulated by DK-406 and 1,25D + SAHA with those regulated by 1,25D alone, whereas the overlap between the common genes and those regulated by SAHA was minimal (Fig. 6b,c). This data suggests that largely VDR-controlled gene regulation is driving the long-term gene expression profiles in cells treated with DK-406, as well as with 1,25D + SAHA.

**Figure 7:**
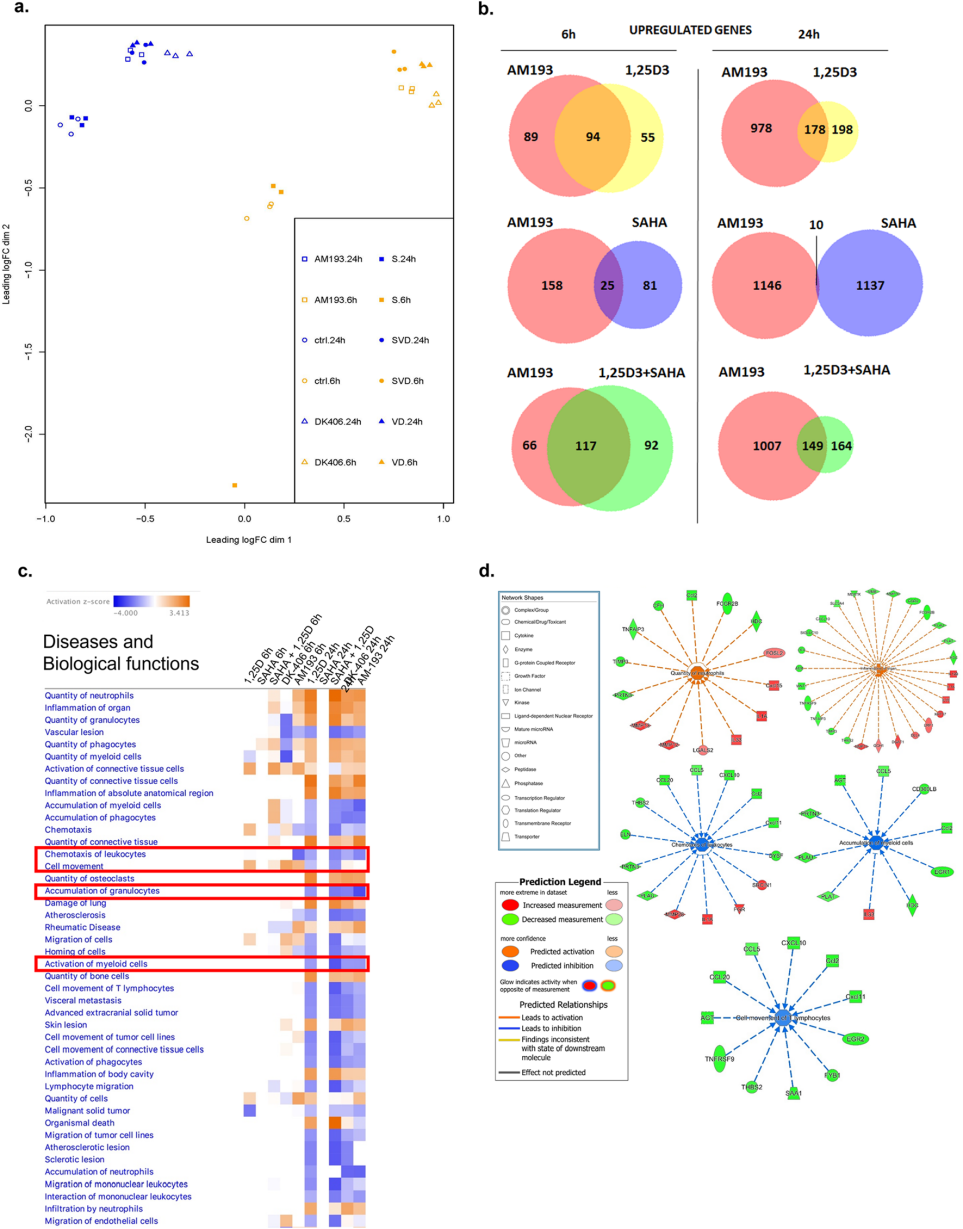
(**a**) Results of principal component analysis of gene expression profiles in 4TO7 cells treated with vehicle (ctl), AM-193, DK-406, SAHA (S), 1,25D (VD) or SAHA + 1,25D (SVD) for 6 or 24 h. (**b**) Venn diagrams showing overlaps in expression profiles of upregulated genes in 4TO7 cells treated with AM-193, 1,25D, SAHA or 1,25D + SAHA for 6 or 24 h. (**c**) Top enriched diseases and biological functions following 6 and 24 h treatment with 1,25D, 1,25D + SAHA, DK-406 or AM-193. Diseases and functions with a predicted activation state (positive z-score) are labeled in orange while those with a predicted inhibition state (negative z-score) are labeled in blue. All diseases and functions have a log *p*-value cut-off of 1.3. Enclosed in red rectangles are diseases and biological functions that are of interest. (**d**) Selected diseases and biological functions that are predicted to be activated (positive z-score) and inhibited (negative z-score) subsequent to 24 h treatment with AM-193. Each function is displayed as nodes (genes) and edges (biological relationship between nodes). The colour intensity of each node signifies log fold change expression; red and green represent upregulated and downregulated genes, respectively. Edges connecting the genes to the respective functions indicate the predicted relationships. The colour of the central function and edges indicate activation (orange) or inhibition (blue) of the different functions. Inconsistent findings and effects that are not predicted are not illustrated for clarity.

To substantiate this result, a similar RNAseq study was conducted in 4TO7 cells treated for 6 or 24 h, which included AM-193 as well as DK-406, 1,25D and/or SAHA (Supplemental Table 3). Because of their differences in potency as cytostatic and cytotoxic agents (Fig. 6 above), cells were treated with 1 µM AM-193 and 10 µM DK-406. Principal component analysis of results from 6 h time points showed a remarkable clustering of results from cell treated with 1,25D, 1,25D + SAHA, DK-406 or AM-193 (Fig. 7a), whereas profiles from vehicle or SAHA-treated cells were similar (with the exception of one outlier of the SAHA-treated samples). This result is consistent with the relatively limited effects of SAHA alone on gene expression in 4TO7 cells, above. A similar clustering of results was observed in cells treated with vehicle or SAHA for 24 h (Fig. 7a). In contrast, PCA analysis clustered results from cells treated for 24 h with 1,25D, 1,25D + SAHA, DK-406 or AM-193 in a group distinct from vehicle-treated cells and distinct from that generated from the profiles obtained at 6 h (Fig. 7a). Venn diagrams of these results illustrate the substantial overlap in genes regulated by hybrids, 125D and 1,25D + SAHA (Fig. 7b).

Previous microarray studies in human myelomonocytic cells with the HDACi trichostatin A (TSA) and 1,25D showed that short-term treatment (90 min) with TSA had widespread effects on gene expression and substantially alter 1,25D-regulated gene expression^34^. Based on these findings, the relatively modest effects we observed in our two studies on gene expression in 4TO7 cells exposed to SAHA were unexpected, given that SAHA and TSA are broad-spectrum HDACi. However, our studies and the previous one differ in the cell lines used, and the duration of the treatments (90 min vs 6, 24 or 48 h). Although there was only partial overlap between the genes regulated by DK-406 and 1,25D alone or with SAHA after 6 h, there was substantial convergence in their gene expression profiles after 48 h. This suggests that long-term effects on gene expression by hybrids are very similar to those driven by 1,25D. The results are also consistent with our observations that, although AM-193 is more potent than DK-406 as a cytostatic and cytotoxic agent, notably acting at 10^–6^ M, it was less efficacious than DK-406 at inducing H3 acetylation at 10^–5^ M (Fig. 5c,d, above). This raises the possibility that any therapeutic effect of the HDACi component of hybrids may be through inhibition of HDACs that do not directly control gene expression. There are several potential targets of HDACi that bind to zinc metalloenzymes (HDACs 1–11; class I, II and IV enzymes), and many HDACs, particularly class II enzymes are partially or wholly cytoplasmic^12^. Consistent with this notion, after 48 h SAHA alone only modestly altered gene expression (84 genes regulated at east twofold), of which only 19 overlapped with the 660 genes whose expression was induced at least twofold by DK-406.

### Pathways analysis of regulated gene expression

We used Ingenuity Pathway Analysis (QIAGEN) to identify enriched diseases and biological functions in our datasets (Fig. 7c). Interestingly, this analysis revealed that 1,25D or hybrid treatments influenced quantities of neutrophils and phagocytes as well as inflammation; however, pathways analysis predicted that accumulations of myeloid cells, phagocytes, granulocytes and chemotaxis are inhibited. In cells treated with AM-193 for 24 h, *Quantity of neutrophils* (Z-score = 2.423 *p* = 1.37E-07) and *Inflammation of organ* (Z-score = 1.878, *p* = 1.77E-10) are predicted to be in an activated state, whereas *Chemotaxis of leukocytes* (Z-score = − 1.912, *p* = 3.46E−09), *Accumulation of myeloid cells* (Z-score = − 2.470, *p* = 2.23E−08) and *Cell movement of T lymphocytes* (Z-score = − 1.258, *p* = 1.35E−06) are predicted to be inhibited (Fig. 7d). Additional activated and inhibited functions include *Quantity of phagocytes* (Z-score = 1.613, *p* = 3.44E−08) and *Quantity of granulocytes* (Z-score = 1.832, *p* = 6.60E−08) as well as *Accumulation of phagocytes* (Z-score = − 2.061, *p* = 6.10E−08) and *Accumulation of granulocytes* (Z-score = − 2.800, *p* = 5.46E−07) (Fig. S4a,b).

Most of the genes in these pathways encode secreted proteins. The expression of several matrix metalloproteinases was elevated in cells treated with 1,25D or hybrids, enzymes that have been implicated in cancer progression^35^. In contrast, the expression of numerous cytokines and chemokines was suppressed. These include Ccl2, Ccl5, Ccl20, Cxcl10, and Cxcl11, all of which have been implicated in promoting breast tumor growth or metastasis^36–42^. Conversely, expression of the gene encoding Il12b was induced. Elevated Il-12 levels in the breast tumor microenvironment promote antitumor immunity and correlate with reduced metastasis^43,44^. Notably, Ccl2, whose expression is repressed, promotes recruitment of M2 type macrophages, which facilitate tumor progression^37^. Similarly, expression of the genes encoding complement factor H (Cfh) and Siglec10 (Cd24), both of which can promote tumor immune escape in breast cancer^45,46^, were inhibited. Collectively, these results suggest that hybrids would alter the immune infiltrate composition and phenotype, suppressing tumor progression, and possibly promoting anti-tumor immunity.

**Figure 8:**
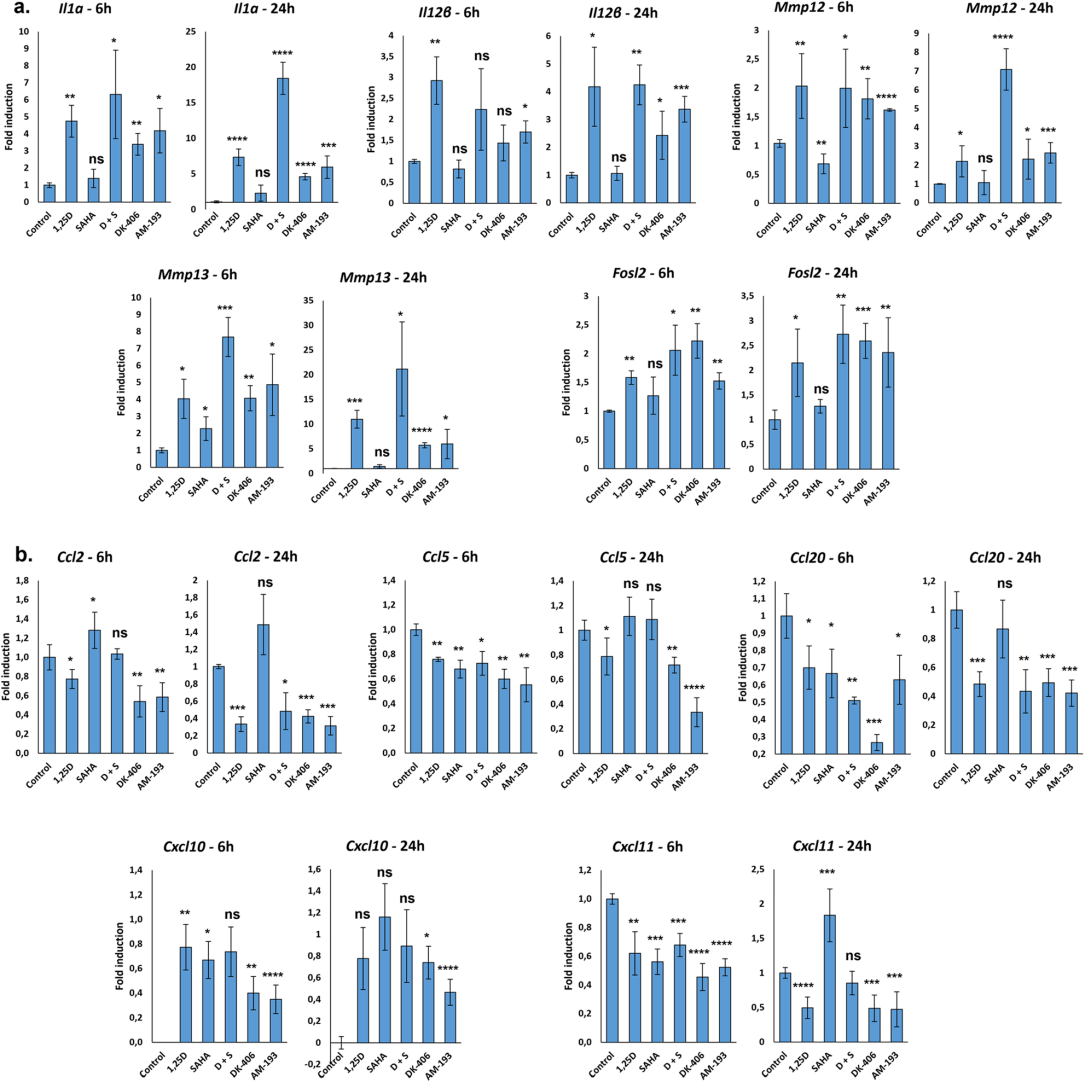
(**a**, **b**) Validation of the results of the regulation of selected upregulated (**a**) and downregulated genes (**b**) identified in RNAseq studies performed in 4TO7 cells treated with vehicle, 1,25D (10^–7^ M), SAHA (10^–7^ M), 1,25D + SAHA, DK-406 (10^–5^ M) or AM-193 (10^–6^ M) for 6 and 24 h, as indicated. Each condition was compared to control. *: *p* ≤ 0.05, **: *p* ≤ 0.01, ***: *p* ≤ 0.001, ****: *p* ≤ 0.0001, ns: not significant.

In addition, several genes that are downregulated encode secreted factors whose expression correlates with breast cancer metastasis, including adrenomedullin (Adm)^47,48^, urokinase-type plasminogen activator (Plaur, uPA)^49^ and the secreted protease Prtn3^50^. Finally, we note that treatment with hybrids suppressed expression of Slc2A4, which encodes the glucose transporter Glut 4, whose loss impairs the viability of triple-negative breast tumors^51^. To validate these results, we selected ten target genes with differing fold up and down regulations for further study by RT/qPCR using RNA isolated from 4TO7 cells (Fig. 8a,b). The results broadly confirm the gene regulatory events seen in RNAseq experiments. Taken together, the results are consistent with gene expression profiles generated in AM-193 or DK-406-treated cells being driven largely by their capacity to function as VDR agonists. They also suggest that exposure to hybrids will lead to profound changes in the cellular composition of the tumor microenvironment.

## Methods

### Reagents

1α,25-Dihydroxyvitamin D3 (BML-DM200) was purchased from Enzo Life Sciences and was used at a final concentration of 100 nM. Suberoylanilide hydroxamic acid (SAHA, #10009929)) was purchased from Cayman Chemical and was used at a final concentration of 100 nM.

### Cell culture

Mouse 4T07 cells and 4T01 cells were cultured in DMEM (319–005-CL; WISENT Inc.) supplemented with 10% heat-inactivated FBS. MB-MDA231 cells were cultured in Leibovitz’s L-15 medium with 10% heat-inactivated FBS in the absence of CO_2_. Before treatments, cells were split and 24 h later, DMSO (vehicle), 1,25D, SAHA, DK-406 or AM-193 was added to the media for 6 or 24 h.

### RealTime-Glo MT cell viability assay

The assay was performed to assess cell viability in order to determine analogue cytotoxicity. After treatment of 4TO7, 4T1 or MB-MDA231 cells for 24 h, the assay was performed according to the manufacturer’s instructions (Promega – #G9712). Luminescence was measured using a 1420 Luminescence Counter Victor Light (PerkinElmer), normalized to control, and plotted. All samples were run in triplicate.

### Fluorogenic HDAC inhibition assay

Purified HDAC2 and HDAC6 were purchased from Cayman Chemical. Boc-Lys(Ac)-7-amino-4-methylcoumarin (BocLys(Ac)-AMC) was used as substrate for the HDAC assays. Substrate solution was prepared as follow: Boc(Lys-Ac)-AMC was dissolved in DMSO and diluted with HDAC buffer (15 mM tris–HCl [pH 8.1], 250 μM EDTA, 250 mM NaCl, 10% glycerol) to give 1 mM solutions containing 1.7% DMSO. Trypsin was used to stop the reaction, releasing free AMC. The trypsin solution was prepared as follow: trypsin was dissolved in HDAC buffer to give a concentration of 10 mg/mL. Release of AMC was monitored by measuring the fluorescence at 460 nm (lex = 390 nm) with a microplate reader (SpectraMax Gemini from Molecular Devices) at 37 °C. The AMC signals were recorded against a blank with buffer, substrate and trypsin but without the enzyme. All experiments were carried out at least in triplicate.

For HDAC inhibition assays, inhibitor diluted in 50 μL of HDAC buffer was mixed with 10 μL of diluted enzyme solution in HDAC buffer at room temperature. The HDAC reaction was started by adding 40 μL of substrate solution in HDAC buffer followed by 60 min of incubation with stirring at 37 °C. The reaction was stopped by adding 100 μL of trypsin solution. After a 30 min incubation with stirring at 37 °C, the release of AMC was monitored by measuring the fluorescence.

### RNA sequencing

4TO7 cells were prepared in triplicate and RNA were extracted after 6 h, 24 h or 48 h treatment (1,25D, SAHA, 1,25D + SAHA, DK-406, AM-193 or vehicle). After RNA extraction, all RNA had a concentration higher than 100 ng/µL and a ratio 260/280 around 2, they were sent to McGill Genome Centre. McGill Genome Centre performed a quality control before sequencing in paired-end at 50 M reads by Illumina NovaSeq 6000 S4 PE100. Sets of genes regulated in the different conditions were sent to us and downstream analysis were performed.

### RNA extraction, reverse transcription and qPCR

RNA extraction was performed with the FavorPrep tissue total RNA mini kit (FAVORGEN Biotech Corporation—FATRK 001) as per manufacturer’s instructions. cDNA was obtained from 1 µg of RNA using 5X All-in-One RT Mastermix (Applied Biological Materials (abm) Inc.—G485) and diluted 5 times. qPCR was performed with BrightGreen 2X q-PCR MasterMix (abm—MasterMix-LR-XL) on a Roche LightCycler 96 system. Expression of targeted genes was normalized to 18S RNA. All primers are listed in Table S3.

### Western Blotting and protein analysis

After treatments, 4T07 cells were solubilized in Lysis Buffer (20 mM Tris, pH 8, 150 mM NaCl, 1% Triton X-100, 3,5 mM SDS, 13 mM deoxycholic acid) and proteins extracted were separated on a 4–15% Tris/Glycine/SDS gel (BIO RAD). A standard protocol was used for transfer and blotting. Anti-acetylated tubulin antibody was purchased from Sigma-Aldrich (#T7451). Anti-H3 antibody was clone96C10 (Cell Signaling Technology (CST); cat # 3638). Anti-acetyl-histone H3(Lys9) was 07–352 (Merck Millipore), and anti-acetyl-histone H3 (Lys27) was #8173 (CST). Anti-GAPDH antibody was purchased from Abcam (#ab8245) and the anti-mouse IgG HRP-linked secondary antibody was purchased from Cell Signaling Technology and were used at recommended concentrations. Signal was detected using Clarity ECL chemiluminescent substrates (Bio-Rad) and ChemiDoc Imaging System from the same company was used to quantify the intensity of bands. Changes in protein levels were quantified relative to control using Image Lab software (Version 6.0.1) after normalization to GAPDH. Western-blot images presented are three biological replicates.

### Ingenuity pathway analysis

Lists of differentially expressed transcripts with a *p* value of ≤ 0.05 and a twofold cut-off for each comparison were analyzed with Ingenuity Pathway Analysis or IPA (QIAGEN Inc., https://www.qiagenbioinformatics.com/products/ingenuitypathway-analysis) to identify enriched diseases and biological functions. IPA uses Fisher’s exact test to determine a probability value to display the association between each gene in the list and IPA-curated pathways and biological functions. A log p-value cut-off of 1.3 was considered statistically significant overrepresentation of genes in a disease or biological function. Furthermore, IPA employs a z-score of greater than 2 and less than − 2 to indicate significant predicted activation and inhibition states of functions, respectively.

### Statistics

Student’s *t*-test were performed using GraphPad software (Version 8.0.1).

## Supplementary Information

Supplementary Information 1.

Supplementary Information 2.

Supplementary Information 3.

Supplementary Information 4.
