# Supplementary material for: Molecular mechanisms of bifunctional vitamin D receptor agonist-histone deacetylase inhibitor hybrid molecules in triple-negative breast cancer
Source: Sci Rep. 2022 Apr 25;12:6745. doi: 10.1038/s41598-022-10740-9 (PMC9038752; doi:10.1038/s41598-022-10740-9)
Supplement: Supplementary file 1 — Supplementary Information 1. [file 41598_2022_10740_MOESM1_ESM.pdf]

Supporting Information

Molecular mechanisms of bifunctional vitamin D receptor agonist-histone deacetylase inhibitor hybrid molecules in triple-negative breast cancer

Camille Barbier[1], Ali Mansour[2], Aiten Ismailova[1], Camille Zeitouni[1], Manuella Bouttier[1], David Scarlata[2], James L. Gleason[2]* and John H. White[1,3]*

Departments of Physiology[1], Chemistry[2] and Medicine[3], McGill University, Montréal, QC, Canada.

To whom correspondence should be addressed: jim.gleason@mcgill.ca; john.white@mcgill.ca

Contents

1. Chemical synthesis and experimental procedures Page 2

Materials:

Tetrahydrofuran and diethyl ether were purified by distillation over sodium metal and benzophenone under a nitrogen atmosphere. Toluene, dichloromethane, and triethylamine were purified by distillation over calcium hydride under a nitrogen atmosphere. *N,N*-dimethylformamide and MeOH were stored over activated 3 Å molecular sieves and kept under an argon atmosphere. *n*-BuLi was titrated with *sec*-butanol in toluene using 2,2'-bipyridine as an indicator. All other commercial solvents and reagents were used as received unless otherwise specified. Normal-phase flash-column chromatography was performed using SiliaFlash® P60 Ultrapure silica (particle size: 40-63 μm, 230-400 mesh) obtained from SiliCycle and was used as received. Reversed-phase flash-column chromatography was performed using octadecyl-functionalized silica obtained from Sigma-Aldrich and was used as received. TLC was performed on glass-backed Ultrapure silica TLC plates (extra hard layer, 60 Å, thickness: 250 μm) obtained from SiliCycle, visualized with a Spectroline UV254 lamp, and stained with acidic ceric ammonium molybdate (CAM) solution, basic potassium permanganate solution, acidic *p*-anisaldehyde solution, or acidic iron (III) chloride solution. All reactions were performed in oven-dried or flame-dried round bottom flasks fitted with a rubber septum under a positive pressure of argon with magnetic stirring, unless otherwise stated. Liquids and solutions were transferred via syringe or stainless-steel cannula.

Instrumentation:

^{1}H and ^{13}C NMR, recorded at 400 MHz and 100 MHz, respectively, were performed on a Varian Mercury and Bruker 400 spectrometer. ^{1}H and ^{13}C NMR, recorded at 500 MHz and 125 MHz, respectively, were performed on a Varian Mercury and Bruker 500 spectrometer. Proton chemical shifts were internally referenced to the residual proton resonance in $CDCl_3$ (δ 7.26 ppm), CD_3OD (δ 3.31 ppm), and d_6-DMSO (δ 2.50 ppm). Carbon chemical shifts were internally referenced to the deuterated solvent signals in CDCl3 (δ 77.2 ppm), CD_3OD (δ 49.0 ppm), and d_6-DMSO (δ 39.50 ppm). Coupling constants (*J*) are reported in Hertz (Hz). LC-MS analysis was performed using a Waters Alliance® instrument (e2695 with 2489 UV detector and 3100 mass spectrometer) using a mobile phase of H_2O:acetonitrile with 0.1% formic acid. The linear gradient used is from 95% H_2O:5% acetonitrile to 100% acetonitrile in 15 min with a flow rate of 1 mL/min. High-resolution mass spectrometry (HRMS) was performed by Dr. Nadim Saadé and Dr. Alexander Wahba at the McGill University Mass Spectrometry Facility in the Department of Chemistry, using electrospray ionization and chemical ionization techniques.

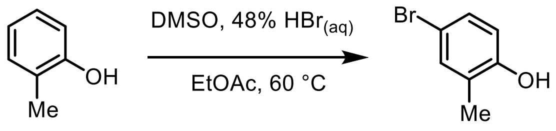

4-bromo-2-methylphenol (8): HBr (48% v/v solution in H_2O, 3.5 mL, 65 mmol, 2.0 equiv) and DMSO (2.5 mL, 36 mmol, 1.1 equiv) were added drop-wise to a stirring solution of *o*-cresol **7** (3.3 mL, 32 mmol, 1 equiv) at 60 °C in EtOAc (324 mL) under air and stirred overnight. After completion of the reaction (as judged by crude NMR), the reaction was diluted with 200-mL of a brine solution and extracted with three 200 mL volumes of EtOAc. The organic fractions were dried over anhydrous $MgSO_4$, filtered, and concentrated *in vacuo* to afford a dark brown liquid/oil. The residue was purified by chromatography on silica gel using 5–10% Et_2O:petroleum Et_2O as eluent to provide bromophenol **8** as a white solid (4.2 g) in 72% yield. The spectroscopic data is in agreement with that published in the literature[1].

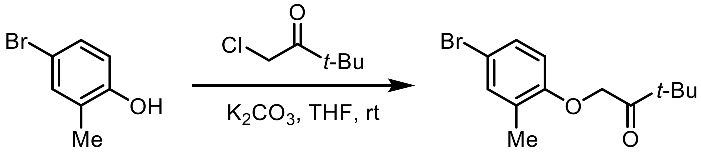

1-(4-bromo-2-methylphenoxy)-3,3-dimethylbutan-2-one (9): 1-chloropinacolone (6.6 mL, 44 mmol, 1.5 equiv), freshly filtered through a plug of basic alumina, was added in one portion to a vigorously stirred slurry of bromophenol **8** (6.3 g, 34 mmol, 1 equiv) and solid K_2CO_3 (9.3 g, 67 mmol, 2.0 equiv) in THF (168 mL) under air. The reaction mixture was heated at reflux overnight, at which point TLC analysis indicated complete consumption of the starting material. The reaction mixture was allowed to cool to room temperature and filtered over a pad of celite *via* vacuum filtration. The filtrate was collected as a yellow solution and concentrated to a dark-yellow oil. The residue was purified by chromatography on silica gel using 2.5–5% EtOAc:hexanes as eluent to provide ketone **9** as a clear oil (8.2 g) in an 86% combined yield. R_f 0.30 (10% EtOAc:hexanes); ^{1}H NMR (400 MHz, $CDCl_3$) δ 7.26 (d, *J* = 1.9 Hz, 1H), δ 7.19 (dd, *J* = 8.6, 2.3 Hz, 1H), δ 6.47 (d, *J* = 8.6 Hz, 1H), δ 4.85 (s, 2H), δ 2.27 (s, 3H), δ 1.24 (s, 9H). ^{13}C NMR (125 MHz, $CDCl_3$) δ 209.29, 155.48, 133.59, 129.72, 129.25. 113.31, 112.69, 69.32, 43.17, 26.32, 16.19. **Note:** 1-chloropinacolone co-eluted with the product and was difficult to remove, appearing at δ 4.37 (s, 2H) and δ 1.21 (s, 9H). Fortunately, the two were easily separable in the next step. HRMS (APCI) calculated for $C_{13}H_{18}O_2Br$ $[M + H]^+$: 285.04847, found 285.04875.

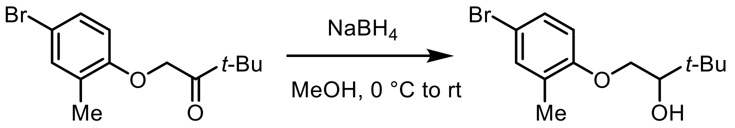

9 **10**

1-(4-bromo-2-methylphenoxy)-3,3-dimethylbutan-2-ol (10): $NaBH_4$ (2.6 g, 67 mmol, 2.0 equiv) was added in portions to a 0 °C solution of ketone **9** (9.6 g, 34 mmol, 1 equiv) in MeOH (337 mL) under air. The reaction mixture was taken out of the ice bath and warmed to room temperature and stirred for one to two hours. The reaction mixture was subsequently cooled to 0 °C and quenched (slowly) with 200-mL of a 1 M HCl solution or until all gas evolution had ceased. The reaction mixture was concentrated to remove most of the MeOH and was extracted with EtOAc (3x 200 mL), followed by a 100-mL wash with brine solution. The organic fractions were dried over anhydrous $MgSO_4$, filtered, and concentrated to afford a yellow, murky white viscous oil. The residue was purified by chromatography on silica gel using 25–100% DCM:hexanes as eluent to provide secondary alcohol **10** as a clear, yellow tinted oil (5.4 g) in 66% yield. R_f 0.29 (10% EtOAc:hexanes); ^{1}H NMR (400 MHz, $CDCl_3$) δ 7.28–7.21 (m, 2H), δ 6.68 (d, J = 8.3 Hz, 1H), δ 4.07 (dd, J = 9.1, 2.6 Hz, 1H), δ 3.84 (t, J = 8.9 Hz, 1H), δ 3.71 (d, J = 8.6 Hz, 1H), δ 2.34 (s, 1H), δ 2.20 (s, 3H), δ 1.01 (s, 9H). ^{13}C NMR (125 MHz, $CDCl_3$) δ 155.90, 133.38, 129.44, 129.10, 112.92, 112.78, 77.26, 69.76, 33.64, 26.06, 16.17. HRMS (ESI) calculated for $C_{13}H_{19}BrO_2Na$ [M + Na]$^+$: 309.04606, found 309.04564.

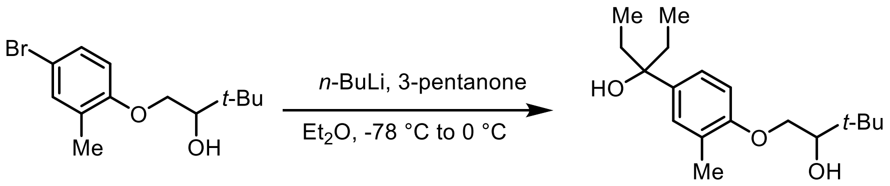

10 **11**

3-(4-(2-hydroxy-3,3-dimethylbutoxy)-3-methylphenyl)pentan-3-ol (11): *n*-BuLi (2.5 M solution in hexanes, 18 mL, 39 mmol, 2.2 equiv) was added drop-wise over a period of 10 min to a stirring solution of secondary alcohol **10** (5.1 g, 18 mmol, 1 equiv) at -78 °C in anhydrous Et_2O (94 mL). The reaction mixture was allowed to stir for two hours, at which point the reaction mixture was warmed to 0 °C and 3-pentanone (2.1 mL, 19 mmol, 1.1 equiv) was added in one portion and the reaction mixture was stirred for another hour. After the one hour, the reaction mixture was quenched with a 50-mL of a saturated solution of NH_4Cl and extracted with Et_2O (3x 100 mL). The organic fractions were dried over anhydrous $MgSO_4$, filtered, and concentrated to afford a yellow oil. The residue was purified by chromatography on silica gel using 10–15%

EtOAc:hexanes as eluent to provide tertiary alcohol **11** as a yellow, viscous oil (2.8 g) in 53% yield. R_f 0.22 (15% EtOAc:hexanes); ^{1}H NMR (400 MHz, $CDCl_3$) δ 7.16–7.11 (m, 2H), δ 6.77 (d, *J* = 9.1 Hz, 1H), δ 4.10 (dd, *J* = 9.1, 2.6 Hz, 1H), δ 3.87 (t, *J* = 8.9 Hz, 1H), δ 3.71 (dt, *J* = 8.7, 2.7 Hz, 1H), δ 2.44 (d, *J* = 2.9 Hz, 1H), δ 2.24 (s, 3H), δ 1.88–1.71 (m, 4H), δ 1.58 (s, 1H), δ 1.01 (s, 9H), δ 0.75 (t, *J* = 7.4 Hz, 6H). ^{13}C NMR (125 MHz, $CDCl_3$) δ 155.16, 138.02, 128.10, 126.06, 123.86, 110.54, 77.31, 77.09, 69.36, 34.89, 33.60, 26.08, 16.62, 7.88.

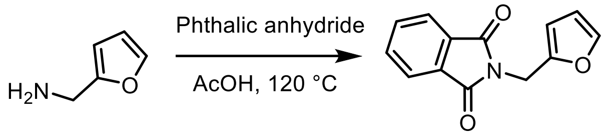

12

2-(furan-2-ylmethyl)isoindoline-1,3-dione (12): Phthalic anhydride (3.9 g, 27 mmol, 1.2 equiv) was added in one portion to a stirring solution of furfurylamine (2.1 mL, 22 mmol, 1 equiv) in neat AcOH (22 mL) under air. The reaction mixture was brought to reflux and stirred for two hours before being neutralized to pH 7 using solid $NaHCO_3$ in portions (caution: gas evolution). The now black reaction mixture was extracted with EtOAc (3x 50 mL) followed by a 25-mL wash with a brine solution. The organic fractions were dried over anhydrous $MgSO_4$, filtered, and concentrated to afford a black-brown solid. The residue was purified by chromatography on silica gel using 15% EtOAc:hexanes as eluent to provide furfuryl phthalimide **12** as a white solid (4.7 g) in 94% yield. The spectroscopic data is in agreement with that published in the literature[2].

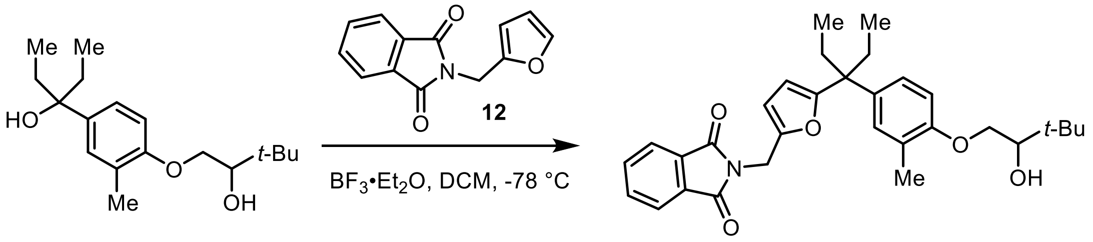

11 **13**

2-((5-(3-(4-(2-hydroxy-3,3-dimethylbutoxy)-3-methylphenyl)pentan-3-yl)furan-2-yl)methyl)isoindoline-1,3-dione (13): $BF_3·Et_2O$ (1.1 mL, 9.1 mmol, 1.1 equiv) was added drop-wise over several minutes to a -78 °C solution of tertiary alcohol **11** (2.4 g, 8.3 mmol, 1 equiv) and furfuryl phthalimide **12** (2.5 g, 11 mmol, 1.3 equiv) in anhydrous DCM (42 mL). After two hours, the dark purple reaction mixture was quenched with 100-mL of a saturated solution of $NaHCO_3$ and left to warm to room temperature and stir for an additional 10–15 min. The now orange solution was extracted with DCM (3x 75 mL), followed by a 30-mL wash with brine

solution. The organic fractions were dried over anhydrous $MgSO_4$, filtered, and concentrated. The residue was purified by chromatography on silica gel using 100% DCM:hexanes as eluent to elute excess **12**, followed by 25% EtOAc:hexanes as eluent to provide phthalimide **13** as a yellow, viscous oil (3.0 g) in 73% yield. R_f 0.56 (5% Et_2O:DCM); ^1H NMR (400 MHz, $CDCl_3$) δ 7.87–7.76 (m, 2H), δ 7.69–7.63 (m, 2H), δ 6.90–6.82 (m, 2H), δ 6.60 (d, J = 8.4 Hz, 1H), δ 6.16 (d, J = 2.8 Hz, 1H), δ 5.98 (d, J = 3.0 Hz, 1H), δ 4.73 (s, 2H), δ 4.00 (dd, J = 9.1, 2.1 Hz, 1H), δ 3.77 (t, J = 8.9 Hz, 1H), δ 3.67–3.61 (m, 1H), δ 2.38 (d, J = 2.4 Hz, 1H), δ 2.07 (s, 3H), δ 2.02–1.83 (m, 4H), δ 0.96 (s, 9H), δ 0.58 (t, J = 7.3 Hz, 6H). ^{13}C NMR (125 MHz, $CDCl_3$) δ 167.50, 160.74, 154.57, 147.56, 137.43, 133.94, 132.10, 129.84, 125.68, 125.46, 123.31, 110.33, 108.24, 107.26, 69.20, 47.21, 34.79, 33.57, 28.63, 26.08, 16.52, 8.41.

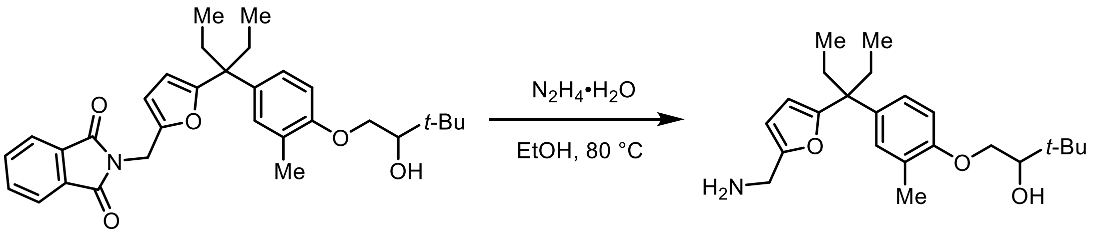

13 **14**

1-(4-(3-(5-(aminomethyl)furan-2-yl)pentan-3-yl)-2-methylphenoxy)-3,3-dimethylbutan-2-ol (14): Hydrazine monohydrate (4.4 mL, 91 mmol, 15 equiv) was added in one portion to a stirring solution of phthalimide **13** (3.0 g, 6.0 mmol, 1 equiv) in EtOH (30 mL) and brought to reflux for one hour, at which point a white precipitate began to crash out of solution, signaling the end of the reaction. The reaction mixture was allowed to cool to room temperature, and the white precipitate was dissolved using 20-mL of a 1 M NaOH solution. The reaction mixture was extracted with EtOAc (3x 50 mL) and the organic fractions were dried over anhydrous Na_2SO_4, filtered, and concentrated to afford 1.6 g (73%) of primary amine **14** as a yellow, viscous oil which was sufficiently pure for use without further purification. R_f 0.14 (100% EtOAc); ^1H NMR (400 MHz, $CDCl_3$) δ 6.96–6.89 (m, 2H), δ 6.71 (d, J = 9.2 Hz, 1H), δ 6.08 (d, J = 3.0 Hz, 1H), δ 6.03 (d, J = 3.0 Hz, 1H), δ 4.08 (dd, J = 9.1, 2.6 Hz, 1H), δ 3.85 (t, J = 8.9 Hz, 1H), δ 3.72–3.65 (m, 3H), δ 2.18 (s, 3H), δ 2.09–1.89 (m, 5H), δ 1.00 (s, 9H), δ 0.66 (t, J = 7.3 Hz, 6H). ^{13}C NMR (125 MHz, $CDCl_3$) δ 159.68, 154.70, 151.87, 137.85, 129.92, 125.58, 110.38, 107.24, 106.27, 77.28, 69.28, 48.68, 47.20, 33.58, 28.63, 26.07, 16.63, 8.42. HRMS (ESI) calculated for $C_{23}H_{35}NO_3Na$ $[M + Na]^+$: 396.25092, found 396.25015. **Note:** Molecular ion (M^+) peak is very small due to fragmentation, producing a major peak with m/z = 357.24227 (loss of NH_2).

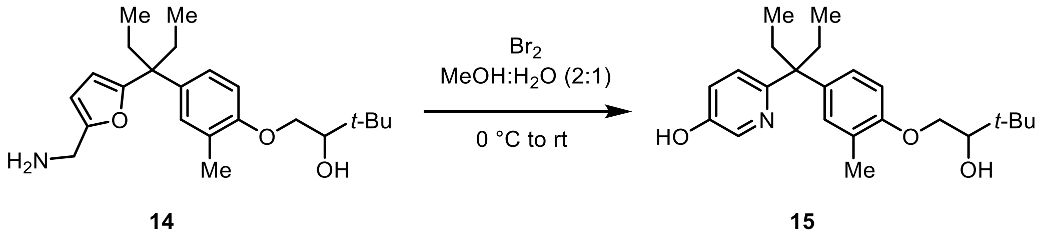

6-(3-(4-(2-hydroxy-3,3-dimethylbutoxy)-3-methylphenyl)pentan-3-yl)pyridin-3-ol (15): Bromine (10 μL, 0.19 mmol, 1.2 equiv) was added all in one portion to a vigorously stirring solution of primary amine **14** (60 mg, 0.16 mmol, 1 equiv) at 0 °C in a 2:1 mixture of MeOH:H_2O (1.6 mL) under air. The dark orange reaction mixture was subsequently warmed to room temperature and stirred for a minimum of 18 hours, at which point the reaction mixture was quenched with 2-mL of a saturated $Na_2S_2O_3$ solution and extracted with EtOAc (3x 15 mL). The organic fractions were dried over anhydrous Na_2SO_4, filtered, and concentrated to afford an orange-brown foam. The residue was purified by chromatography on silica gel using 40% EtOAc:hexanes as eluent to provide hydroxy pyridine **15** as a brown solid (37 mg) in 62% yield. R_f 0.50 (50% EtOAc:hexanes); ^{1}H NMR (400 MHz, $CDCl_3$) δ 8.11 (s, 1H), δ 7.06–6.83 (m, 4H), δ 6.69 (d, *J* = 8.5 Hz, 1H), δ 4.07 (dd, *J* = 9.1, 2.5 Hz, 1H), δ 3.84 (t, *J* = 9.0 Hz, 1H), δ 3.68 (dd, *J* = 8.8, 2.5 Hz, 1H), δ 2.26–2.07 (m, 7H), δ 0.99 (s, 9H), δ 0.60 (t, *J* = 7.3 Hz, 6H). HRMS (ESI) calculated for $C_{23}H_{34}NO_3Na$ [M + H]$^+$: 372.25332, found 372.25309.

General Procedure A: Alkylation of hydroxy pyridine 15 and phenol 25

Alkyl halide (1.3 equiv), freshly filtered through a plug of basic alumina, was added in one portion to a vigorously stirred slurry of hydroxy pyridine **15** or phenol **25** (1 equiv) and solid K_2CO_3 (2.0 equiv) in ACN under air. The reaction mixture was heated at reflux overnight, at which point TLC analysis indicated complete consumption of the starting material. The reaction mixture was allowed to cool to room temperature and filtered over a pad of celite *via* vacuum filtration. The filtrate was collected and concentrated, then purified by chromatography on silica gel.

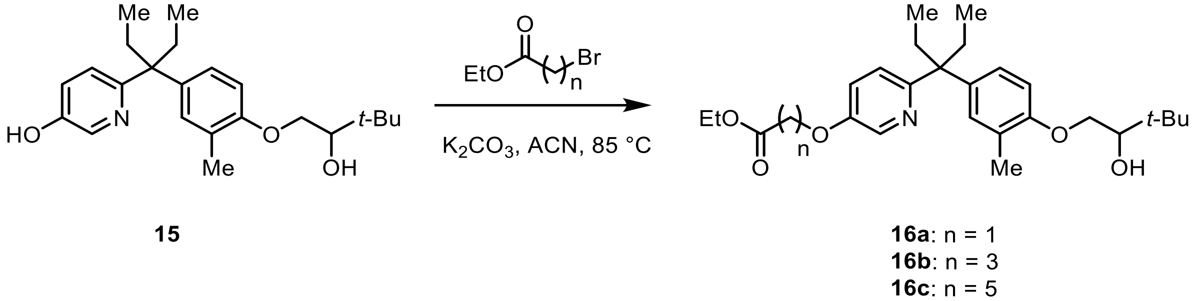

Ethyl 2-((6-(3-(4-(2-hydroxy-3,3-dimethylbutoxy)-3-methylphenyl)pentan-3-yl)pyridin-3-yl)oxy)acetate (16a): Synthesized following **General Procedure A** using ethyl bromoacetate (14 μL, 0.13 mmol, 1.3 equiv), hydroxy pyridine **15** (36 mg, 0.091 mmol, 1 equiv) and K_2CO_3 (27 mg, 0.19 mmol, 2.0 equiv) in ACN (0.5 mL). Purification by chromatography on silica gel using 20% EtOAc:hexanes as eluent provided ethyl ester **16a** as a clear oil (41 mg) in 93% yield. R_f 0.30 (20% EtOAc:hexanes); ^{1}H NMR (400 MHz, $CDCl_3$) δ 8.26 (s, 1H), δ 7.14–6.84 (m, 4H), δ 6.70 (d, *J* = 8.5 Hz, 1H), δ 4.63 (s, 2H), δ 4.27 (q, *J* = 7.1 Hz, 2H), δ 4.07 (dd, *J* = 9.1, 2.5 Hz, 1H), δ 3.84 (t, *J* = 8.9 Hz, 1H), δ 3.69 (dt, *J* = 8.5, 2.5 Hz, 1H), δ 2.42 (d, *J* = 2.6 Hz, 1H), δ 2.26–2.06 (m, 7H), δ 1.29 (t, *J* = 7.1 Hz, 3H), δ 0.99 (s, 9H), δ 0.60 (t, *J* = 7.2 Hz, 6H). HRMS (ESI) calculated for $C_{27}H_{40}NO_5$ $[M + H]^+$: 458.29010, found 458.28977.

Ethyl 4-((6-(3-(4-(2-hydroxy-3,3-dimethylbutoxy)-3-methylphenyl)pentan-3-yl)pyridin-3-yl)oxy)butanoate (16b): Synthesized following **General Procedure A** using ethyl 4-bromobutyrate (29 μL, 0.21 mmol, 1.3 equiv), hydroxy pyridine **15** (58 mg, 0.16 mmol, 1 equiv) and K_2CO_3 (43 mg, 0.31 mmol, 2.0 equiv) in ACN (0.8 mL). Purification by chromatography on silica gel using 25% EtOAc:hexanes as eluent provided ethyl ester **16b** as a yellow oil (62 mg) in 82% yield. R_f 0.62 (40% EtOAc:hexanes); ^{1}H NMR (400 MHz, $CDCl_3$) δ 8.23 (d, *J* = 2.8 Hz, 1H), δ 7.04 (dd, *J* = 8.6, 2.7 Hz, 1H), δ 6.99–6.90 (m, 2H), δ 6.88 (s, 1H), δ 6.69 (d, *J* = 8.4 Hz, 1H), δ 4.14 (q, *J* = 7.1 Hz, 2H), δ 4.07 (dd, *J* = 9.2, 2.7 Hz, 1H), δ 4.02 (t, *J* = 5.9 Hz, 2H), δ 3.83 (t, *J* = 8.7 Hz, 1H), δ 3.68 (dt, *J* = 8.9, 2.1 Hz, 1H), δ 2.51 (t, *J* = 7.2 Hz, 2H), δ 2.43 (d, *J* = 2.6 Hz, 1H), δ 2.26–2.05 (m, 9H), δ 1.25 (t, *J* = 7.1 Hz, 3H), δ 0.99 (s, 9H), δ 0.60 (t, *J* = 7.2 Hz, 6H). HRMS (ESI) calculated for $C_{29}H_{44}NO_5$ $[M + H]^+$: 486.32140, found 486.32240.

Ethyl 6-((6-(3-(4-(2-hydroxy-3,3-dimethylbutoxy)-3-methylphenyl)pentan-3-yl)pyridin-3-yl)oxy)hexanoate (16c): Synthesized following **General Procedure A** using ethyl 6-bromohexanoate (52 μL, 0.29 mmol, 1.3 equiv), hydroxy pyridine **15** (83 mg, 0.22 mmol, 1 equiv) and K_2CO_3 (62 mg, 0.45 mmol, 2.0 equiv) in ACN (1.1 mL). Purification by chromatography on silica gel using 20% EtOAc:hexanes as eluent provided **16c** as a clear oil (93 mg) in 81% yield. R_f 0.71 (40% EtOAc:hexanes); ^{1}H NMR (400 MHz, $CDCl_3$) δ 8.23 (d, *J* = 2.8 Hz, 1H), δ 7.03 (dd, *J* = 9.2, 3.6 Hz, 1H), δ 6.99–6.91 (m, 2H), δ 6.88 (s, 1H), δ 6.69 (d, *J* = 8.5 Hz, 1H), δ 4.12 (q, *J* = 7.1 Hz, 2H), δ 4.07 (dd, *J* = 9.1, 2.3 Hz, 1H), δ 3.96 (t, *J* = 6.3 Hz, 2H), δ 3.83 (t, *J* = 8.9 Hz, 1H), δ 3.68 (dt, *J* = 8.5, 2.5 Hz, 1H), δ 2.43 (d, *J* = 2.6 Hz, 1H), δ 2.32 (t, *J* = 7.4 Hz, 2H), δ 2.26–2.07 (m, 7H), δ 1.85–1.64 (m, 4H), δ 1.55–1.44 (m, 2H), δ 1.24 (t, *J* = 7.1 Hz, 3H), δ 0.99 (s, 9H), δ 0.60 (t, *J* = 7.2 Hz, 6H). ^{13}C NMR (125 MHz, $CDCl_3$) δ 173.59, 159.73, 154.49, 152.71, 140.31, 135.33, 130.34, 125.88, 125.73, 123.13, 121.43, 110.38, 77.2, 69.24, 67.92, 60.27, 50.98, 34.23,

33.57, 28.96, 28.72, 26.06. 25.61, 24.69, 16.63, 14.26, 8.37. HRMS (ESI) calculated for $C_{31}H_{48}NO_5$ $[M + H]^+$: 514.35270, found 514.35237.

General Procedure B: Formation of hydroxamic acids 17a–c and 27a–c

Hydroxylamine (50 wt% solution in H_2O, 500 equiv) followed by KOH (3.0 M solution in H_2O, 7.0 equiv) were added in that order to a vigorously stirring solution of ethyl esters **16a–c** or **26a–c** (1 equiv) at 0 °C in MeOH under air. The reaction mixture was left to warm slowly for 12–24 hours and was then neutralized to pH 7 and extracted with EtOAC (3x 10 mL). The organic fractions were dried over anhydrous Na_2SO_4, filtered, concentrated *in vacuo* and purified by reversed-phase chromatography on octadecyl-functionalized silica gel.

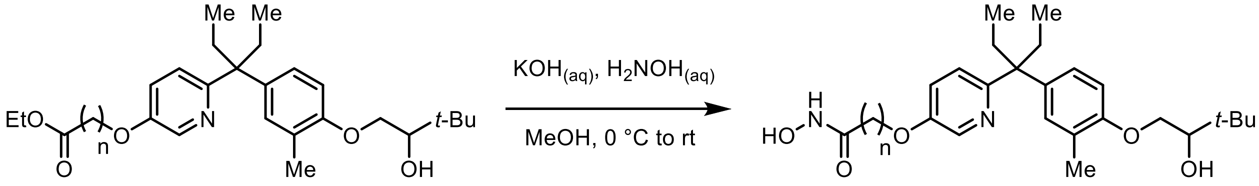

16a: n = 1
16b: n = 3
16c: n = 5

AM-155, **17a**: n = 1
AM-171, **17b**: n = 3
AM-172, **17c**: n = 5

***N*-hydroxy-2-((6-(3-(4-(2-hydroxy-3,3-dimethylbutoxy)-3-methylphenyl)pentan-3-yl)pyridin-3-yl)oxy)acetamide (17a):** Synthesized following **General Procedure B** using hydroxylamine (50 wt% solution in H_2O, 3.1 mL, 45 mmol, 500 equiv), KOH (3.0 M solution in H_2O, 0.21 mL, 0.62 mmol, 7.0 equiv) and ethyl ester **16a** (41 mg, 0.10 mmol, 1 equiv) in MeOH (0.9 mL). Purification by reversed-phase chromatography on octadecyl-functionalized silica gel using a gradient of 65–80% MeOH:H_2O over a period of 15 minutes as eluent provided AM-155 (**17a**) as a fine white powder (19 mg) in 48% yield after lyophilization. ^1H NMR (500 MHz, d_6-DMSO) δ 10.84 (s, 1H), δ 8.98 (s, 1H), δ 8.21 (d, J = 2.9 Hz, 1H), δ 7.24 (dd, J = 8.8, 3.0 Hz, 1H), δ 7.04 (d, J = 8.8 Hz, 1H), δ 6.90 (dd, J = 8.5, 2.1 Hz, 1H), δ 6.85 (s, 1H), δ 6.80 (d, J = 8.5 Hz, 1H), δ 4.77 (d, J = 5.3 Hz, 1H), δ 4.51 (s, 2H), δ 4.01 (dd, J = 10.1, 3.2 Hz, 1H), δ 3.75 (dd, J = 10.0, 7.2 Hz, 1H), δ 3.47–3.42 (m, 1H), δ 2.22–2.02 (m, 7H), δ 0.92 (s, 9H), δ 0.54 (t, J = 7.3 Hz, 6H). ^{13}C NMR (100 MHz, $CDCl_3$) δ 165.03, 161.78, 154.65, 150.97, 139.71, 135.52, 130.27, 125.91, 125.85, 123.44, 121.83, 110.46, 77.2, 69.34, 66.65, 51.15, 33.60, 28.59, 26.07, 16.62, 8.33. HRMS (ESI) calculated for $C_{25}H_{36}N_2O_5Na$ $[M + Na]^+$: 467.2516, found 467.2506. Purity >95% by LC-MS, t_r = 9.68 minutes.

***N*-hydroxy-4-((6-(3-(4-(2-hydroxy-3,3-dimethylbutoxy)-3-methylphenyl)pentan-3-yl)pyridin-3-yl)oxy)butanamide (17b):** Synthesized following **General Procedure B** using hydroxylamine (50 wt% solution in H_2O, 4.2 mL, 61 mmol, 500 equiv), KOH (3.0 M solution in H_2O, 0.31 mL, 0.85 mmol, 7.0 equiv) and ethyl ester **16b** (59 mg, 0.12 mmol, 1 equiv) in MeOH (1.2 mL). Purification by reversed-phase chromatography on octadecyl-functionalized silica gel using a gradient of 65–80% MeOH:H_2O over a period of 15 minutes as eluent provided AM-171 (**17b**) as a fine white powder (38 mg) in 66% yield after lyophilization. ^1H NMR (400 MHz, d_6-DMSO) δ 10.40 (s, 1H), δ 8.69 (s, 1H), δ 8.15 (d, *J* = 2.8 Hz, 1H), δ 7.20 (dd, *J* = 9.1, 2.9 Hz, 1H), δ 6.98 (d, *J* = 8.8 Hz, 1H), δ 6.87 (dd, *J* = 8.7, 2.2 Hz, 1H), δ 6.78 (s, 1H), δ 6.77 (d, *J* = 8.5 Hz, 1H), δ 4.77 (d, *J* = 5.2 Hz, 1H), δ 4.03–3.93 (m, 3H), δ 3.71 (dd, *J* = 10.3, 7.2 Hz, 1H), δ 3.45–3.38 (m, 1H), δ 2.19–2.00 (m, 9H), δ 1.90 (quintet, *J* = 6.5 Hz, 2H), δ 0.89 (s, 9H), δ 0.51 (t, *J* = 7.2 Hz, 6H). ^{13}C NMR (125 MHz, CD_3OD) δ 170.85, 159.48, 155.11, 153.17, 139.17, 135.11, 128.80, 125.69, 125.62, 123.45, 121.50, 110.10, 77.22, 69.55, 67.08, 50.72, 33.68, 28.81, 28.16, 25.18, 24.96, 15.37, 7.27. HRMS (ESI) calculated for $C_{27}H_{40}N_2O_5Na$ $[M + Na]^+$: 495.2829, found 495.2825. Purity >95% by LC-MS, t_r = 9.04 minutes.

***N*-hydroxy-6-((6-(3-(4-(2-hydroxy-3,3-dimethylbutoxy)-3-methylphenyl)pentan-3-yl)pyridin-3-yl)oxy)hexanamide (17c):** Synthesized following **General Procedure B** using hydroxylamine (50 wt% solution in H_2O, 5.8 mL, 84 mmol, 500 equiv), KOH (3.0 M solution in H_2O, 0.41 mL, 1.2 mmol, 7.0 equiv) and ethyl ester **16c** (86 mg, 0.17 mmol, 1 equiv) in MeOH (0.9 mL). Purification by reversed-phase chromatography on octadecyl-functionalized silica gel using a gradient of 65–80% MeOH:H_2O over a period of 15 minutes as eluent provided AM-172 (**17c**) as a fine white powder (29 mg) in 34% yield after lyophilization. ^1H NMR (400 MHz, d_6-DMSO) δ 10.32 (s, 1H), δ 8.66 (s, 1H), δ 8.15 (d, *J* = 2.9 Hz, 1H), δ 7.21 (dd, *J* = 8.7, 2.7 Hz, 1H), δ 6.98 (d, *J* = 8.8 Hz, 1H), δ 6.90–6.73 (m, 3H), δ 4.77 (d, *J* = 5.1 Hz, 1H), δ 4.02–3.91 (m, 3H), δ 3.71 (dd, *J* = 10.0, 7.3 Hz, 1H), δ 3.45–3.39 (m, 1H), δ 2.18–2.00 (m, 7H), δ 1.94 (t, *J* = 7.3 Hz, 2H), δ 1.72–1.62 (m, 2H), δ 1.57–1.46 (m, 2H), δ 1.40–1.30 (m, 2H), δ 0.89 (s, 9H), δ 0.51 (t, *J* = 7.2 Hz, 6H). ^{13}C NMR (125 MHz, CD_3OD) δ 169.48, 159.19, 155.05, 152.89, 139.48, 135.74, 129.87, 126.00, 125.32, 123.09, 121.66, 110.86, 76.27, 70.31, 68.09, 50.79, 34.41, 32.64, 28.89, 28.34, 26.55, 25.57, 25.32, 16.94, 8.80. HRMS (ESI) calculated for $C_{29}H_{44}N_2O_5Na$ $[M + Na]^+$: 523.3142, found 523.3138. Purity >95% by LC-MS, t_r = 9.64 minutes.

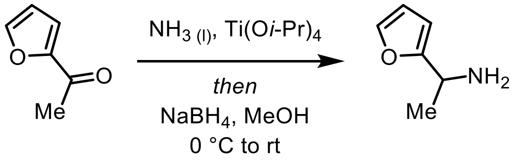

1-(furan-2-yl)ethan-1-amine: Titanium (IV) isopropoxide (54 mL, 0.18 mol, 2.0 equiv) was added drop-wise to a neat solution of 2-acetylfuran (9.1 mL, 91 mmol, 1 equiv) at room temperature and left to stir for five minutes, at which point an orange slurry is formed. After the five minutes had passed, ammonia (7 M solution in MeOH, 65 mL, 0.45 mol, 5.0 equiv) was added dropwise over several minutes and the now cloudy reaction mixture was allowed to stir overnight. After stirring overnight, the reaction mixture was cooled to 0 °C and $NaBH_4$ (5.1 g, 0.14 mol, 1.5 equiv) was added in portions. After complete addition of the reducing agent, the reaction mixture was allowed to stir for three hours at room temperature before being cooled back to 0 °C and quenched with 100-mL of an aqueous NH_4OH solution, which formed a white-beige precipitate. The precipitate was filtered over a pad of celite and the dark orange filtrate was extracted using Et_2O (3x 200 mL). The organic fractions were combined and acidified to pH 1-3 with 6 M HCl and extracted again with three 200-mL volumes of a brine solution. The acidic, aqueous fractions were combined and basified to pH > 12 with 6 M NaOH and extracted again with Et_2O (3x 200 mL). The organic fractions were combined and dried over anhydrous Na_2SO_4, filtered, and concentrated to afford 6.4 g (64%) of 1-(furan-2-yl)ethan-1-amine as a yellow/orange tinted liquid which was sufficiently pure for use without further purification (caution: the product is volatile, concentrate at no higher than 30 °C and no lower than 200 mbar). The spectroscopic data is in agreement with that published in the literature[3].

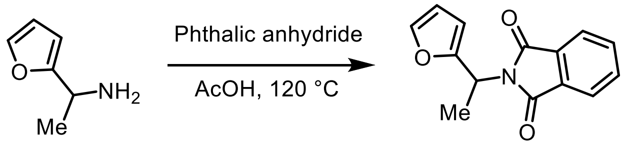

20

2-(1-(furan-2-yl)ethyl)isoindoline-1,3-dione (20): Phthalic anhydride (9.7 g, 66 mmol, 1.2 equiv) was added in one portion to a stirring solution of 1-(furan-2-yl)ethan-1-amine (6.1 g, 55 mmol, 1 equiv) in neat AcOH (55 mL) under air. The reaction mixture was brought to reflux and stirred for two hours before being neutralized to pH 7 using solid $NaHCO_3$ in portions (caution: gas evolution). The now black reaction mixture was extracted with EtOAc (3x 100 mL) followed by a 50-mL wash with a brine solution. The organic fractions were dried over anhydrous $MgSO_4$, filtered, and concentrated to afford a black-brown solid. The residue was purified by

chromatography on silica gel using 15% EtOAc:hexanes as eluent to provide phthalimide **20** as a white solid (7.1 g) in a 54% yield. R_f 0.45 (25% EtOAc:hexanes); ^1H NMR (500 MHz, $CDCl_3$) δ 7.85–7.81 (m, 2H), δ 7.73–7.69 (m, 2H), δ 7.31 (d, *J* = 1.0 Hz, 1H), δ 6.38–6.33 (m, 2H), δ 5.58 (q, *J* = 7.2 Hz, 1H), δ 1.86 (d, *J* = 7.2 Hz, 3H). ^{13}C NMR (125 MHz, $CDCl_3$) δ 167.69, 152.94, 141.84, 133.96, 131.98, 123.29, 110.32, 107.06, 43.41, 16.46. HRMS (ESI) calculated for $C_{14}H_{12}NO_3$ $[M + H]^+$: 242.08117, found 242.08118.

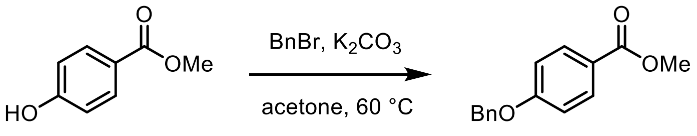

18

Methyl 4-(benzyloxy)benzoate (18): Benzyl bromide (1.4 mL, 21 mmol, 1.1 equiv), freshly filtered through a plug of basic alumina, was added in one portion to a vigorously stirred slurry of methyl 4-hydroxybenzoate (2.9 g, 19 mmol, 1 equiv) and solid K_2CO_3 (4.2 g, 30 mmol, 1.6 equiv) in acetone (75 mL) under air. The reaction mixture was heated at reflux overnight, at which point TLC analysis indicated complete consumption of the starting material. The reaction mixture was cooled to room temperature and then 100 mL cold H_2O was added resulting in the formation of a white precipitate. The precipitate was filtered, rinsed with three 20-mL volumes of H_2O and dried under high vacuum to afford 3.6 g (98%) of benzyl ether **18** as a white solid which was sufficiently pure for use without further purification. The spectroscopic data is in agreement with that published in the literature[4].

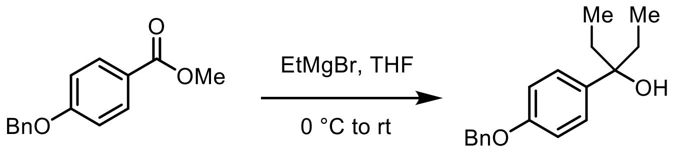

18 **19**

3-(4-(benzyloxy)phenyl)pentan-3-ol (19): EtMgBr (3.0 M solution in Et_2O, 12 mL, 37 mmol, 2.5 equiv) was added drop-wise over several minutes to a 0 °C solution of benzyl ether **18** (3.6 g, 15 mmol, 1 equiv) in anhydrous THF (60 mL). At this point, the solution has become a yellow color and the reaction mixture was brought to room temperature. After four hours, the reaction mixture was cooled to 0 °C and quenched with 25-mL of a saturated solution of NH_4Cl. Excess THF was evaporated *in vacuo*, and the remaining clear residue was extracted with three 20-mL volumes of EtOAc, followed by a 10-mL wash with brine solution. The organic fractions were dried over anhydrous $MgSO_4$, filtered, and concentrated to afford 3.8 g (95%) of tertiary alcohol **19** as a white

solid which was sufficiently pure for use without further purification. The spectroscopic data is in agreement with that published in the literature[5].

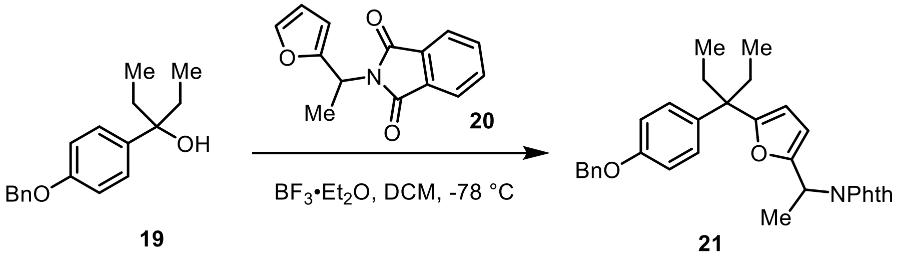

19 21

2-(1-(5-(3-(4-(benzyloxy)phenyl)pentan-3-yl)furan-2-yl)ethyl)isoindoline-1,3-dione (21): $BF_3{\cdot}Et_2O$ (2.1 mL, 17 mmol, 1.1 equiv) was added drop-wise over several minutes to a –78 °C solution of tertiary alcohol **19** (4.1 g, 15 mmol, 1 equiv) and phthalimide **20** (4.8 g, 20 mmol, 1.3 equiv) in anhydrous DCM (76 mL). After two hours, the dark purple reaction mixture was quenched with 150-mL of a saturated solution of $NaHCO_3$ and left to warm to room temperature and stir for an additional 10–15 min. The now orange solution was extracted with DCM (3x 200 mL), followed by a 50-mL wash with brine solution. The organic fractions were dried over anhydrous $MgSO_4$, filtered, and concentrated. The residue was purified by recrystallization from hot EtOH to provide phthalimide **21** as fluffy, beige-white crystals (6.5 g) in 86% yield. R_f 0.67 (100% DCM); ^{1}H NMR (500 MHz, $CDCl_3$) δ 7.80–7.76 (m, 2H), δ 7.69–7.64 (m, 2H), δ 7.44–7.31 (m, 5H), δ 6.99 (d, *J* = 8.8 Hz, 2H), δ 6.75 (d, *J* = 8.8 Hz, 2H), δ 6.25 (dd, *J* = 3.5, 1.0 Hz, 1H), δ 6.09 (d, *J* = 3.2 Hz, 1H), δ 5.47 (q, *J* = 7.0 Hz, 1H), δ 4.95 (s, 2H), δ 2.00–1.85 (m, 4H), δ 1.77 (d, *J* = 7.1 Hz, 3H), δ 0.64 (t, *J* = 7.3 Hz, 3H), δ 0.60 (t, *J* = 7.3 Hz, 3H). ^{13}C NMR (125 MHz, $CDCl_3$) δ 167.64, 160.07, 156.72, 151.06, 137.69, 137.26, 133.78, 131.97, 128.55, 128.25, 127.89, 127.50, 123.10, 113.81, 107.21, 106.75, 69.88, 47.34, 43.77, 28.90, 28.77, 16.41, 8.45, 8.28. HRMS (ESI) calculated for $C_{32}H_{32}NO_4$ $[M + H]^+$: 494.23258, found 494.23233.

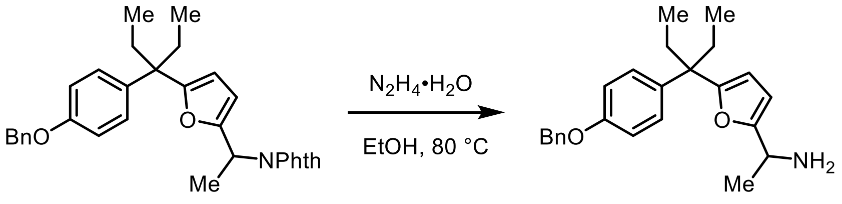

21 22

1-(5-(3-(4-(benzyloxy)phenyl)pentan-3-yl)furan-2-yl)ethan-1-amine (22): Hydrazine monohydrate (9.4 mL, 0.19 mol, 15 equiv) was added in one portion to a stirring solution of phthalimide **21** (6.4 g, 13 mmol, 1 equiv) in EtOH (65 mL) and brought to reflux for one hour, at

which point a white precipitate began to crash out of solution, signaling the end of the reaction. The reaction mixture was allowed to cool to room temperature, and the white precipitate was dissolved using 50-mL of a 1 M NaOH solution. The reaction mixture was extracted with EtOAc (3x 100 mL) and the organic fractions were dried over anhydrous Na_2SO_4, filtered, and concentrated to afford 4.1 g (88%) of primary amine **22** as a yellow, viscous oil which was sufficiently pure for use without further purification. R_f 0.12 (100% DCM); ^{1}H NMR (500 MHz, $CDCl_3$) δ 7.45–7.30 (m, 5H), δ 7.09 (d, *J* = 8.8 Hz, 2H), δ 6.89 (d, *J* = 8.8 Hz, 2H), δ 6.07 (d, *J* = 3.0 Hz, 1H), δ 6.01 (dd, *J* = 3.0, 0.7 Hz, 1H), δ 5.04 (s, 2H), δ 3.96 (q, *J* = 6.6 Hz, 1H), δ 2.09–1.93 (m, 4H), δ 1.42 (bs, 2H), δ 1.34 (d, *J* = 6.6 Hz, 3H), δ 0.69 (t, *J* = 7.3 Hz, 6H). ^{13}C NMR (125 MHz, $CDCl_3$) δ 159.45, 158.97, 156.87, 137.98, 137.22, 128.55, 128.31, 127.91, 127.56, 114.03, 106.88, 102.88, 69.98, 47.37, 45.01, 28.82, 28.79, 21.88, 8.45. HRMS (ESI) calculated for $C_{24}H_{29}NO_2Na$ [M + Na]$^+$: 386.20905, found 386.20794. Note: Molecular ion (M^+) peak is very small due to fragmentation, producing a major peak with m/z = 347.19986 (loss of NH_2).

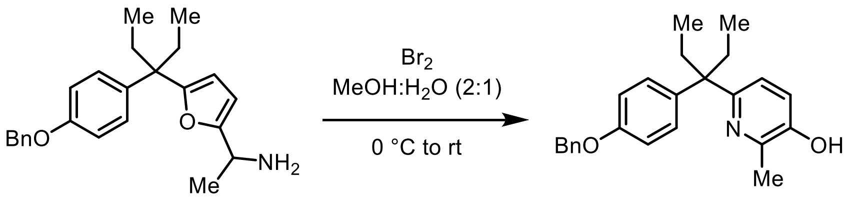

22

6-(3-(4-(benzyloxy)phenyl)pentan-3-yl)-2-methylpyridin-3-ol: Bromine (0.71 mL, 14 mmol, 1.2 equiv) was added all in one portion to a vigorously stirring solution of primary amine **22** (4.1 g, 11 mmol, 1 equiv) at 0 °C in a 2:1 mixture of MeOH:H_2O (114 mL) under air. The orange reaction mixture was subsequently warmed to room temperature and stirred for a minimum of 24 hours, at which point the reaction mixture was quenched with 50-mL of a saturated $Na_2S_2O_3$ solution and extracted with EtOAc (3x 100 mL). The organic fractions were dried over anhydrous Na_2SO_4, filtered, and concentrated to afford an orange-brown foam. The residue was purified by chromatography on silica gel using 25% EtOAc:hexanes as eluent to provide hydroxy pyridine as a yellow solid (2.1 g) in 51% yield. R_f 0.38 (25% EtOAc:hexanes); ^{1}H NMR (500 MHz, $CDCl_3$) δ 7.49–7.30 (m, 5H), δ 7.09 (d, *J* = 8.8 Hz, 2H), δ 6.90–6.85 (m, 3H), δ 6.77–6.72 (m, 1H), δ 5.02 (s, 2H), δ 4.66 (bs, 1H), δ 2.48 (s, 3H), δ 2.27–2.07 (m, 4H), δ 7.14 (t, *J* = 7.1 Hz, 6H). HRMS (ESI) calculated for $C_{24}H_{28}NO_2$ [M + H]$^+$: 362.2115, found 362.2113.

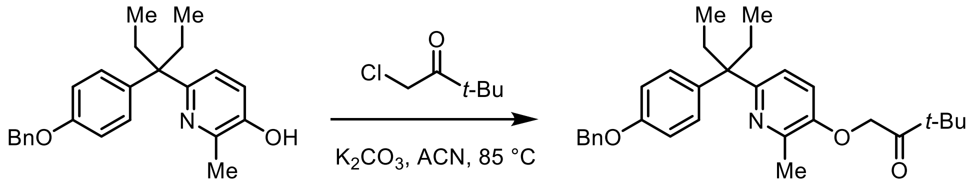

23

1-((6-(3-(4-(benzyloxy)phenyl)pentan-3-yl)-2-methylpyridin-3-yl)oxy)-3,3-dimethylbutan-2-one (23): 1-chloropinacolone (1.1 mL, 7.5 mmol, 1.3 equiv), freshly filtered through a plug of basic alumina, was added in one portion to a vigorously stirred slurry of hydroxy pyridine (2.1 g, 5.8 mmol, 1 equiv) and solid K_2CO_3 (1.6 g, 12 mmol, 2.0 equiv) in ACN (29 mL) under air. The reaction mixture was heated at reflux overnight, at which point TLC analysis indicated complete consumption of the starting material. The reaction mixture was allowed to cool to room temperature and filtered over a pad of celite *via* vacuum filtration. The filtrate was collected as a yellow solution and concentrated to a dark-yellow oil. The residue was purified by chromatography on silica gel using 15% EtOAc:hexanes as eluent to provide ketone **23** as a yellow oil (2.2 g) in 83% yield. R_f 0.63 (25% EtOAc:hexanes); ^1H NMR (500 MHz, $CDCl_3$) δ 7.50–7.29 (m, 5H), δ 7.10 (d, *J* = 8.8 Hz, 2H), δ 6.87 (d, *J* = 8.8 Hz, 2H), δ 6.77–6.69 (m, 2H), δ 5.02 (s, 2H), δ 4.83 (s, 2H), δ 2.52 (s, 3H), δ 2.28–2.08 (m, 4H), δ 1.25 (s, 9H), δ 0.62 (t, *J* = 7.4 Hz, 6H). HRMS (ESI) calculated for $C_{30}H_{38}NO_3$ $[M + H]^+$: 460.28462, found 460.28563.

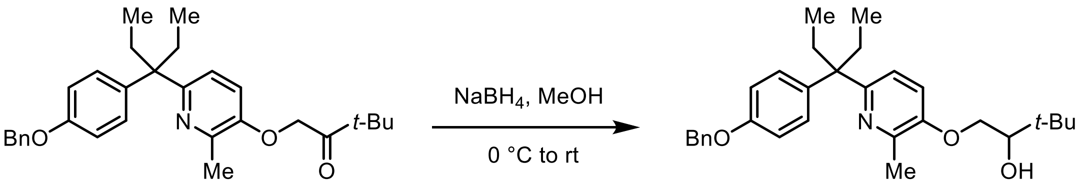

23 **24**

1-((6-(3-(4-(benzyloxy)phenyl)pentan-3-yl)-2-methylpyridin-3-yl)oxy)-3,3-dimethylbutan-2-ol (24): $NaBH_4$ (0.37 g, 9.6 mmol, 2.0 equiv) was added in 100 mg portions to a 0 °C solution of ketone **23** (2.2 g, 4.8 mmol, 1 equiv) in MeOH (48 mL) under air. The reaction mixture was taken out of the ice bath and warmed to room temperature and stirred for one to two hours. The reaction mixture was subsequently cooled to 0 °C and quenched (slowly) with 50 mL of H_2O or until all gas evolution had ceased. The reaction mixture was concentrated to remove most of the MeOH and was extracted with EtOAc (3x 100 mL), followed by a 50-mL wash with brine solution. The organic fractions were dried over anhydrous Na_2SO_4, filtered, and concentrated to afford a clear oil. The residue was purified by chromatography on silica gel using 10–15% EtOAc:hexanes as eluent to

provide secondary alcohol **24** as a clear, viscous oil (1.8 g) in 82% yield. R_f 0.57 (10% EtOAc:hexanes); ^{1}H NMR (500 MHz, $CDCl_3$) δ 7.49–7.30 (m, 5H), δ 7.10 (d, *J* = 8.8 Hz, 2H), δ 6.94 (d, *J* = 8.5 Hz, 1H), δ 6.87 (d, *J* = 8.8 Hz, 2H), δ 6.80 (d, *J* = 8.5 Hz, 1H), δ 5.03 (s, 2H), δ 4.06 (dd, *J* = 9.1, 2.6 Hz, 1H), δ 3.84 (t, *J* = 8.4 Hz, 1H), δ 3.71 (dt, *J* = 9.2, 2.8 Hz, 1H), δ 2.47 (s, 3H), δ 2.36 (d, *J* = 3.1 Hz, 1H), δ 2.28–2.09 (m, 4H), δ 1.01 (s, 9H), δ 0.62 (t, *J* = 7.3 Hz, 6H). ^{13}C NMR (125 MHz, $CDCl_3$) δ 158.39, 156.54, 150.26, 146.34, 140.66, 137.29, 128.83, 128.55, 127.89, 127.57, 120.74, 117.68, 113.86, 77.24, 69.96, 69.56, 53.43, 50.98, 33.65, 28.92, 26.06, 19.82, 8.47. HRMS (ESI) calculated for $C_{30}H_{40}NO_3$ $[M + H]^+$: 462.3003, found 462.2997.

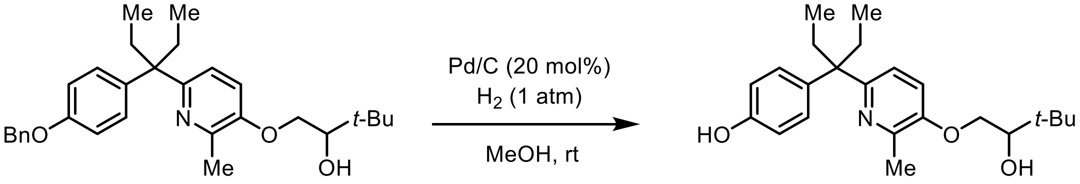

24 **25**

4-(3-(5-(2-hydroxy-3,3-dimethylbutoxy)-6-methylpyridin-2-yl)pentan-3-yl)phenol (25): Pd/C (84 mg, 0.79 mmol, 0.20 equiv) was added in one portion to a solution of secondary alcohol **24** (1.8 g, 3.9 mmol, 1 equiv) in anhydrous MeOH (40 mL) at room temperature. An atmosphere of hydrogen was established using a balloon and a vent to relieve the round-bottom flask of all argon. The balloon was refilled with hydrogen and the needle inserted through the rubber septum. The resulting black suspension was stirred vigorously overnight and filtered over a pad of celite once complete by TLC analysis. The filtrate was collected and concentrated under reduced pressure to afford a pale-yellow oil. The residue was purified by chromatography on silica gel using 20% EtOAc:hexanes as eluent to provide phenol **25** as a white powder (0.88 g) in 60% yield. R_f 0.45 (30% EtOAc:hexanes); ^{1}H NMR (500 MHz, $CDCl_3$) δ 7.08–6.97 (m, 3H), δ 6.84 (d, *J* = 8.6 Hz, 2H), δ 6.39 (d, *J* = 8.5 Hz, 2H), δ 4.10 (dd, *J* = 9.1, 2.4 Hz, 1H), δ 3.88 (t, *J* = 8.8 Hz, 1H), δ 3.73 (dt, *J* = 8.6, 2.9 Hz, 1H), δ 2.48 (s, 3H), δ 2.36 (d, *J* = 3.0 Hz, 1H), δ 2.30–2.22 (m, 2H), δ 2.09–2.00 (m, 2H), δ 1.03 (s, 9H), δ 0.58 (t, *J* = 7.2 Hz, 6H). HRMS (ESI) calculated for $C_{23}H_{34}NO_3$ $[M + H]^+$: 372.25332, found 372.25481.

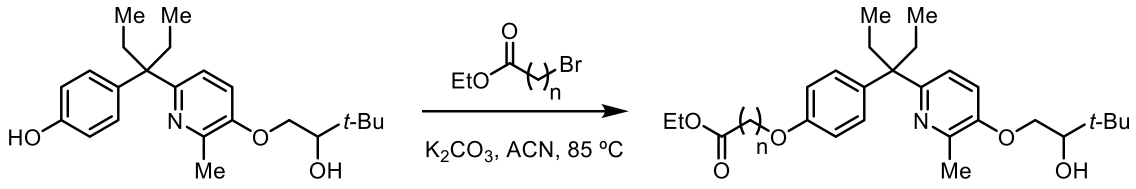

25

26a: n = 1
26b: n = 3
26c: n = 5

Ethyl 2-(4-(3-(5-(2-hydroxy-3,3-dimethylbutoxy)-6-methylpyridin-2-yl)pentan-3-yl)phenoxy)acetate (26a): Synthesized following **General Procedure A** using ethyl bromoacetate (12 μL, 0.11 mmol, 1.3 equiv), phenol **25** (30 mg, 0.081 mmol, 1 equiv) and K_2CO_3 (22 mg, 0.16 mmol, 2.0 equiv) in ACN (0.4 mL). Purification by chromatography on silica gel using 20% EtOAc:hexanes as eluent provided ethyl ester **26a** as a clear oil (23 mg) in 62% yield. R_f 0.57 (30% EtOAc:hexanes); ^1H NMR (400 MHz, $CDCl_3$) δ 7.08 (d, *J* = 8.7 Hz, 2H), δ 6.92 (d, *J* = 8.5 Hz, 1H), δ 6.81–6.75 (m, 3H), δ 4.57 (s, 2H), δ 4.26 (q, *J* = 7.1 Hz, 2H), δ 4.05 (dd, *J* = 9.1, 2.4 Hz, 1H), δ 3.82 (t, *J* = 8.9 Hz, 1H), δ 3.73–3.67 (m, 1H), δ 2.44 (s, 3H), δ 2.36 (d, *J* = 2.6 Hz, 1H), δ 2.27–2.05 (m, 4H), δ 1.28 (t, *J* = 7.1 Hz, 3H), δ 1.00 (s, 9H), δ 0.59 (t, *J* = 7.3 Hz, 6H). ^{13}C NMR (125 MHz, $CDCl_3$) δ 169.16, 158.21, 155.55, 150.27, 146.58, 141.51, 128.89, 120.72, 117.64, 113.79, 77.22, 69.55, 65.55, 61.27, 51.00, 33.64, 28.90, 26.04, 19.80, 14.17, 8.43. HRMS (ESI) calculated for $C_{27}H_{40}NO_5$ $[M + H]^+$: 458.29010, found 458.29047.

Ethyl 4-(4-(3-(5-(2-hydroxy-3,3-dimethylbutoxy)-6-methylpyridin-2-yl)pentan-3-yl)phenoxy)butanoate (26b): Synthesized following **General Procedure A** using ethyl 4-bromobutyrate (15 μL, 0.11 mmol, 1.3 equiv), phenol **25** (30 mg, 0.081 mmol, 1 equiv) and K_2CO_3 (22 mg, 0.16 mmol, 2.0 equiv) in ACN (0.4 mL). Purification by chromatography on silica gel using 20% EtOAc:hexanes as eluent provided ethyl ester **26b** as a clear oil (33 mg) in 84% yield. R_f 0.72 (30% EtOAc:hexanes); ^1H NMR (400 MHz, $CDCl_3$) δ 7.06 (d, *J* = 8.7 Hz, 2H), δ 6.92 (d, *J* = 8.5 Hz, 1H), δ 6.80–6.73 (m, 3H), δ 4.13 (q, *J* = 7.1 Hz, 2H), δ 4.05 (dd, *J* = 9.1, 2.3 Hz, 1H), δ 3.96 (t, *J* = 6.0 Hz, 2H), δ 3.82 (t, *J* = 8.8 Hz, 1H), δ 3.70 (dt, *J* = 8.7, 2.4 Hz, 1H), δ 2.49 (t, *J* = 7.3 Hz, 2H), δ 2.45 (s, 3H), δ 2.36 (d, *J* = 2.7 Hz, 1H), δ 2.27–2.04 (m, 4H), δ 1.24 (t, *J* = 7.1 Hz, 3H), δ 1.00 (s, 9H), δ 0.60 (t, *J* = 7.3 Hz, 6H). ^{13}C NMR (125 MHz, $CDCl_3$) δ 173.30, 158.43, 156.47, 150.23, 146.30, 140.42, 128.78, 120.73, 117.67, 113.51, 77.22, 69.55, 66.54, 60.40, 50.94, 33.64, 30.88, 28.91, 26.05, 24.75, 19.81, 14.23, 8.45. HRMS (ESI) calculated for $C_{29}H_{44}NO_5$ $[M + H]^+$: 486.32140, found 486.32282.

Ethyl 6-(4-(3-(5-(2-hydroxy-3,3-dimethylbutoxy)-6-methylpyridin-2-yl)pentan-3-yl)phenoxy)hexanoate (26c): Synthesized following **General Procedure A** using ethyl 6-

bromohexanoate (0.2 mL, 1.2 mmol, 1.3 equiv), phenol **25** (0.35 g, 0.95 mmol, 1 equiv) and K_2CO_3 (0.26 g, 1.9 mmol, 2.0 equiv) in ACN (4.8 mL). Purification by chromatography on silica gel using 15% EtOAc:hexanes as eluent provided ethyl ester **26c** as a clear oil (0.45 g) in 93% yield. R_f 0.47 (20% EtOAc:hexanes); ^{1}H NMR (500 MHz, $CDCl_3$) δ 7.07 (d, *J* = 8.8 Hz, 2H), δ 6.93 (d, *J* = 8.5 Hz, 1H), δ 6.80 (m, 3H), δ 4.13 (q, *J* = 7.1 Hz, 2H), δ 4.06 (dd, *J* = 9.1, 2.6 Hz, 1H), δ 3.92 (t, *J* = 6.4 Hz, 2H), δ 3.83 (t, *J* = 8.8 Hz, 1H), δ 3.71 (dt, *J* = 8.5, 2.8 Hz, 1H), δ 2.46 (s, 3H), δ 2.36 (d, *J* = 3.1 Hz, 1H), δ 2.33 (t, *J* = 7.5 Hz, 2H), δ 2.26–2.09 (m, 4H), δ 1.82–1.66 (m, 4H), δ 1.53–1.46 (m, 2H), δ 1.26 (t, *J* = 7.1 Hz, 3H), δ 1.01 (s, 9H), δ 0.62 (t, *J* = 7.3 Hz, 6H). ^{13}C NMR (125 MHz, $CDCl_3$) δ 173.68, 158.49, 156.67, 150.22, 146.27, 140.21, 128.76, 120.77, 117.56, 113.50, 77.27, 69.55, 67.44, 60.24, 50.94, 34.29, 33.64, 29.06, 28.93. 26.04, 25.72, 24.72, 19.81, 14.26, 8.45. HRMS (ESI) calculated for $C_{31}H_{48}NO_5$ [M + H]$^+$: 514.35270, found 514.35358.

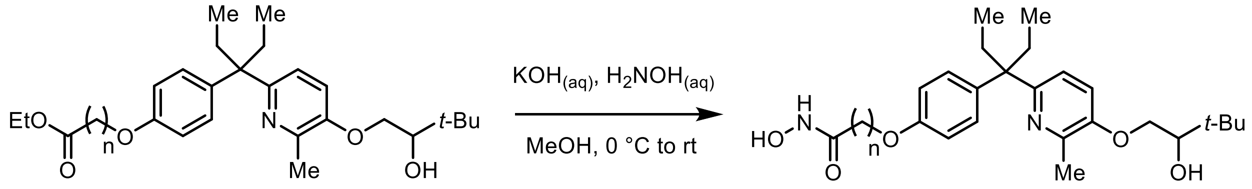

26a: n = 1
26b: n = 3
26c: n = 5

AM-191, **27a**: n = 1
AM-192, **27b**: n = 3
AM-193, **27c**: n = 5

***N*-hydroxy-2-(4-(3-(5-(2-hydroxy-3,3-dimethylbutoxy)-6-methylpyridin-2-yl)pentan-3-yl)phenoxy)acetamide (27a):** Synthesized following **General Procedure B** using hydroxylamine (50 wt% solution in H_2O, 1.7 mL, 25 mmol, 500 equiv), KOH (3.0 M solution in H_2O, 0.12 mL, 0.35 mmol, 7.0 equiv) and ethyl ester **26a** (23 mg, 0.051 mmol, 1 equiv) in MeOH (0.5 mL). Purification by reversed-phase chromatography on octadecyl-functionalized silica gel using a gradient of 65–80% MeOH:H_2O over a period of 15 minutes as eluent provided AM-191 (**27a**) as a fine white powder (12 mg) in 52% yield after lyophilization. ^{1}H NMR (400 MHz, *d*$_6$-DMSO) δ 10.78 (s, 1H), δ 8.93 (s, 1H), δ 7.19 (d, *J* = 8.6 Hz, 1H), δ 7.00 (d, *J* = 8.6 Hz, 2H), δ 6.88 (d, *J* = 8.5 Hz, 1H), δ 6.79 (d, *J* = 8.7 Hz, 2H), δ 4.86 (d, *J* = 5.1 Hz, 1H), δ 4.38 (s, 2H), δ 4.03 (dd, *J* = 10.3, 2.7 Hz, 1H), δ 3.73 (dd, *J* = 10.4, 7.6 Hz, 1H), δ 3.47–3.39 (m, 1H), δ 2.31 (s, 3H), δ 2.21–1.97 (m, 4H), δ 0.89 (s, 9H), δ 0.52 (t, *J* = 7.1 Hz, 6H). ^{13}C NMR (201 MHz, $CDCl_3$) δ 165.37, 157.68, 154.70, 150.51, 147.22, 142.16, 129.22, 120.47, 117.60, 113.78, 77.20, 69.58, 66.57, 50.98, 33.67, 28.71, 26.06, 19.40, 8.41. HRMS (ESI) calculated for $C_{25}H_{37}N_2O_5$ [M + H]$^+$: 445.26970, found 445.26956. Purity >95% by LC-MS, t_r = 7.49 minutes.

***N*-hydroxy-4-(4-(3-(5-(2-hydroxy-3,3-dimethylbutoxy)-6-methylpyridin-2-yl)pentan-3-yl)phenoxy)butanamide (27b):** Synthesized following **General Procedure B** using hydroxylamine (50 wt% solution in H_2O, 2.3 mL, 34 mmol, 500 equiv), KOH (3.0 M solution in H_2O, 0.16 mL, 0.48 mmol, 7.0 equiv) and ethyl ester **26b** (33 mg, 0.071 mmol, 1 equiv) in MeOH (0.7 mL). Purification by reversed-phase chromatography on octadecyl-functionalized silica gel using a gradient of 65–80% MeOH:H_2O over a period of 15 minutes as eluent to provide AM-192 (**27b**) as a fine white powder (20 mg) in 63% yield after lyophilization. ^{1}H NMR (400 MHz, CD_3OD) δ 7.19 (d, *J* = 8.6 Hz, 1H), δ 7.06–6.99 (m, 3H), δ 6.83–6.77 (m, 2H), δ 4.17 (dd, *J* = 10.0, 2.8 Hz, 1H), δ 3.98 (t, *J* = 6.1 Hz, 2H), δ 3.90 (dd, *J* = 10.0, 7.9 Hz, 1H), δ 3.65 (dd, *J* = 7.9, 2.8 Hz, 1H), δ 2.43 (s, 3H), δ 2.32–2.18 (m, 4H), δ 2.18–2.02 (m, 4H), δ 1.02 (s, 9H), δ 0.61 (t, *J* = 7.3 Hz, 6H). ^{13}C NMR (100 MHz, CD_3OD) δ 171.03, 157.34, 156.67, 151.04, 146.96, 140.04, 128.53, 120.58, 117.48, 113.31, 77.10, 69.75, 66.47, 50.62, 33.69, 28.97, 28.28, 25.14 (x 2C), 17.69, 7.37. HRMS (ESI) calculated for $C_{27}H_{41}N_2O_5$ $[M + H]^+$: 473.30100, found 473.30111. Purity >95% by LC-MS, t_r = 7.75 minutes.

***N*-hydroxy-6-(4-(3-(5-(2-hydroxy-3,3-dimethylbutoxy)-6-methylpyridin-2-yl)pentan-3-yl)phenoxy)hexanamide (27c):** Synthesized following **General Procedure B** using hydroxylamine (50 wt% solution in H_2O, 13.1 mL, 435 mmol, 500 equiv), KOH (3.0 M solution in H_2O, 2.1 mL, 6.1 mmol, 7.0 equiv) and ethyl ester **26c** (0.45 g, 0.87 mmol, 1 equiv) in MeOH (8.7 mL). Purification by reversed-phase chromatography on octadecyl-functionalized silica gel using a gradient of 65–80% MeOH:H_2O over a period of 15 minutes as eluent to provide AM-193 (**27c**) as a fine white powder (0.25 g) in 55% yield after lyophilization. ^{1}H NMR (500 MHz, d_6-DMSO) δ 10.31 (s, 1H), δ 8.64 (s, 1H), δ 7.20 (d, *J* = 8.6 Hz, 1H), δ 6.99 (d, *J* = 8.8 Hz, 2H), δ 6.87 (d, *J* = 8.5 Hz, 1H), δ 6.77 (d, *J* = 8.8 Hz, 2H), 4.84 (d, *J* = 5.3 Hz, 1H), δ 4.04 (dd, *J* = 10.1, 2.9 Hz, 1H), δ 3.87 (t, *J* = 6.4 Hz, 2H), δ 3.75 (dd, *J* = 10.6, 7.9 Hz, 1H), δ 3.47–3.42 (m, 1H), δ 2.32 (s, 3H), δ 2.20–2.11 (m, 2H), δ 2.09–2.01 (m, 2H), δ 1.94 (t, *J* = 7.3 Hz, 2H), δ 1.66 (quintet, *J* = 6.7 Hz, 2H), δ 1.52 (quintet, *J* = 7.2 Hz, 2H), δ 1.40–1.31 (m, 2H), δ 0.91 (s, 9H), δ 0.53 (t, *J* = 7.3 Hz, 6H). ^{13}C NMR (125 MHz, $CDCl_3$) δ 170.9, 158.3, 156.6, 150.4, 146.8, 140.3, 128.9, 120.6, 117.7, 113.6, 77.3, 69.6, 67.4, 50.9, 33.7, 32.8, 28.8 (2C), 26.1, 25.6, 25.00, 19.5, 8.4. HRMS (ESI) calculated for $C_{29}H_{45}N_2O_5$ $[M + H]^+$: 501.33230, found 501.33219. Purity >95% by LC-MS, t_r = 8.32 minutes.

References

1. Song, S.; Sun, X.; Li, X.; Yuan, Y.; Jiao, N. *Org. Lett.* **2015**, *17* (12), 2886–2889.
2. Ungwitayatorn, J.; Wiwat, C.; Mataytsuk, C.; Pimthon, J.; Piyaviriyakul, S. *Chin. J. Chem.* **2008**, *26* (2), 379-387.
3. López-Rodríguez, M. L.; Viso, A.; Ortega-Gutiérrez, S.; Fowler, C. J.; Tiger, G.; de Lago, E.; Fernández-Ruiz, J.; Ramos, J. A. *J. Med. Chem.* **2003**, *46* (8), 1512–1522.
4. Kuwano, R.; Kusano, H. *Org. Lett.* **2008**, *10* (10), 1979–1982.
5. Kaldre, D.; Wang, T.-T.; Fischer, J.; White, J.-H.; Gleason, J.-L. *Bioorg. Med. Chem.* **2015**, *23* (15), 5035–5049.

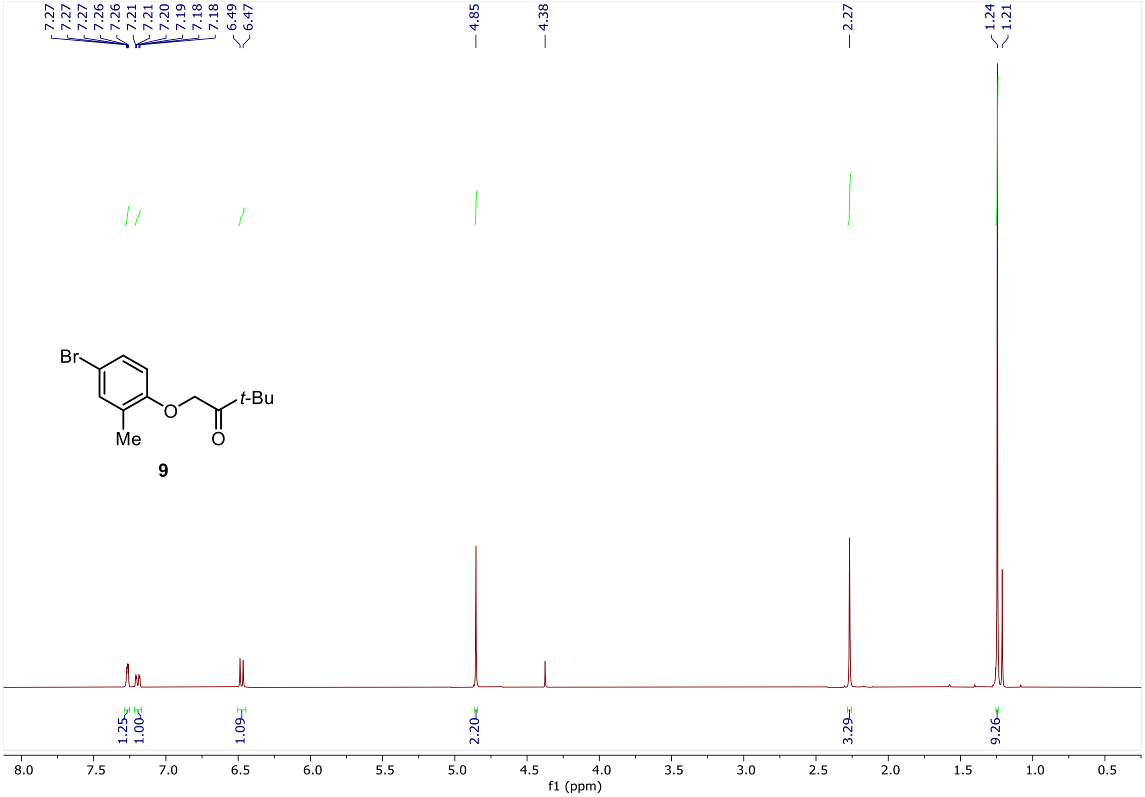
7.27
7.27
7.27
7.26
7.26
7.21
7.21
7.20
7.19
7.18
7.18
6.49
6.47
4.85
4.38
2.27
1.24
1.21
Br
O
t-Bu
Me
O
9
1.25
1.00
1.09
2.20
3.29
9.26
8.0 7.5 7.0 6.5 6.0 5.5 5.0 4.5 4.0 3.5 3.0 2.5 2.0 1.5 1.0 0.5
f1 (ppm)

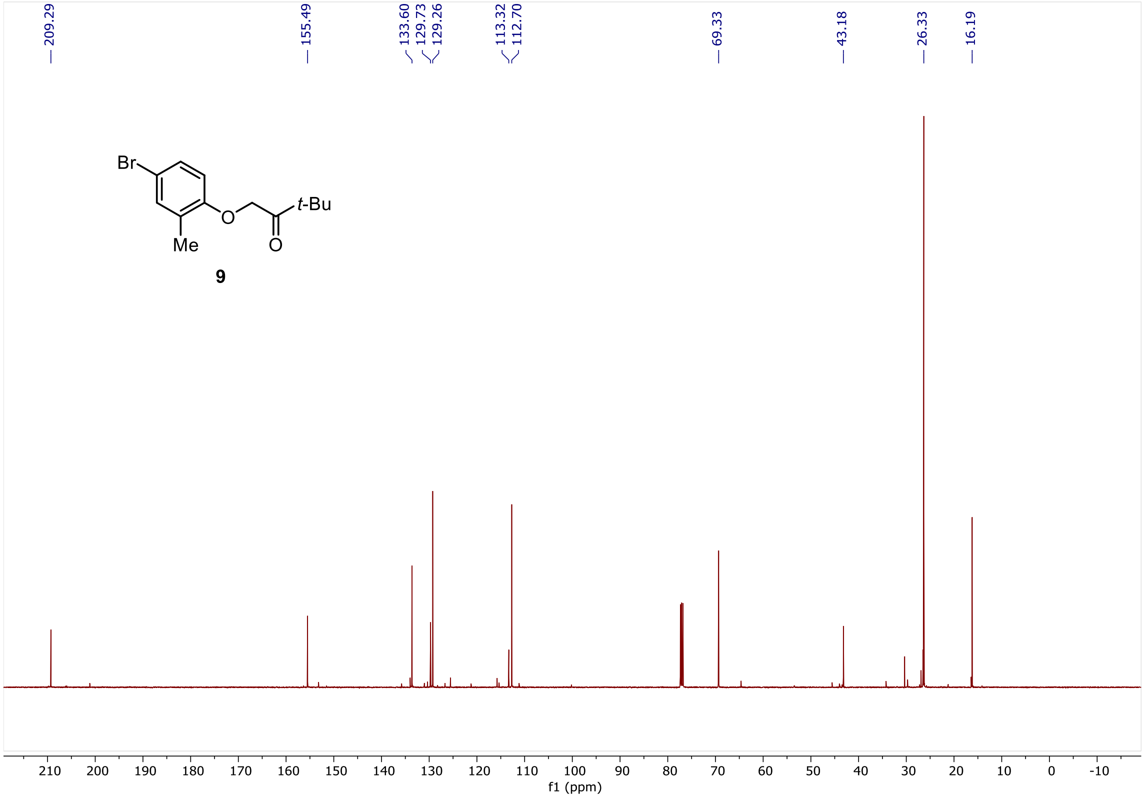
209.29
155.49
133.60
129.73
129.26
113.32
112.70
69.33
43.18
26.33
16.19
Br
O
t-Bu
Me
O
9
210 200 190 180 170 160 150 140 130 120 110 100 90 80 70 60 50 40 30 20 10 0 -10
f1 (ppm)

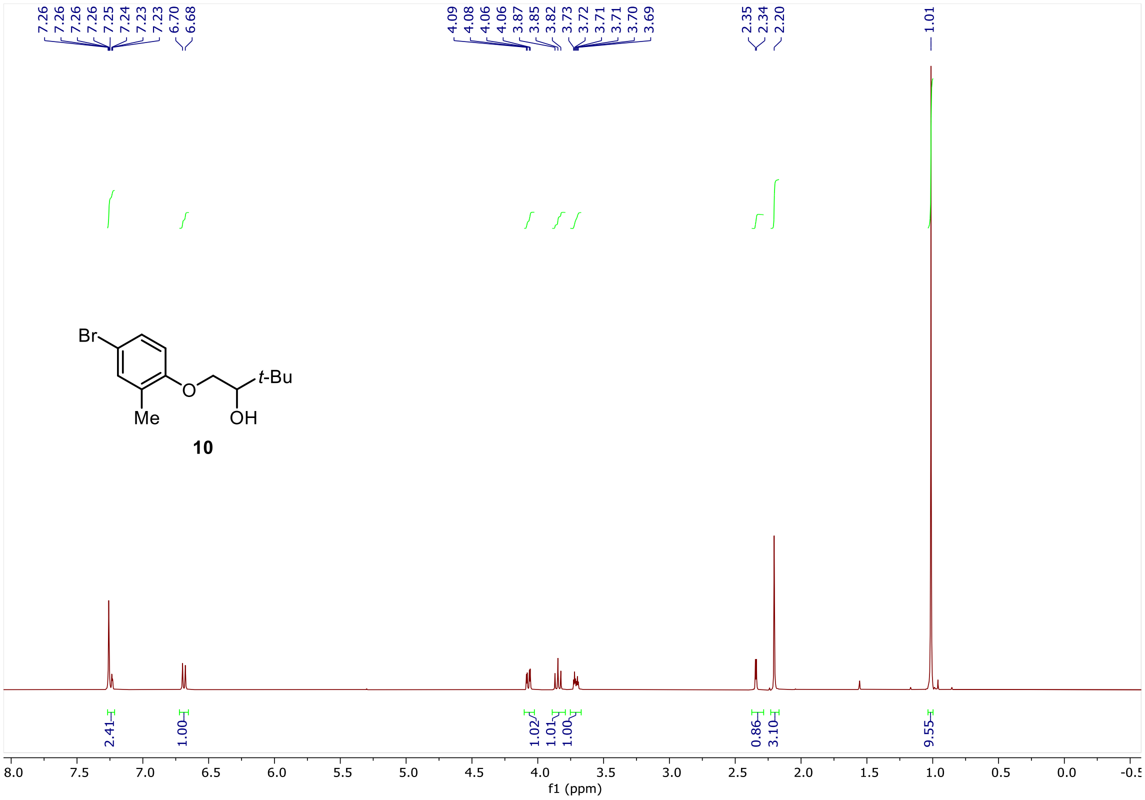
7.26 7.26 7.26 7.26 7.25 7.24 7.23 7.23 6.70 6.68
4.09 4.08 4.06 4.06 3.87 3.85 3.82 3.73 3.72 3.71 3.71 3.70 3.69
2.35 2.34 2.20
1.01
Br
O
t-Bu
Me
OH
10
2.41
1.00
1.02
1.01
1.00
0.86
3.10
9.55
8.0 7.5 7.0 6.5 6.0 5.5 5.0 4.5 4.0 3.5 3.0 2.5 2.0 1.5 1.0 0.5 0.0
f1 (ppm)

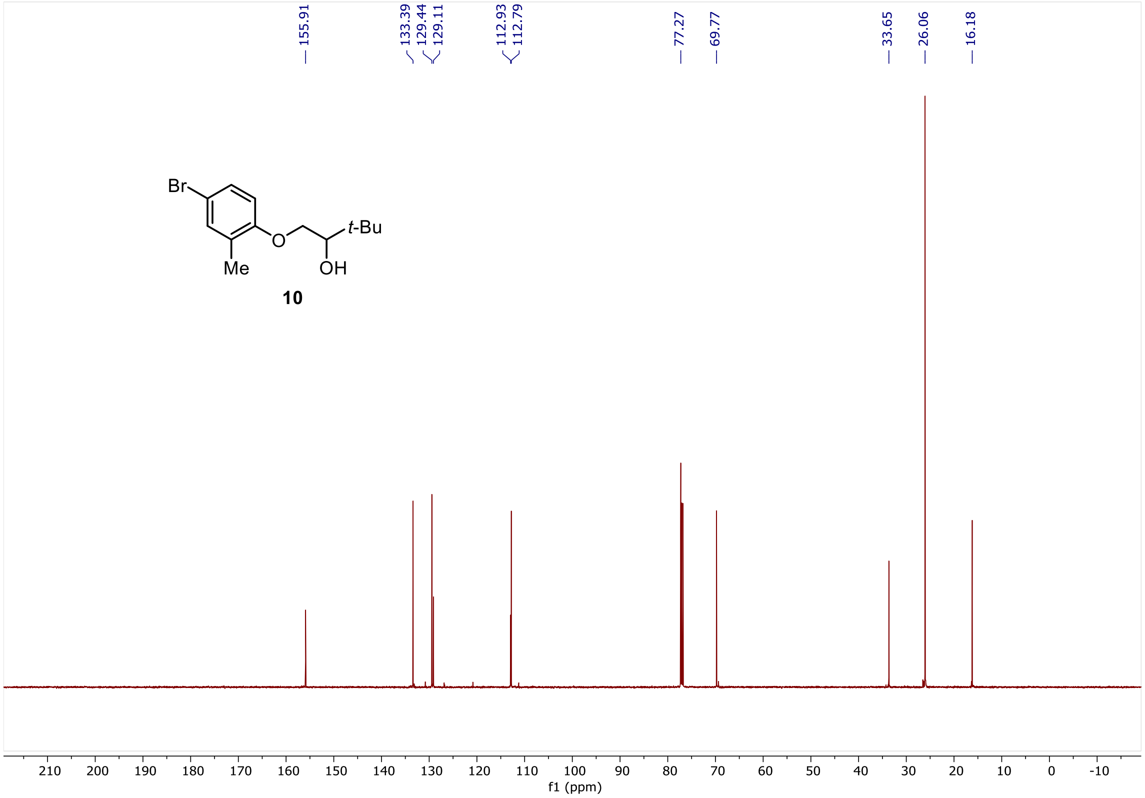
155.91
133.39 129.44 129.11
112.93 112.79
77.27
69.77
33.65
26.06
16.18
Br
O
t-Bu
Me
OH
10
210 200 190 180 170 160 150 140 130 120 110 100 90 80 70 60 50 40 30 20 10 0 -10
f1 (ppm)

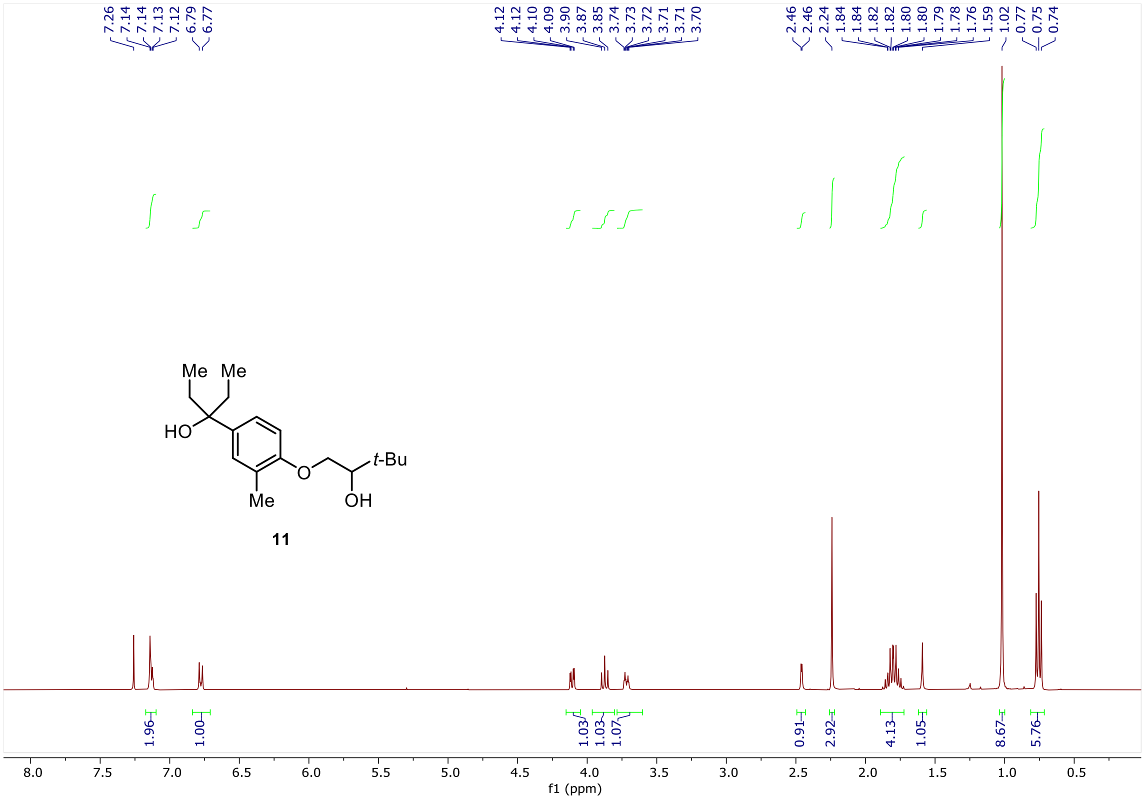
7.26
7.14
7.14
7.13
7.12
6.79
6.77
4.12
4.12
4.10
4.09
3.90
3.87
3.85
3.74
3.73
3.72
3.71
3.71
3.70
2.46
2.46
2.24
1.84
1.84
1.82
1.82
1.80
1.80
1.79
1.78
1.76
1.59
1.02
0.77
0.75
0.74
Me
Me
HO
O
t-Bu
Me
OH
11
1.96
1.00
1.03
1.03
1.07
0.91
2.92
4.13
1.05
8.67
5.76
f1 (ppm)

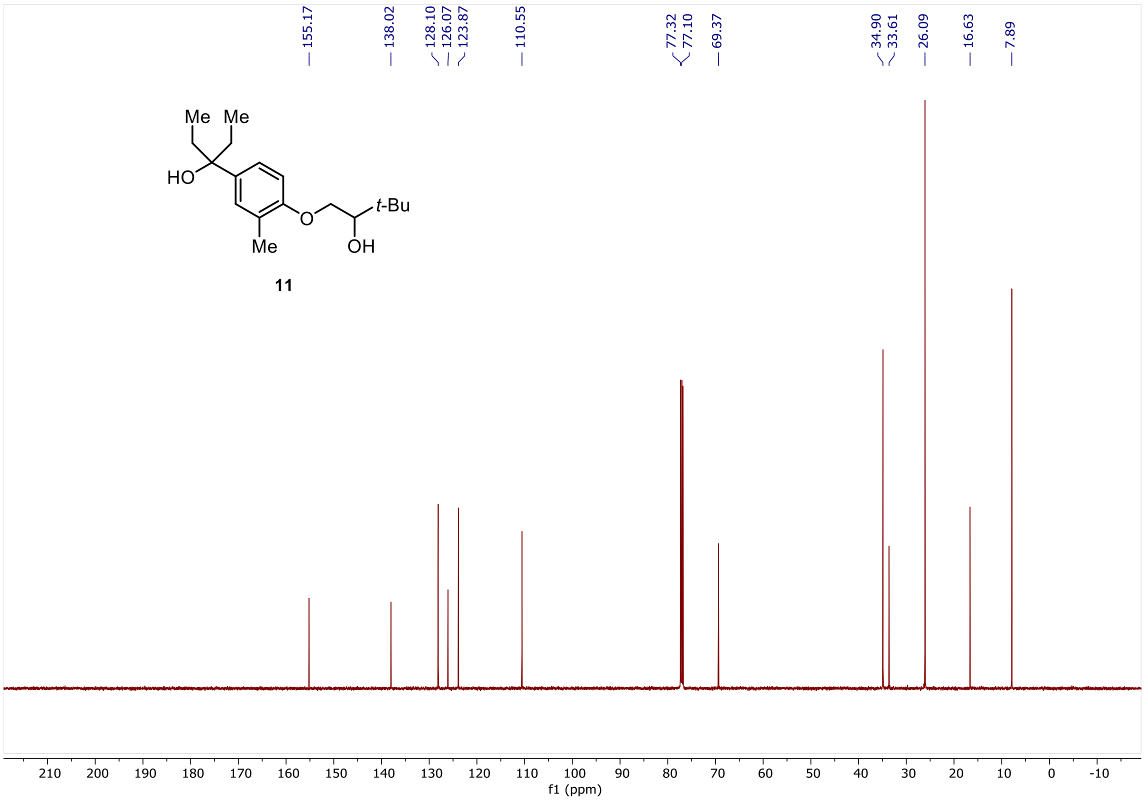
155.17
138.02
128.10
126.07
123.87
110.55
77.32
77.10
69.37
34.90
33.61
26.09
16.63
7.89
Me
Me
HO
O
t-Bu
Me
OH
11
f1 (ppm)

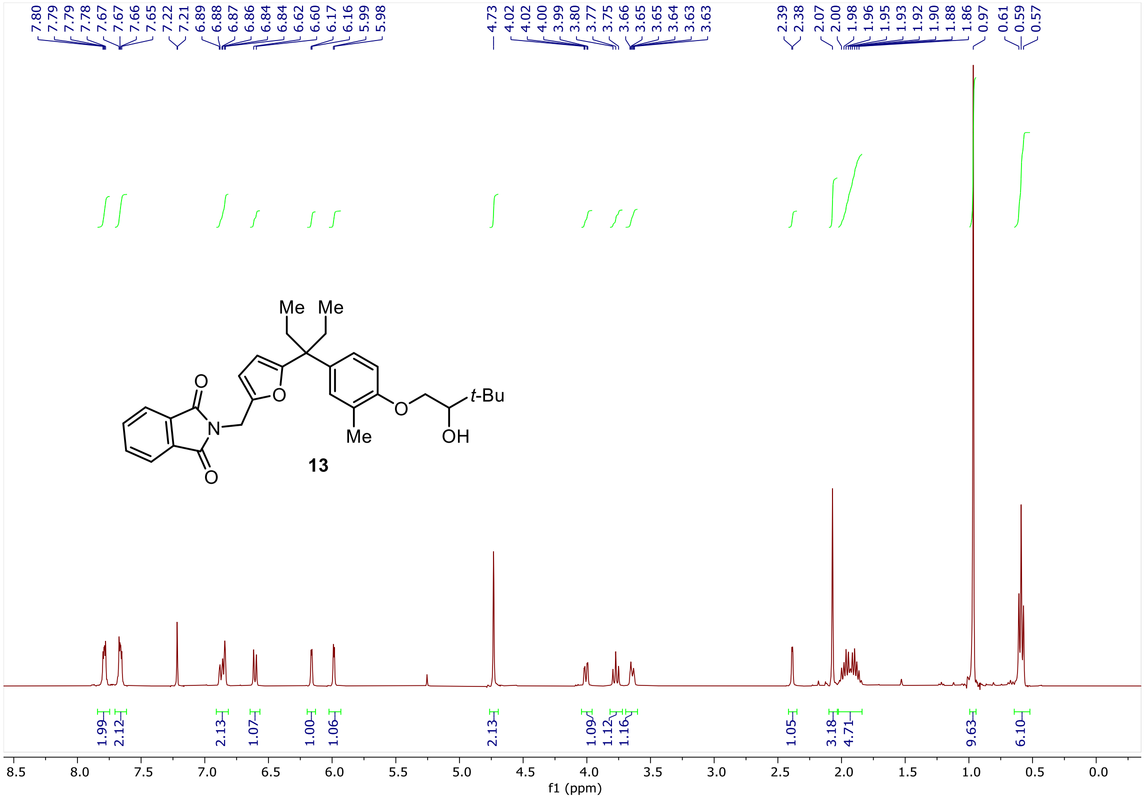
7.80
7.79
7.79
7.78
7.67
7.67
7.66
7.65
7.22
7.21
6.89
6.88
6.87
6.86
6.84
6.84
6.62
6.60
6.17
6.16
5.99
5.98
4.73
4.02
4.02
4.00
3.99
3.80
3.77
3.75
3.66
3.65
3.65
3.64
3.63
3.63
2.39
2.38
2.07
2.00
1.98
1.96
1.95
1.93
1.92
1.90
1.88
1.86
0.97
0.61
0.59
0.57
Me Me
O
N
O
O
O
t-Bu
Me
OH
13
1.99
2.12
2.13
1.07
1.00
1.06
2.13
1.09
1.12
1.16
1.05
3.18
4.71
9.63
6.10
8.5 8.0 7.5 7.0 6.5 6.0 5.5 5.0 4.5 4.0 3.5 3.0 2.5 2.0 1.5 1.0 0.5 0.0
f1 (ppm)

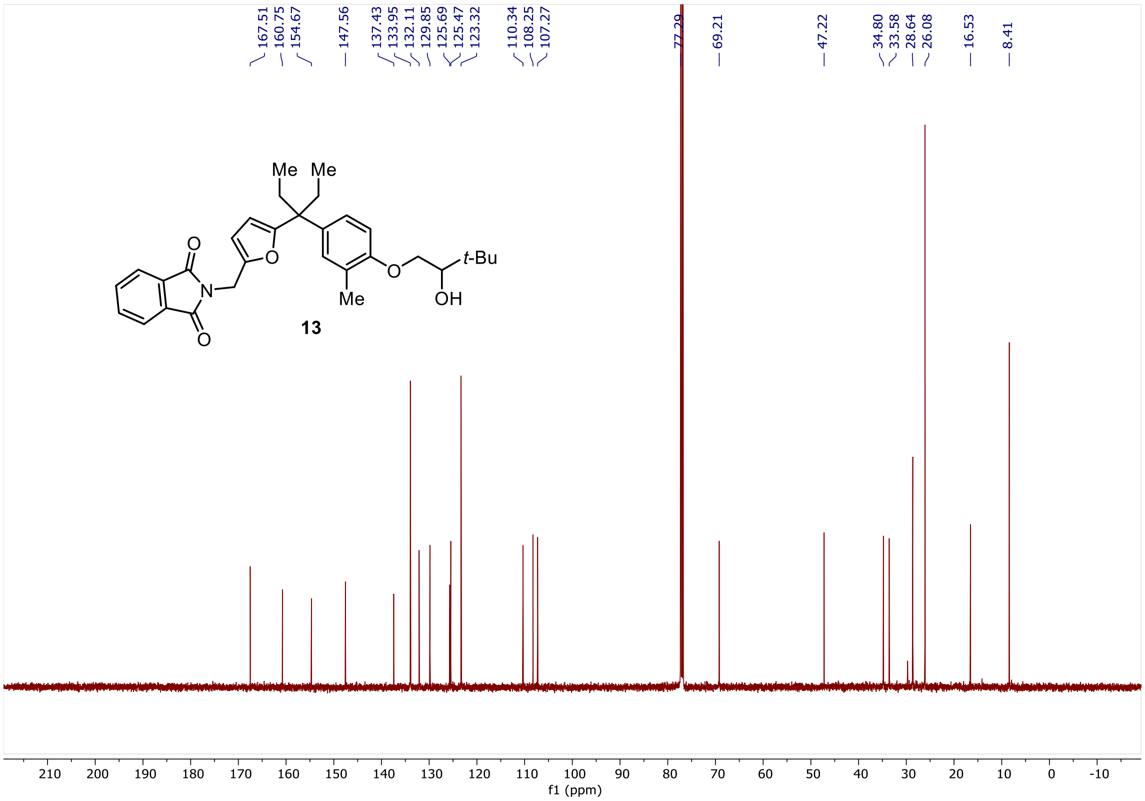
167.51
160.75
154.67
147.56
137.43
133.95
132.11
129.85
125.69
125.47
123.32
110.34
108.25
107.27
77.29
69.21
47.22
34.80
33.58
28.64
26.08
16.53
8.41
Me Me
O
N
O
O
O
t-Bu
Me
OH
13
210 200 190 180 170 160 150 140 130 120 110 100 90 80 70 60 50 40 30 20 10 0 -10
f1 (ppm)

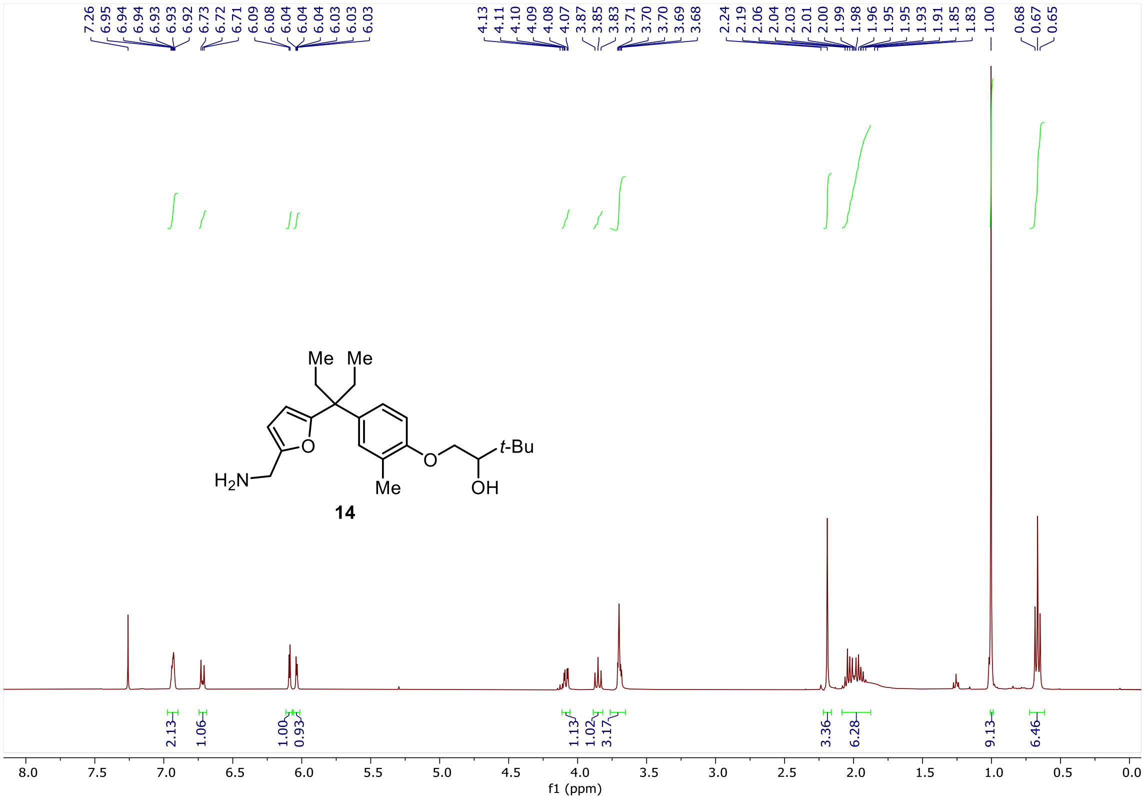
7.26
6.95
6.94
6.94
6.93
6.93
6.92
6.73
6.72
6.71
6.09
6.08
6.04
6.04
6.04
6.03
6.03
6.03
4.13
4.11
4.10
4.09
4.08
4.07
3.87
3.85
3.83
3.71
3.70
3.70
3.69
3.68
2.24
2.19
2.06
2.04
2.03
2.01
2.00
1.99
1.98
1.96
1.95
1.95
1.93
1.91
1.85
1.83
1.00
0.68
0.67
0.65
Me
Me
O
H_2N
Me
O
t-Bu
OH
14
2.13
1.06
1.00
0.93
1.13
1.02
3.17
3.36
6.28
9.13
6.46
8.0
7.5
7.0
6.5
6.0
5.5
5.0
4.5
4.0
3.5
3.0
2.5
2.0
1.5
1.0
0.5
0.0
f1 (ppm)

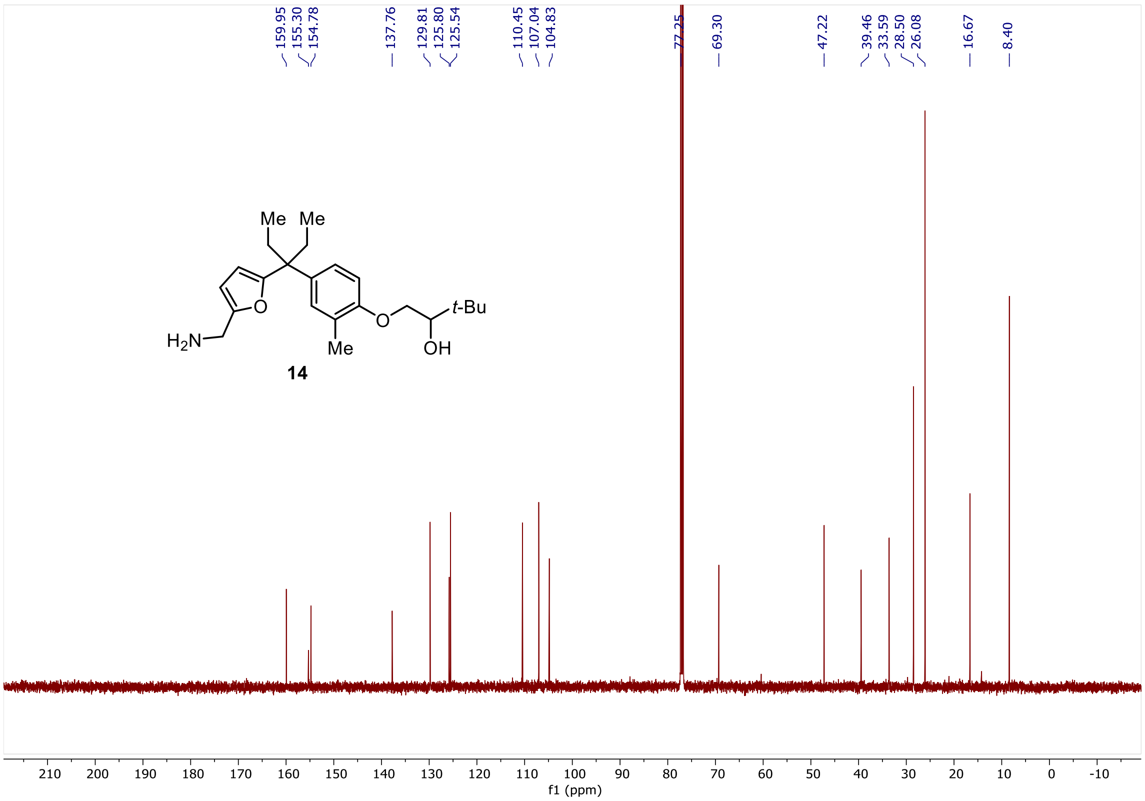
159.95
155.30
154.78
137.76
129.81
125.80
125.54
110.45
107.04
104.83
77.25
69.30
47.22
39.46
33.59
28.50
26.08
16.67
8.40
Me
Me
O
H_2N
Me
O
t-Bu
OH
14
210
200
190
180
170
160
150
140
130
120
110
100
90
80
70
60
50
40
30
20
10
0
-10
f1 (ppm)

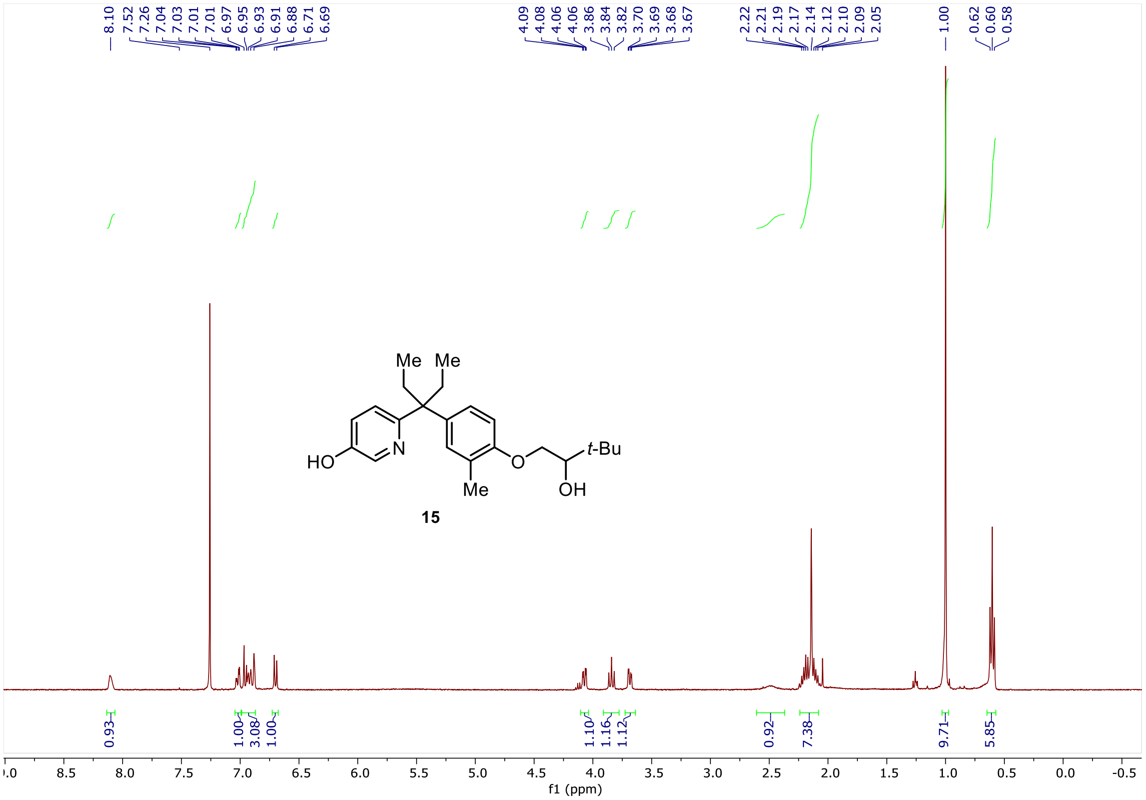
8.10
7.52 7.26 7.04 7.03 7.01 7.01 6.97 6.95 6.93 6.91 6.88 6.71 6.69
4.09 4.08 4.06 4.06 3.86 3.84 3.82 3.70 3.69 3.68 3.67
2.22 2.21 2.19 2.17 2.14 2.12 2.10 2.09 2.05
1.00
0.62 0.60 0.58
Me Me
HO
N
O
t-Bu
Me
OH
15
0.93 1.00 3.08 1.00 1.10 1.16 1.12 0.92 7.38 9.71 5.85
f1 (ppm)

15

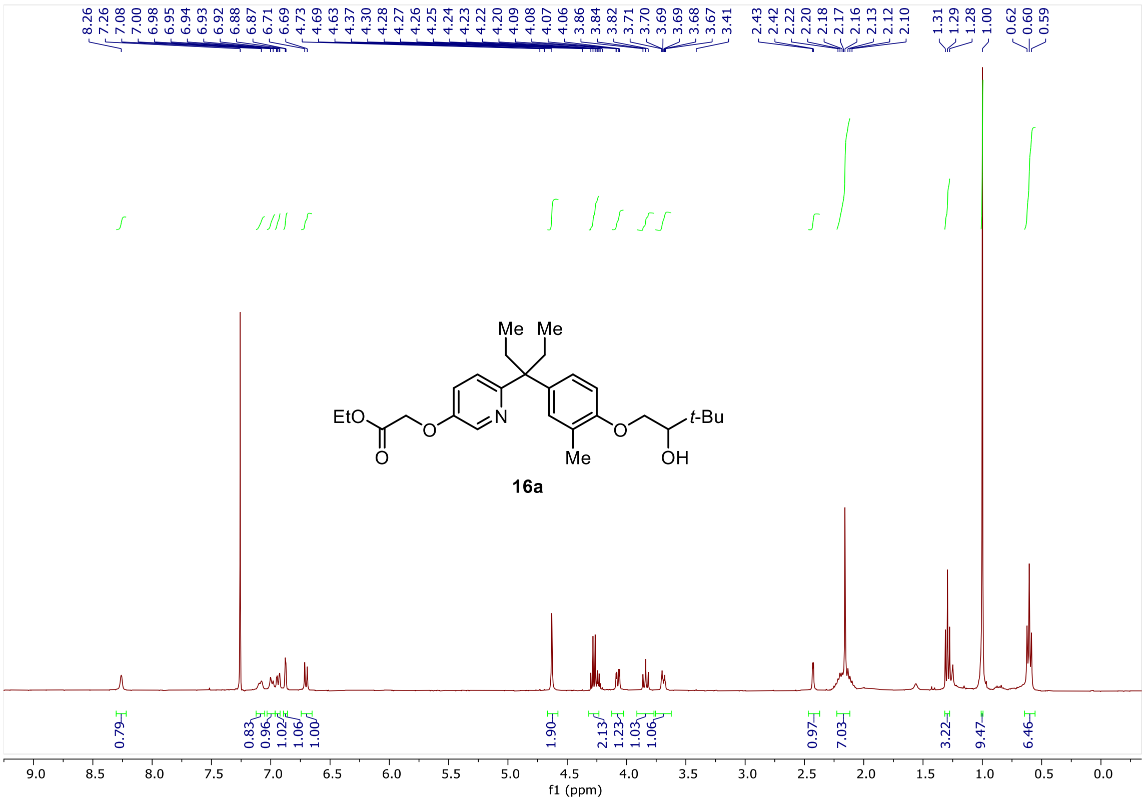
8.26 7.26 7.08 7.00 6.98 6.95 6.94 6.93 6.92 6.88 6.87 6.71 6.69 4.73 4.69 4.63 4.37 4.30 4.28 4.27 4.26 4.25 4.24 4.23 4.22 4.20 4.09 4.08 4.07 4.06 3.86 3.84 3.82 3.71 3.70 3.69 3.69 3.68 3.67 3.41
2.43 2.42 2.22 2.20 2.18 2.17 2.16 2.13 2.12 2.10
1.31 1.29 1.28 1.00
0.62 0.60 0.59
Me Me
EtO
O
O
N
O
t-Bu
Me
OH
16a
0.79 0.83 0.96 1.02 1.06 1.00 1.90 2.13 1.23 1.03 1.06 0.97 7.03 3.22 9.47 6.46
f1 (ppm)

16a

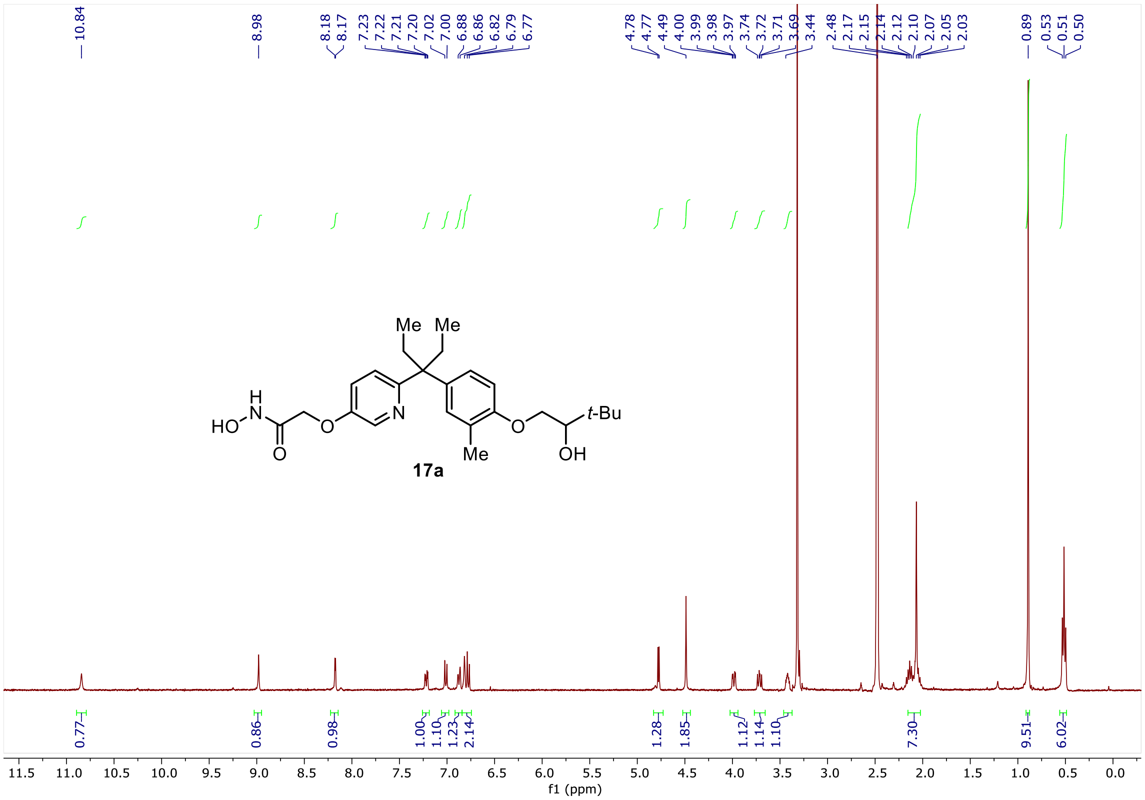
10.84
8.98
8.18
8.17
7.23
7.22
7.21
7.20
7.02
7.00
6.88
6.86
6.82
6.79
6.77
4.78
4.77
4.49
4.00
3.99
3.98
3.97
3.74
3.72
3.71
3.69
3.44
2.48
2.17
2.15
2.14
2.12
2.10
2.07
2.05
2.03
0.89
0.53
0.51
0.50
Me
Me
HO
N
H
O
O
N
O
t-Bu
Me
OH
17a
0.77
0.86
0.98
1.00
1.10
1.23
2.14
1.28
1.85
1.12
1.14
1.10
7.30
9.51
6.02
11.5 11.0 10.5 10.0 9.5 9.0 8.5 8.0 7.5 7.0 6.5 6.0 5.5 5.0 4.5 4.0 3.5 3.0 2.5 2.0 1.5 1.0 0.5 0.0
f1 (ppm)

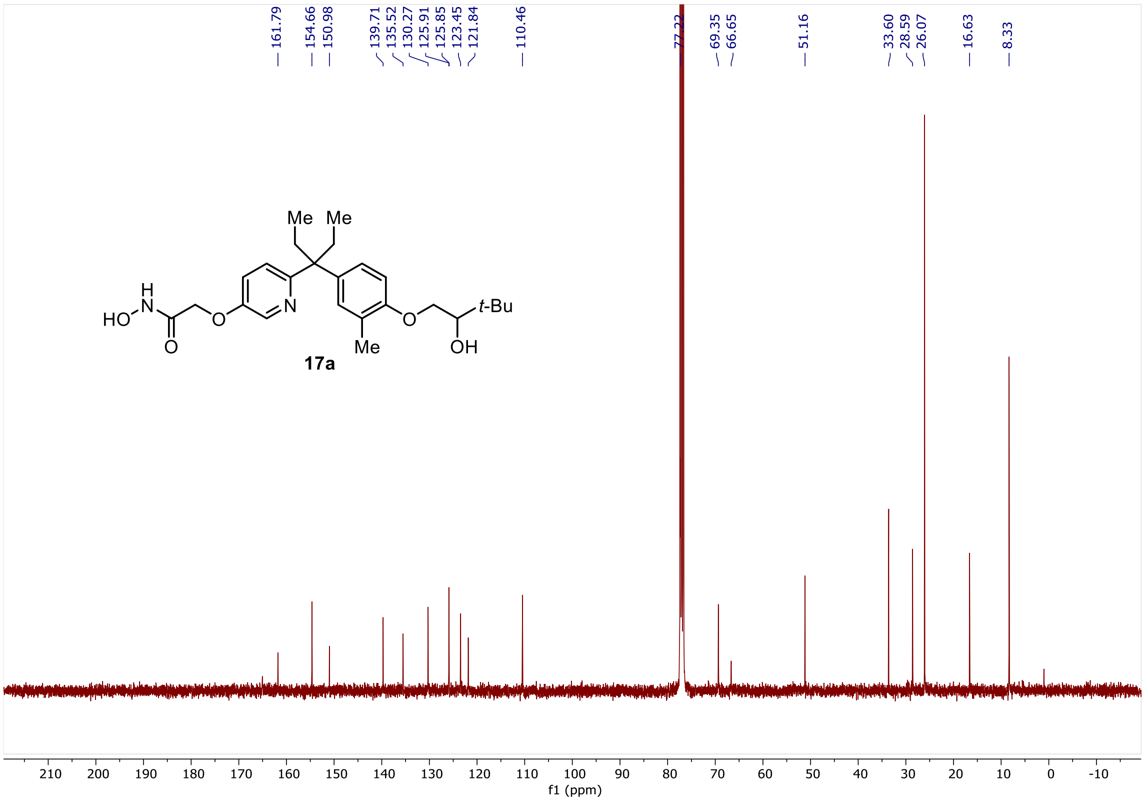
161.79
154.66
150.98
139.71
135.52
130.27
125.91
125.85
123.45
121.84
110.46
77.22
69.35
66.65
51.16
33.60
28.59
26.07
16.63
8.33
Me
Me
HO
N
H
O
O
N
O
t-Bu
Me
OH
17a
210 200 190 180 170 160 150 140 130 120 110 100 90 80 70 60 50 40 30 20 10 0 -10
f1 (ppm)

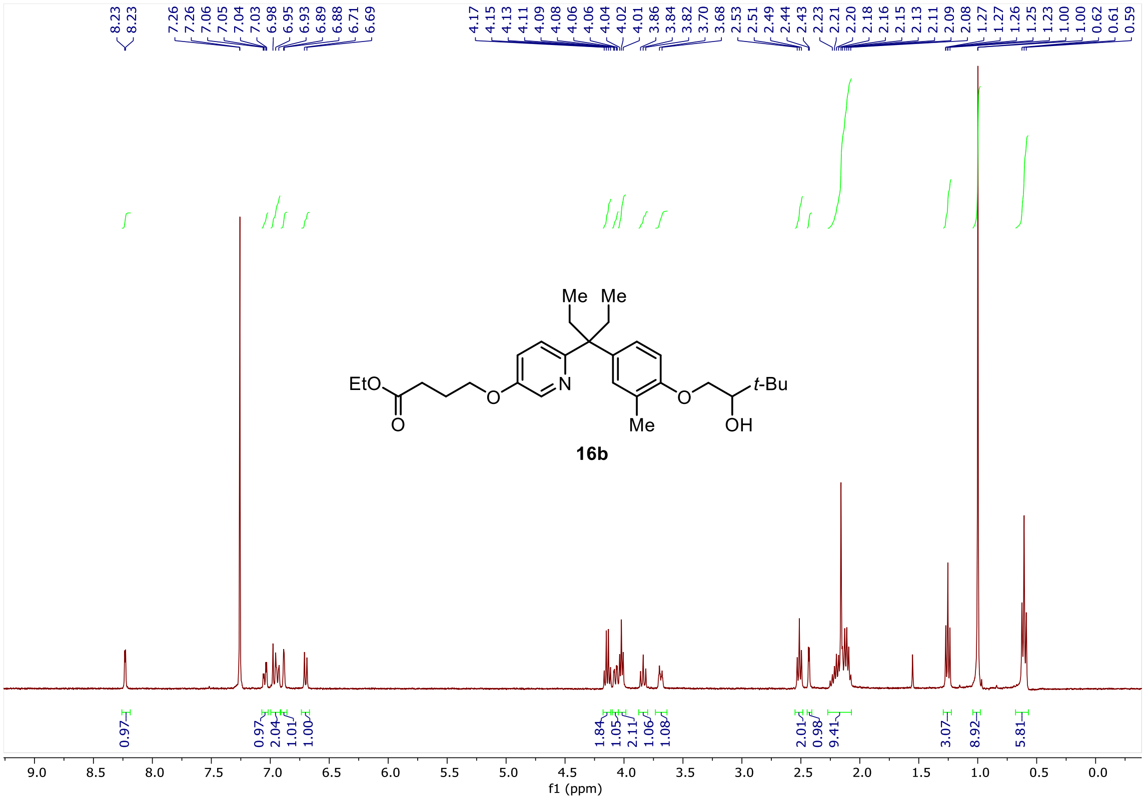
16b
EtO
O
N
Me
Me
Me
OH
t-Bu
f1 (ppm)

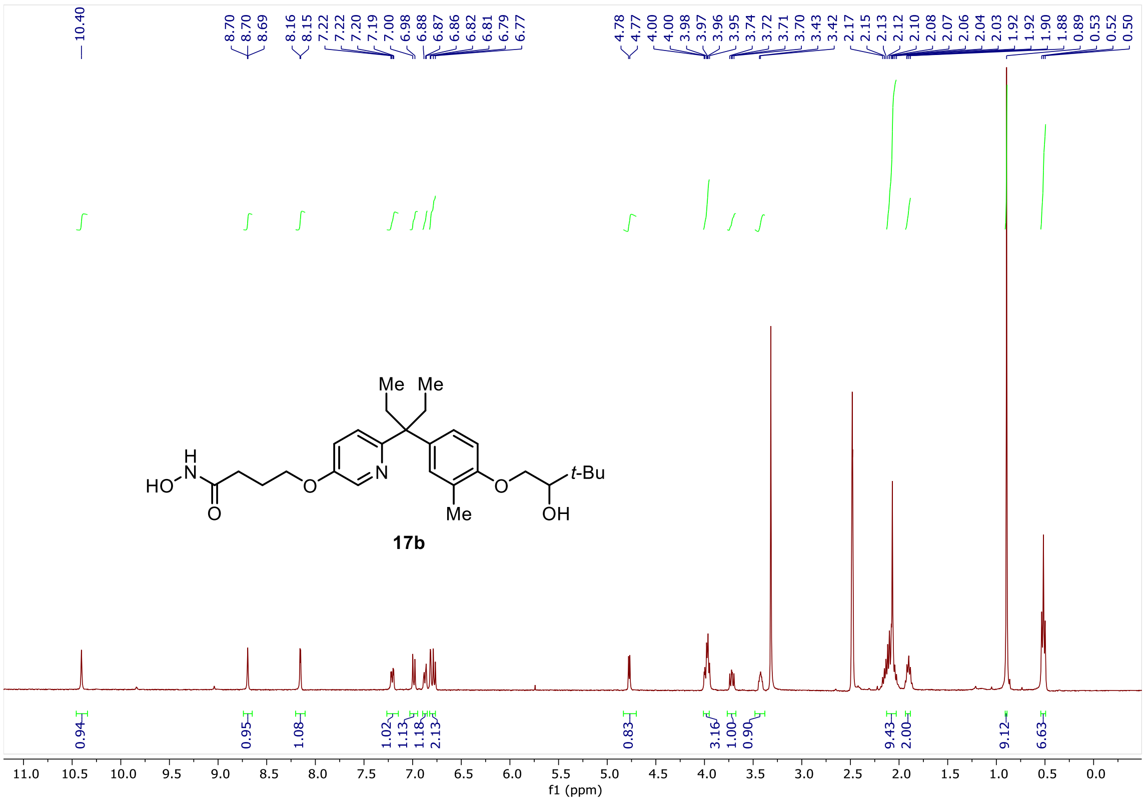
17b
HO
H
N
O
Me
Me
Me
OH
t-Bu
f1 (ppm)

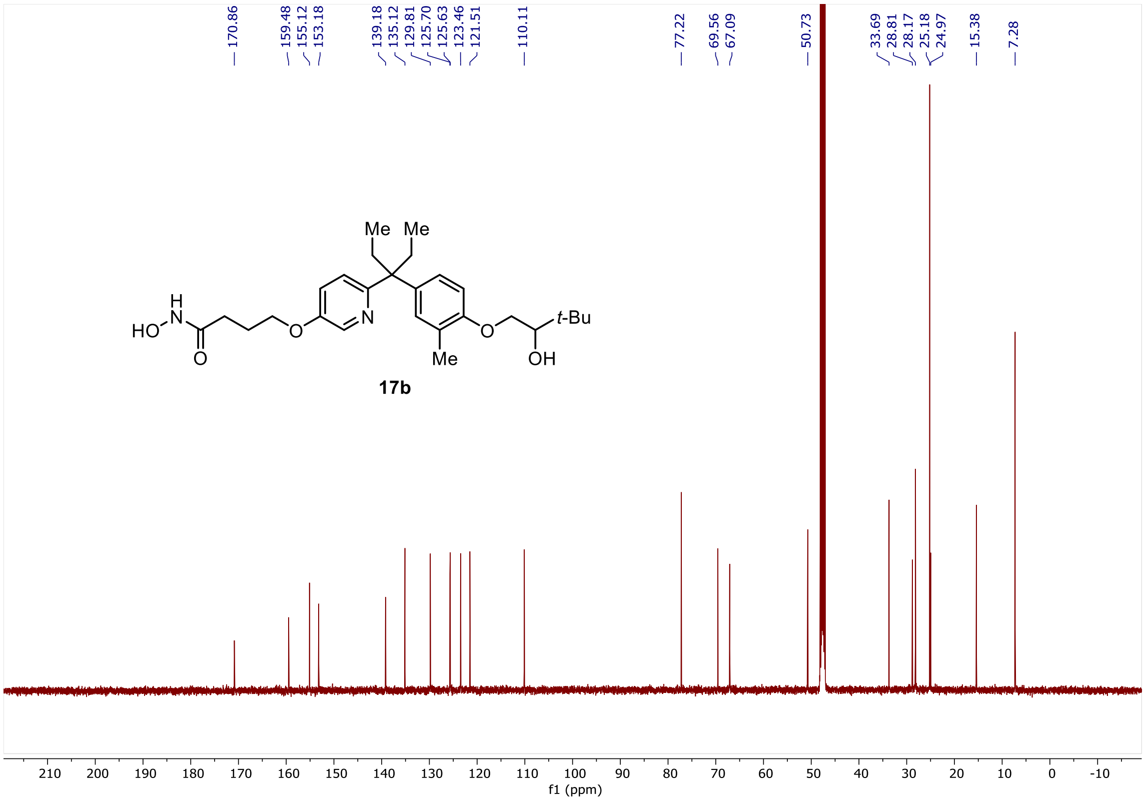
170.86
159.48
155.12
153.18
139.18
135.12
129.81
125.70
125.63
123.46
121.51
110.11
77.22
69.56
67.09
50.73
33.69
28.81
28.17
25.18
24.97
15.38
7.28
Me Me
HO
H
N
O
O
N
O
t-Bu
Me
OH
17b
210 200 190 180 170 160 150 140 130 120 110 100 90 80 70 60 50 40 30 20 10 0 -10
f1 (ppm)

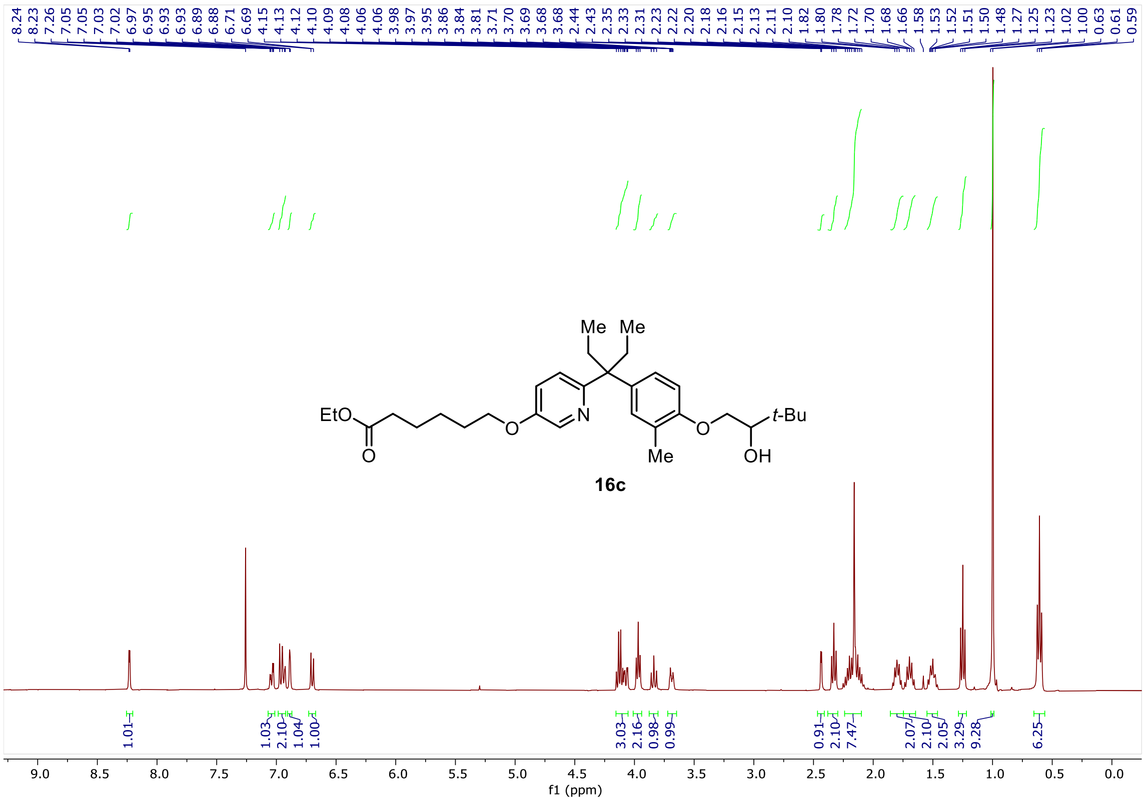
8.24 8.23 7.26 7.05 7.03 7.02 6.97 6.95 6.93 6.93 6.89 6.88 6.71 6.69 4.15 4.13 4.12 4.10 4.09 4.08 4.06 4.06 3.98 3.97 3.95 3.86 3.84 3.81 3.71 3.70 3.69 3.68 2.44 2.43 2.35 2.33 2.31 2.23 2.22 2.20 2.18 2.16 2.15 2.13 2.11 2.10 1.82 1.80 1.78 1.72 1.70 1.68 1.66 1.58 1.53 1.52 1.51 1.50 1.48 1.27 1.25 1.23 1.02 1.00 0.63 0.61 0.59
Me Me
EtO
O
O
N
O
t-Bu
Me
OH
16c
1.01 1.03 2.10 1.04 1.00 3.03 2.16 0.98 0.99 0.91 2.10 7.47 2.07 2.10 2.05 3.29 9.28 6.25
9.0 8.5 8.0 7.5 7.0 6.5 6.0 5.5 5.0 4.5 4.0 3.5 3.0 2.5 2.0 1.5 1.0 0.5 0.0
f1 (ppm)

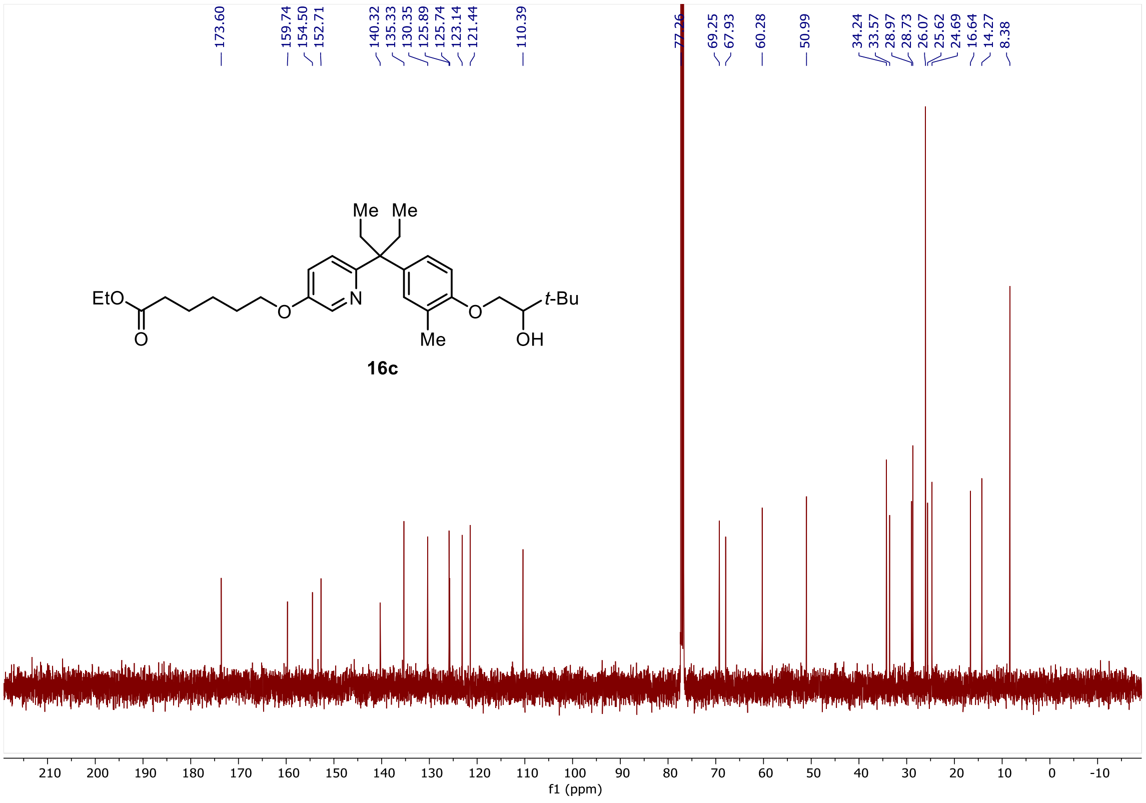
173.60
159.74
154.50
152.71
140.32
135.33
130.35
125.89
125.74
123.14
121.44
110.39
77.26
69.25
67.93
60.28
50.99
34.24
33.57
28.97
28.73
26.07
25.62
24.69
16.64
14.27
8.38
Me Me
EtO
O
O
N
O
t-Bu
Me
OH
16c
f1 (ppm)

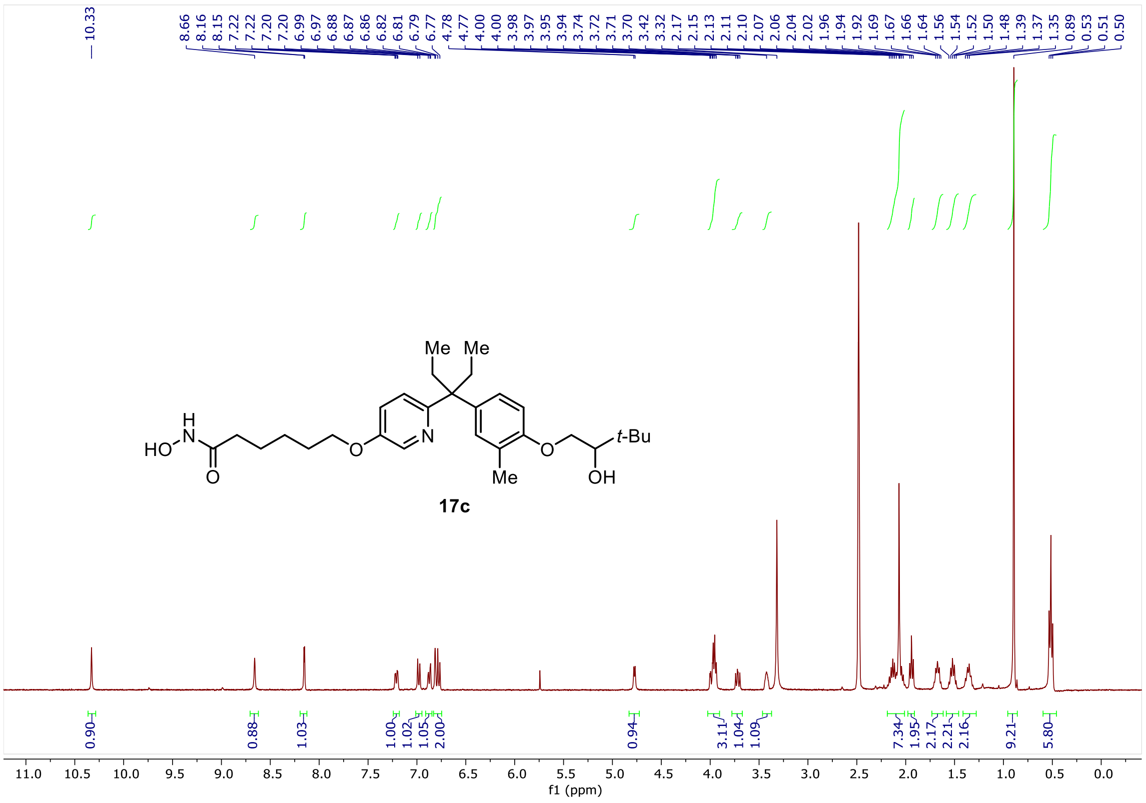
10.33
8.66
8.16
8.15
7.22
7.22
7.20
7.20
6.99
6.97
6.88
6.87
6.86
6.82
6.81
6.79
6.77
4.78
4.77
4.00
4.00
3.98
3.97
3.95
3.94
3.74
3.72
3.71
3.70
3.42
3.32
2.17
2.15
2.13
2.11
2.10
2.07
2.06
2.04
2.02
1.96
1.94
1.92
1.69
1.67
1.66
1.64
1.56
1.54
1.52
1.50
1.48
1.39
1.37
1.35
0.89
0.53
0.51
0.50
Me Me
H
HO N
O
O
N
O
t-Bu
Me
OH
17c
0.90
0.88
1.03
1.00
1.02
1.05
2.00
0.94
3.11
1.04
1.09
7.34
1.95
2.17
2.21
2.16
9.21
5.80
f1 (ppm)

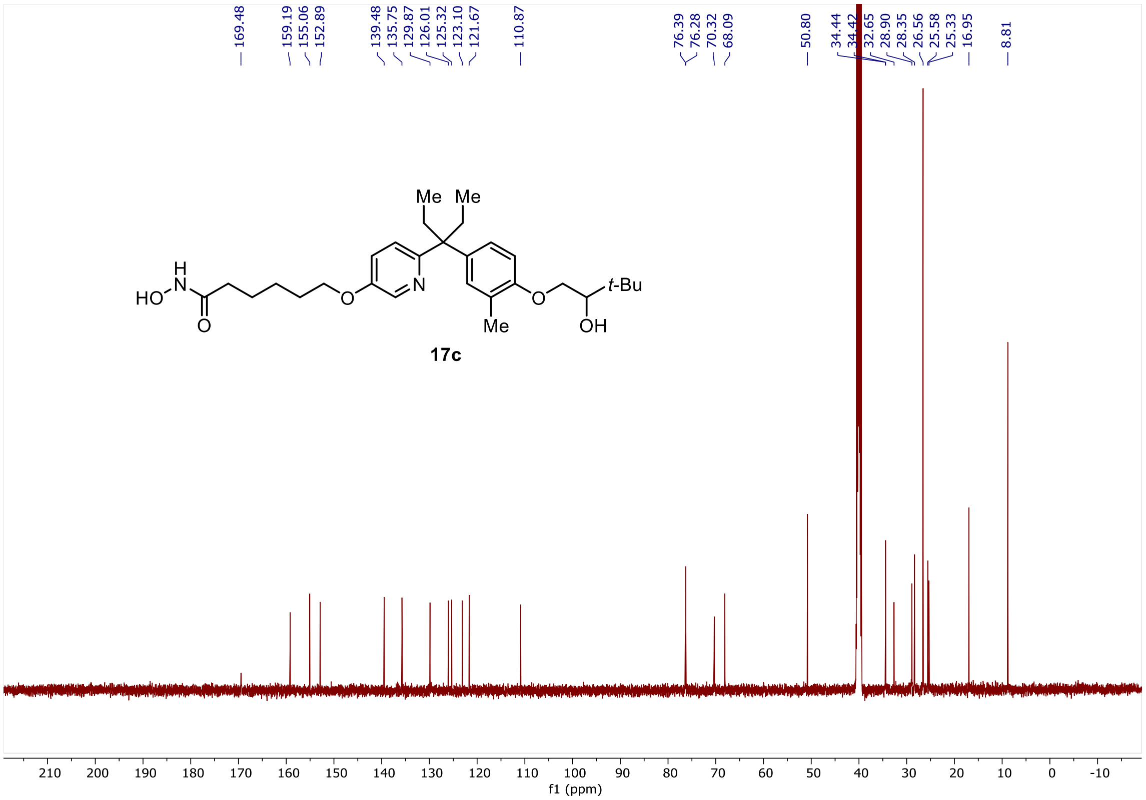
169.48
159.19
155.06
152.89
139.48
135.75
129.87
126.01
125.32
123.10
121.67
110.87
76.39
76.28
70.32
68.09
50.80
34.44
34.42
32.65
28.90
28.35
26.56
25.58
25.33
16.95
8.81
Me Me
HO N H O O N O Me OH t-Bu
17c
210 200 190 180 170 160 150 140 130 120 110 100 90 80 70 60 50 40 30 20 10 0 -10
f1 (ppm)

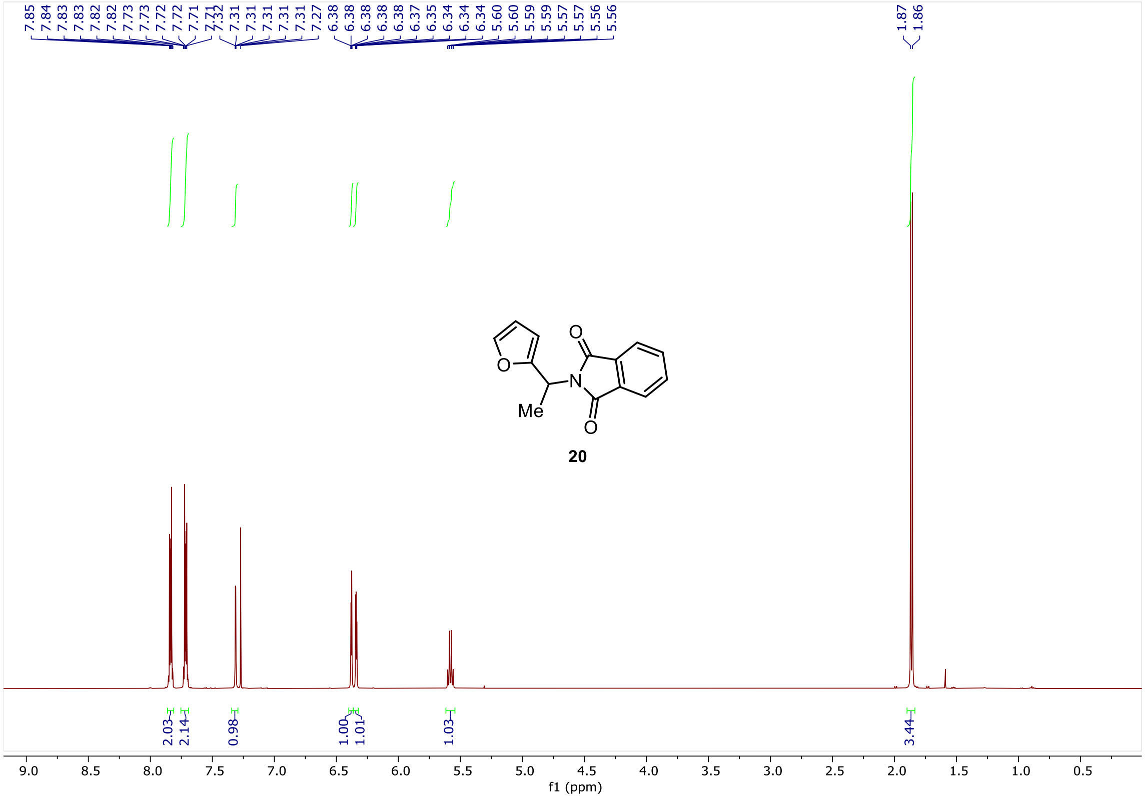
7.85
7.84
7.83
7.83
7.82
7.82
7.73
7.73
7.72
7.71
7.71
7.32
7.31
7.31
7.31
7.31
7.31
7.27
6.38
6.38
6.38
6.38
6.38
6.37
6.35
6.34
6.34
6.34
5.60
5.60
5.59
5.59
5.57
5.57
5.56
5.56
1.87
1.86
O N O O Me
20
2.03
2.14
0.98
1.00
1.01
1.03
3.44
9.0 8.5 8.0 7.5 7.0 6.5 6.0 5.5 5.0 4.5 4.0 3.5 3.0 2.5 2.0 1.5 1.0 0.5
f1 (ppm)

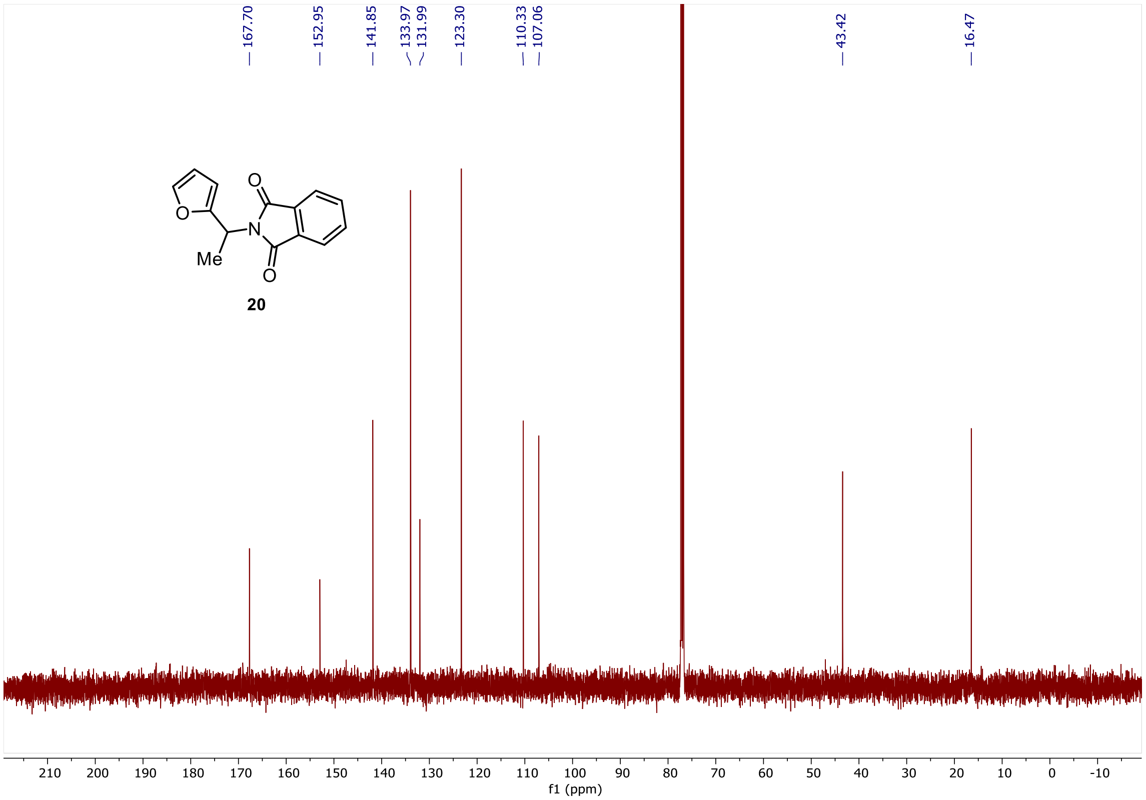
167.70
152.95
141.85
133.97
131.99
123.30
110.33
107.06
43.42
16.47
Me
20
f1 (ppm)

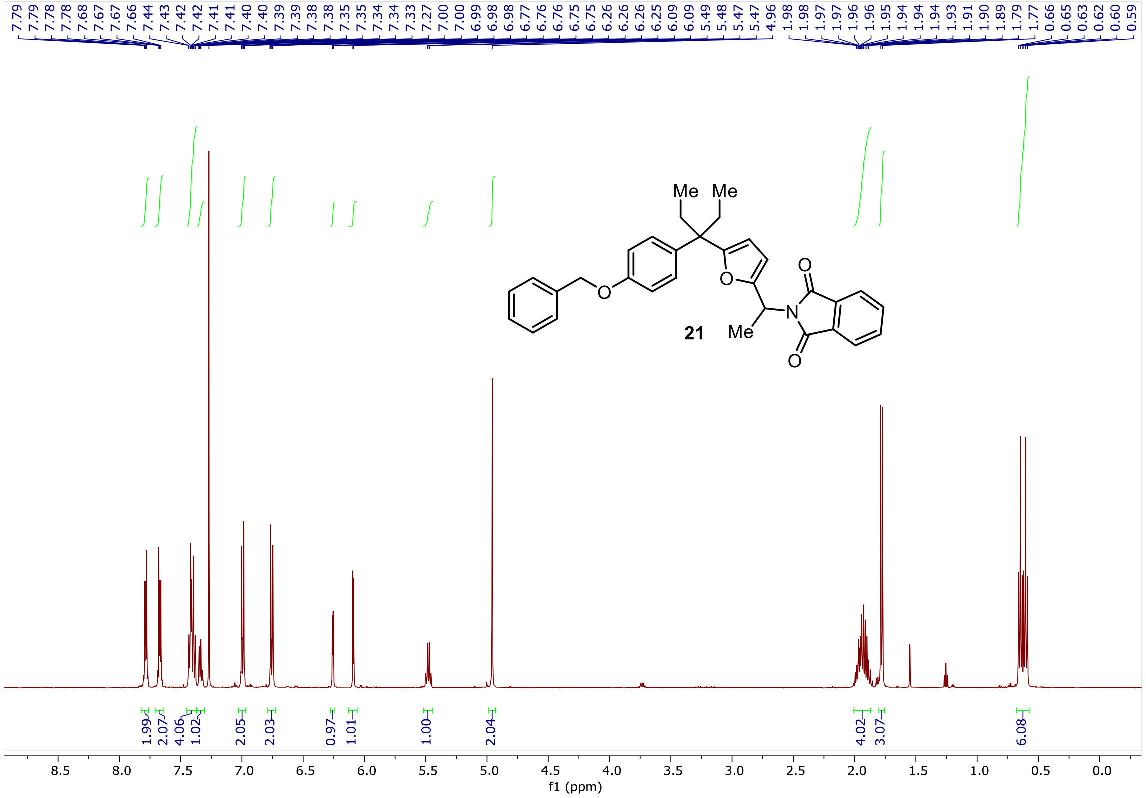
Me
Me
21
Me
1.99
2.07
4.06
1.02
2.05
2.03
0.97
1.01
1.00
2.04
4.02
3.07
6.08
f1 (ppm)

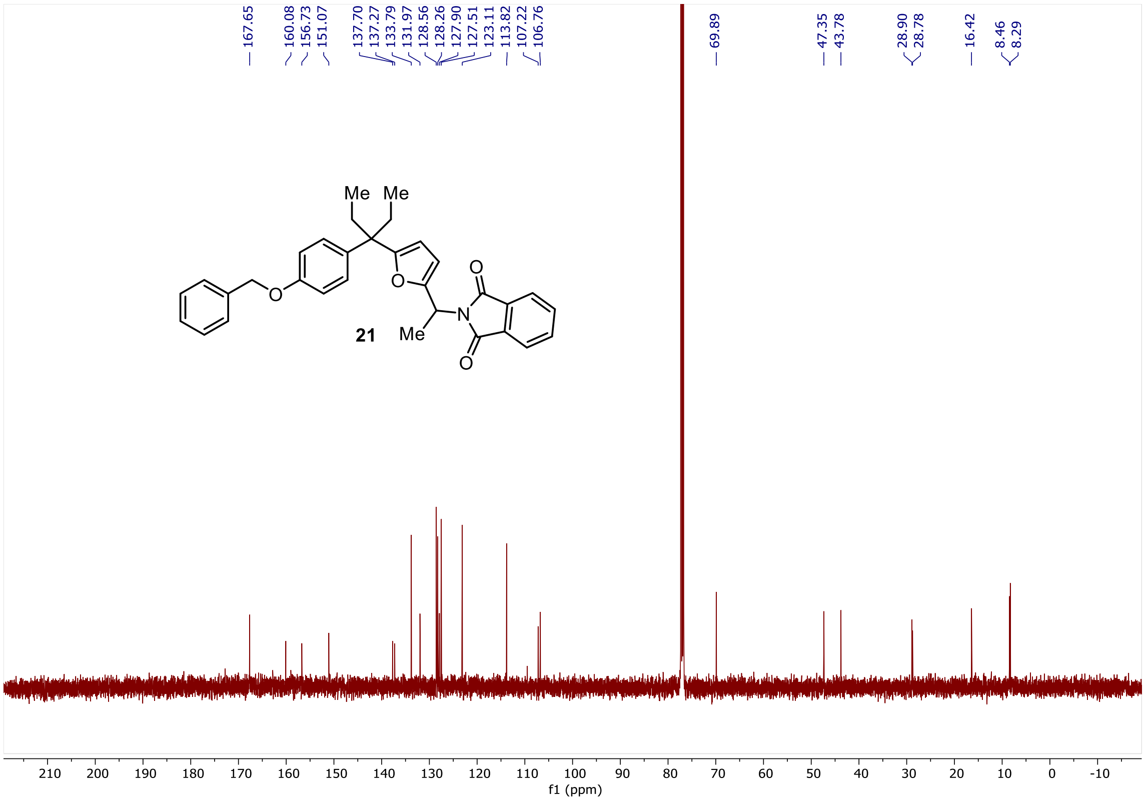

167.65
160.08
156.73
151.07
137.70
137.27
133.79
131.97
128.56
128.26
127.90
127.51
123.11
113.82
107.22
106.76
69.89
47.35
43.78
28.90
28.78
16.42
8.46
8.29
Me
Me
O
O
N
O
Me
21
f1 (ppm)

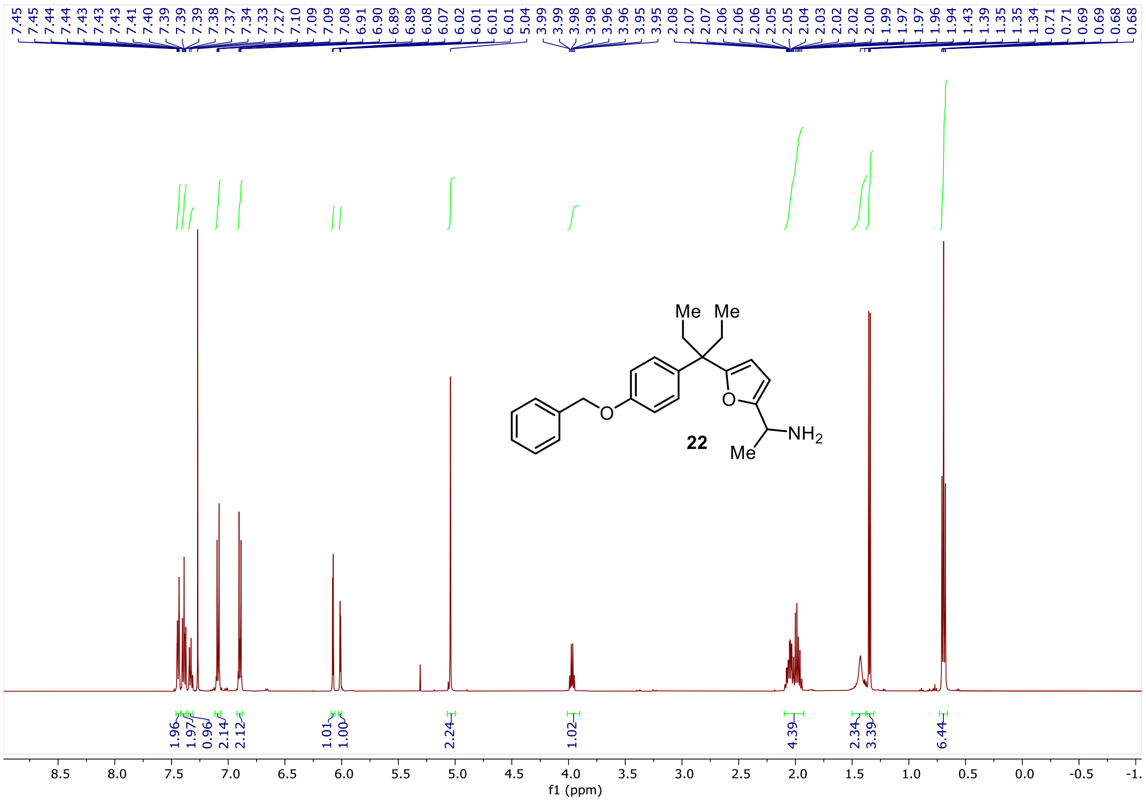

7.45
7.45
7.44
7.43
7.43
7.43
7.41
7.40
7.39
7.39
7.39
7.38
7.37
7.34
7.33
7.27
7.10
7.09
7.09
7.08
6.91
6.90
6.89
6.89
6.08
6.07
6.02
6.01
6.01
6.01
5.04
3.99
3.99
3.98
3.98
3.96
3.96
3.95
2.08
2.07
2.06
2.06
2.05
2.04
2.04
2.03
2.02
2.02
2.00
1.99
1.97
1.97
1.96
1.94
1.43
1.39
1.35
1.35
1.34
0.71
0.71
0.69
0.69
0.68
0.68
Me
Me
O
O
NH2
Me
22
1.96
1.97
0.96
2.14
2.12
1.01
1.00
2.24
1.02
4.39
2.34
3.39
6.44
f1 (ppm)

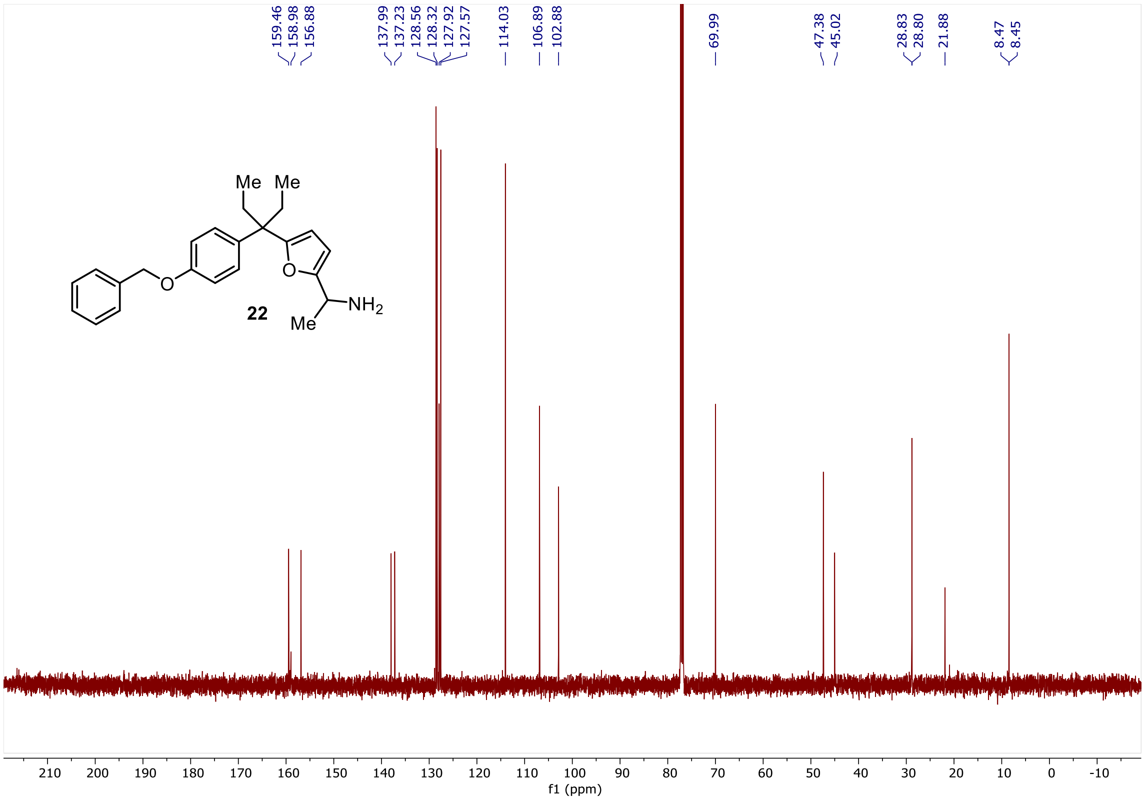

159.46
158.98
156.88
137.99
137.23
128.56
128.32
127.92
127.57
114.03
106.89
102.88
69.99
47.38
45.02
28.83
28.80
21.88
8.47
8.45
Me
Me
O
NH2
22
Me
210
200
190
180
170
160
150
140
130
120
110
100
90
80
70
60
50
40
30
20
10
0
-10
f1 (ppm)

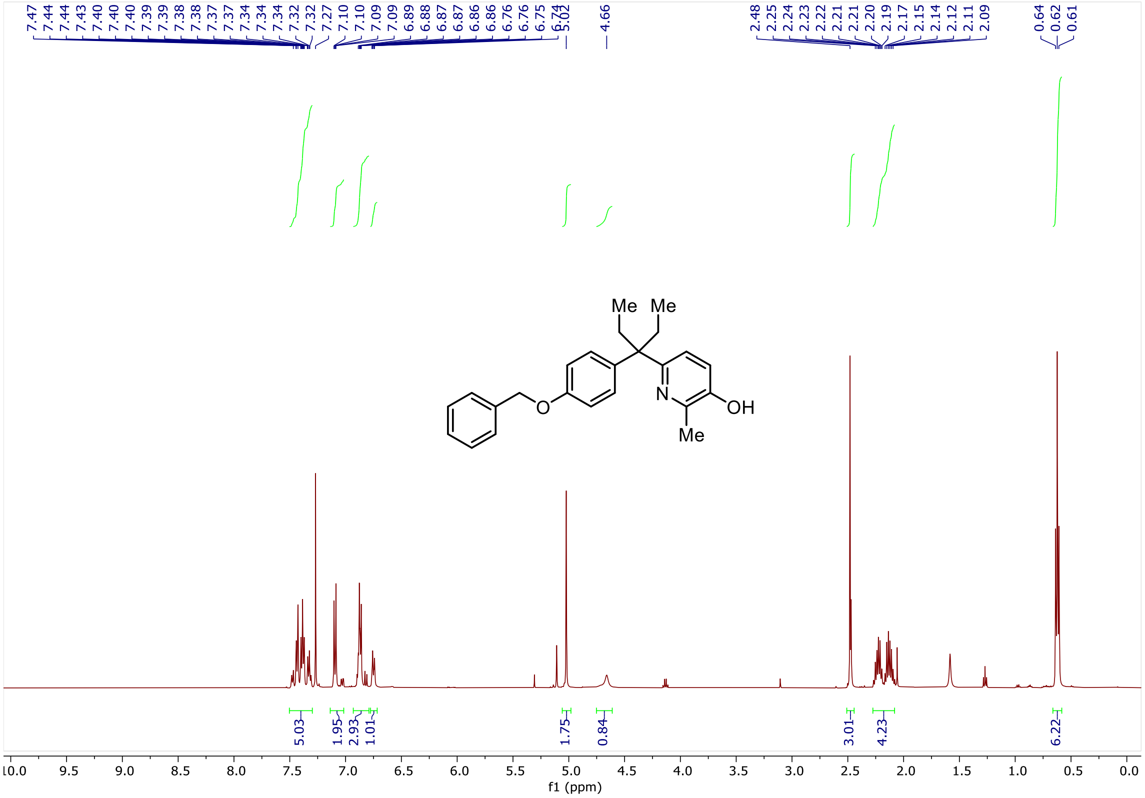

7.47
7.44
7.43
7.40
7.40
7.40
7.39
7.39
7.38
7.37
7.37
7.34
7.34
7.34
7.32
7.32
7.27
7.10
7.10
7.09
7.09
6.89
6.88
6.87
6.87
6.86
6.86
6.76
6.76
6.75
6.74
5.02
4.66
2.48
2.25
2.24
2.23
2.22
2.21
2.21
2.20
2.19
2.17
2.15
2.14
2.12
2.11
2.09
0.64
0.62
0.61
Me
Me
O
N
OH
Me
5.03
1.95
2.93
1.01
1.75
0.84
3.01
4.23
6.22
10.0
9.5
9.0
8.5
8.0
7.5
7.0
6.5
6.0
5.5
5.0
4.5
4.0
3.5
3.0
2.5
2.0
1.5
1.0
0.5
0.0
f1 (ppm)

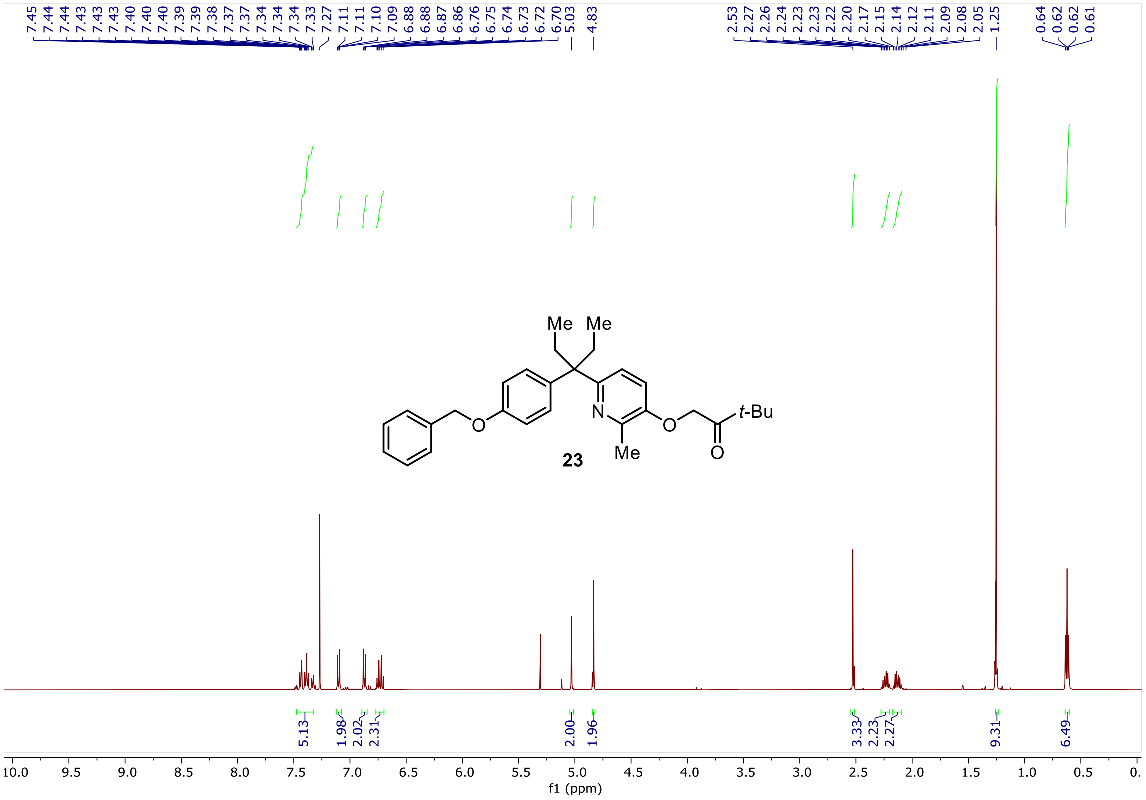
7.45 7.44 7.44 7.43 7.43 7.43 7.40 7.40 7.39 7.39 7.38 7.37 7.34 7.34 7.33 7.27 7.11 7.11 7.10 7.09 6.88 6.88 6.87 6.86 6.76 6.75 6.74 6.73 6.72 6.70 5.03 4.83
2.53 2.27 2.26 2.24 2.23 2.23 2.22 2.20 2.17 2.15 2.14 2.12 2.11 2.09 2.08 2.05 1.25
0.64 0.62 0.62 0.61
Me Me
N
O
O
t-Bu
O
Me
23
5.13 1.98 2.02 2.31 2.00 1.96 3.33 2.23 2.27 9.31 6.49
10.0 9.5 9.0 8.5 8.0 7.5 7.0 6.5 6.0 5.5 5.0 4.5 4.0 3.5 3.0 2.5 2.0 1.5 1.0 0.5 0.
f1 (ppm)

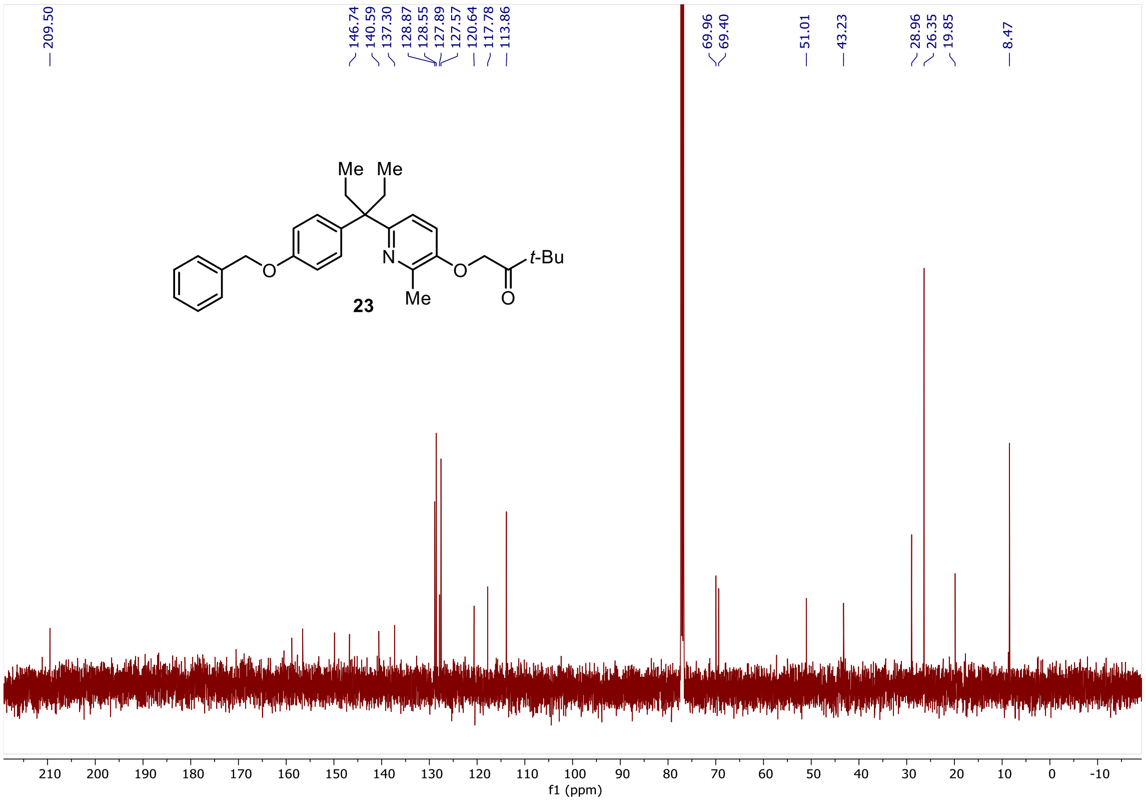
209.50
146.74 140.59 137.30 128.87 128.55 127.89 127.57 120.64 117.78 113.86
69.96 69.40
51.01
43.23
28.96 26.35 19.85
8.47
Me Me
N
O
O
t-Bu
O
Me
23
210 200 190 180 170 160 150 140 130 120 110 100 90 80 70 60 50 40 30 20 10 0 -10
f1 (ppm)

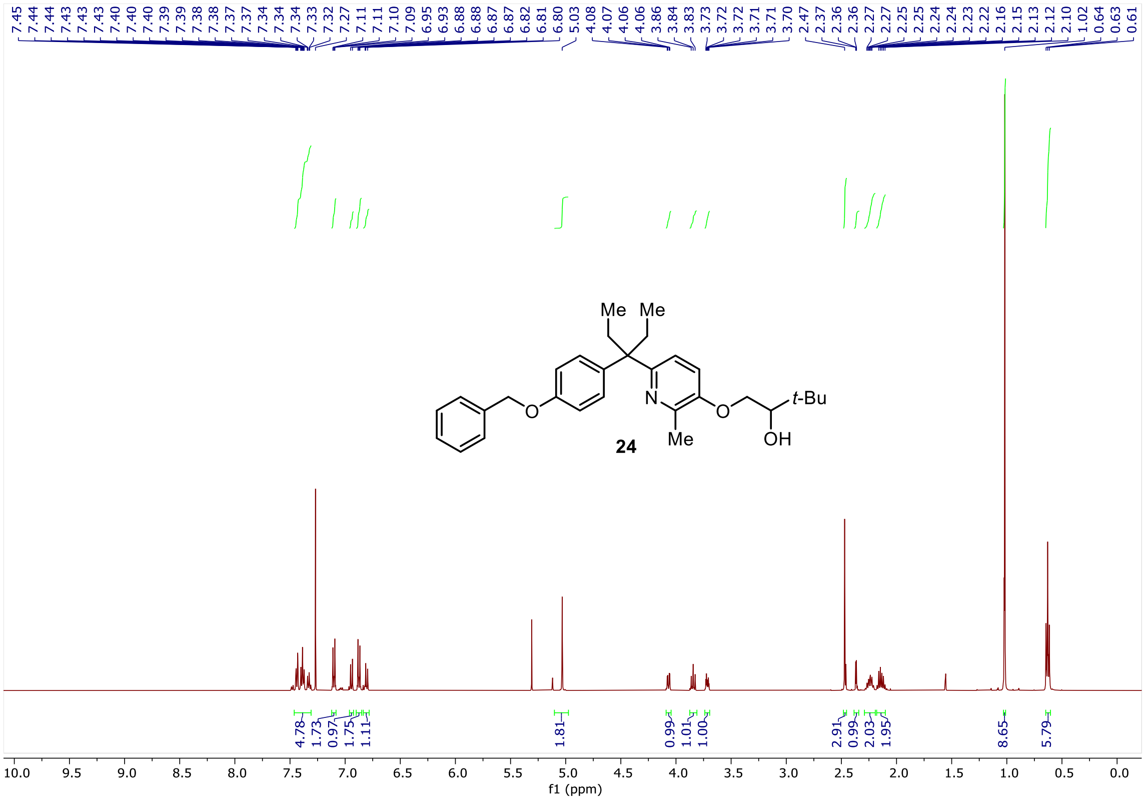
7.45 7.44 7.44 7.43 7.43 7.43 7.40 7.40 7.39 7.39 7.38 7.38 7.37 7.37 7.34 7.34 7.34 7.33 7.32 7.27 7.11 7.11 7.10 7.09 6.95 6.93 6.88 6.88 6.87 6.87 6.82 6.81 6.80 5.03 4.08 4.07 4.06 4.06 3.86 3.84 3.83 3.73 3.72 3.72 3.71 3.71 3.70 2.47 2.37 2.36 2.36 2.27 2.27 2.25 2.25 2.24 2.24 2.23 2.22 2.16 2.15 2.13 2.12 2.10 1.02 0.64 0.63 0.61
Me Me
N
O
O
t-Bu
Me
OH
24
4.78 1.73 0.97 1.75 1.11 1.81 0.99 1.01 1.00 2.91 0.99 2.03 1.95 8.65 5.79
10.0 9.5 9.0 8.5 8.0 7.5 7.0 6.5 6.0 5.5 5.0 4.5 4.0 3.5 3.0 2.5 2.0 1.5 1.0 0.5 0.0
f1 (ppm)

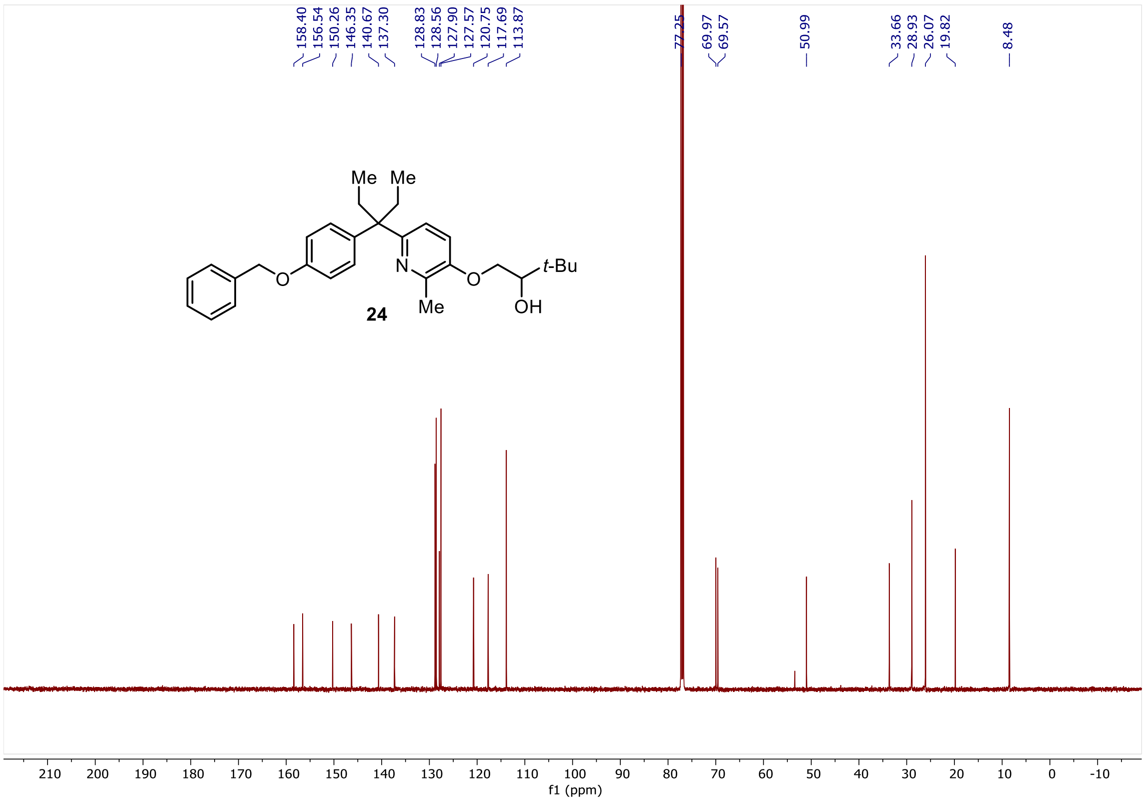
158.40 156.54 150.26 146.35 140.67 137.30 128.83 128.56 127.90 127.57 120.75 117.69 113.87 77.25 69.97 69.57 50.99 33.66 28.93 26.07 19.82 8.48
Me Me
N
O
O
t-Bu
Me
OH
24
210 200 190 180 170 160 150 140 130 120 110 100 90 80 70 60 50 40 30 20 10 0 -10
f1 (ppm)

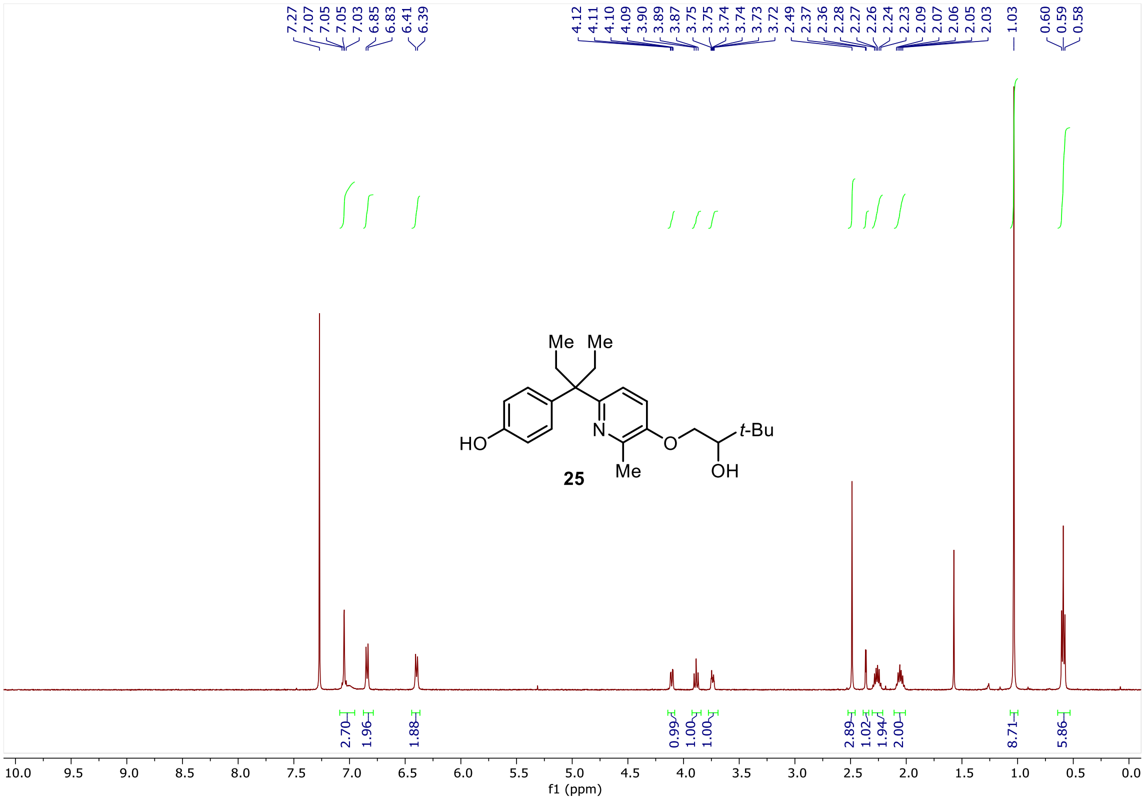
7.27
7.07
7.05
7.05
7.03
6.85
6.83
6.41
6.39
4.12
4.11
4.10
4.09
3.90
3.89
3.87
3.75
3.75
3.74
3.74
3.73
3.72
2.49
2.37
2.36
2.28
2.27
2.26
2.24
2.23
2.09
2.07
2.06
2.05
2.03
1.03
0.60
0.59
0.58
Me
Me
HO
N
O
t-Bu
OH
Me
25
2.70
1.96
1.88
0.99
1.00
1.00
2.89
1.02
1.94
2.00
8.71
5.86
10.0
9.5
9.0
8.5
8.0
7.5
7.0
6.5
6.0
5.5
5.0
4.5
4.0
3.5
3.0
2.5
2.0
1.5
1.0
0.5
0.0
f1 (ppm)

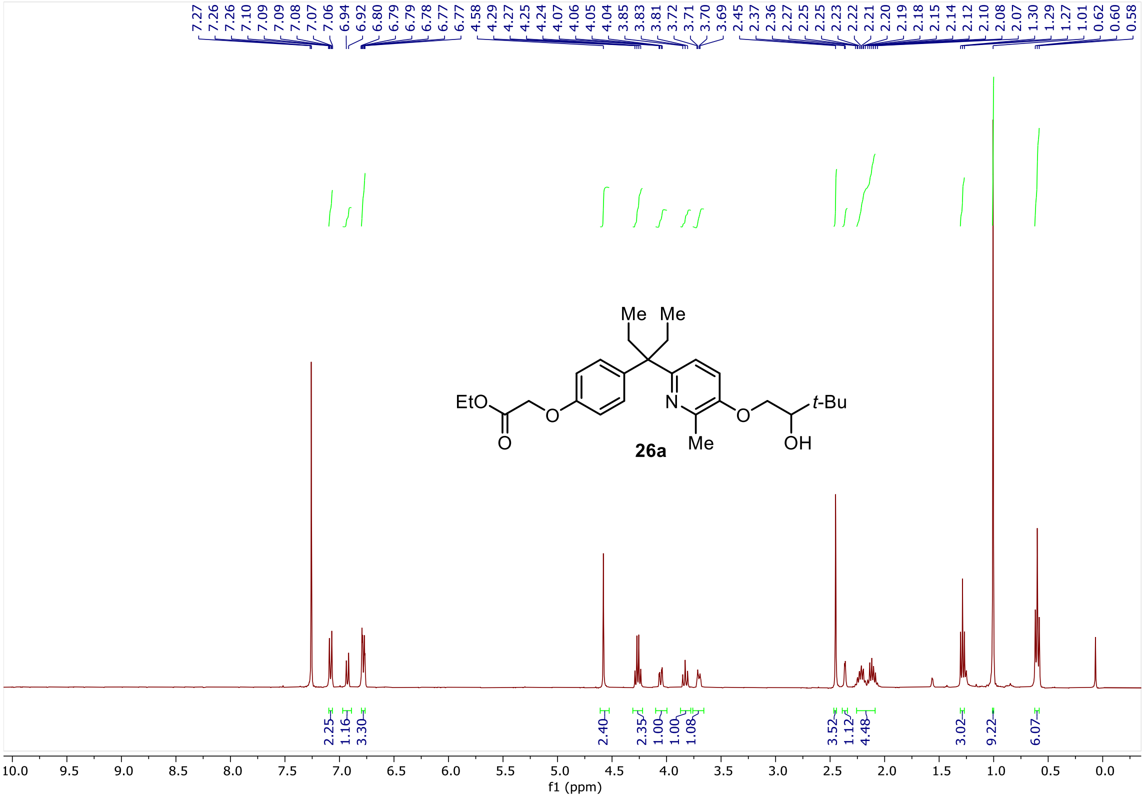
7.27
7.26
7.26
7.10
7.09
7.09
7.08
7.07
7.06
6.94
6.92
6.80
6.79
6.79
6.78
6.77
6.77
4.58
4.29
4.27
4.25
4.24
4.07
4.06
4.05
4.04
3.85
3.83
3.81
3.72
3.71
3.70
3.69
2.45
2.37
2.36
2.27
2.25
2.25
2.23
2.22
2.21
2.20
2.19
2.18
2.15
2.14
2.12
2.10
2.08
2.07
1.30
1.29
1.27
1.01
0.62
0.60
0.58
Me
Me
EtO
O
O
N
O
t-Bu
OH
Me
26a
2.25
1.16
3.30
2.40
2.35
1.00
1.00
1.08
3.52
1.12
4.48
3.02
9.22
6.07
10.0
9.5
9.0
8.5
8.0
7.5
7.0
6.5
6.0
5.5
5.0
4.5
4.0
3.5
3.0
2.5
2.0
1.5
1.0
0.5
0.0
f1 (ppm)

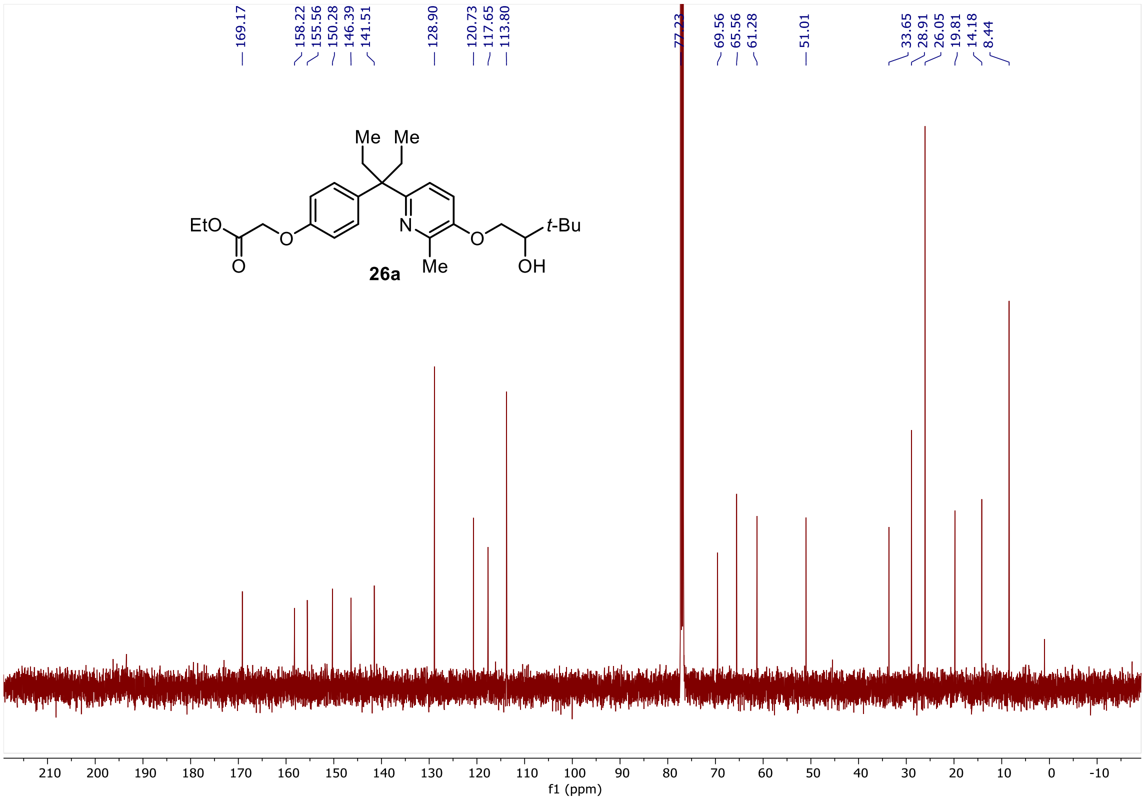

169.17
158.22
155.56
150.28
146.39
141.51
128.90
120.73
117.65
113.80
77.23
69.56
65.56
61.28
51.01
33.65
28.91
26.05
19.81
14.18
8.44
Me Me
EtO
O
N
O
t-Bu
O
OH
Me
26a
210 200 190 180 170 160 150 140 130 120 110 100 90 80 70 60 50 40 30 20 10 0 -10
f1 (ppm)

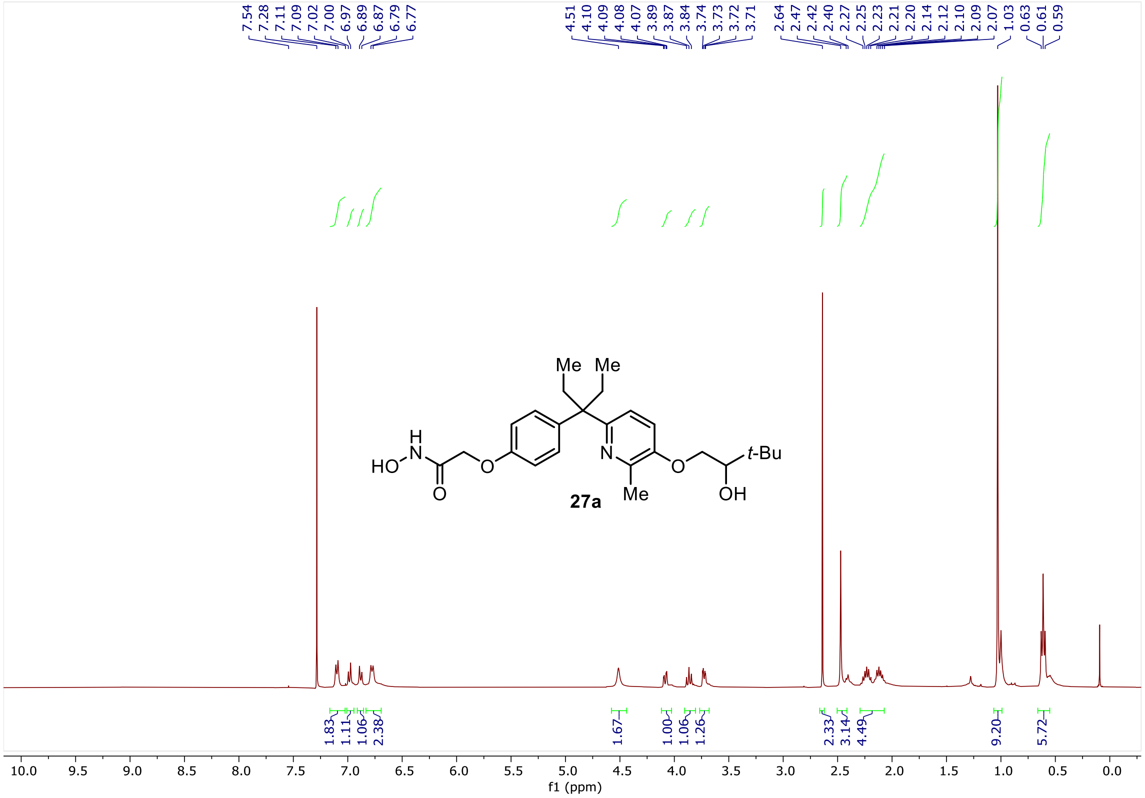

7.54
7.28
7.11
7.09
7.02
7.00
6.97
6.89
6.87
6.79
6.77
4.51
4.10
4.09
4.08
4.07
3.89
3.87
3.84
3.74
3.73
3.72
3.71
2.64
2.47
2.42
2.40
2.27
2.25
2.23
2.21
2.20
2.14
2.12
2.10
2.09
2.07
1.03
0.63
0.61
0.59
Me Me
H
HO N
O
O
N
O
t-Bu
27a
Me
OH
1.83
1.11
1.06
2.38
1.67
1.00
1.06
1.26
2.33
3.14
4.49
9.20
5.72
10.0 9.5 9.0 8.5 8.0 7.5 7.0 6.5 6.0 5.5 5.0 4.5 4.0 3.5 3.0 2.5 2.0 1.5 1.0 0.5 0.0
f1 (ppm)

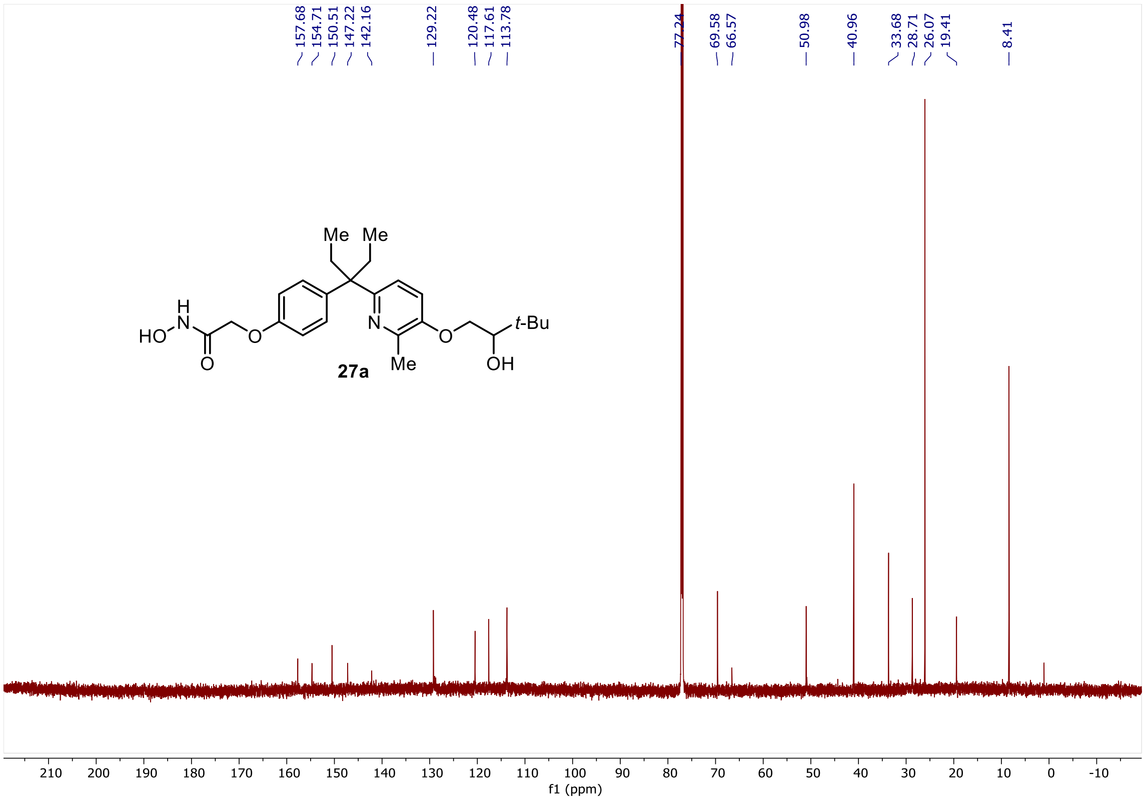

157.68
154.71
150.51
147.22
142.16
129.22
120.48
117.61
113.78
77.24
69.58
66.57
50.98
40.96
33.68
28.71
26.07
19.41
8.41
Me Me
HO
H
N
O
O
N
O
t-Bu
OH
Me
27a
f1 (ppm)

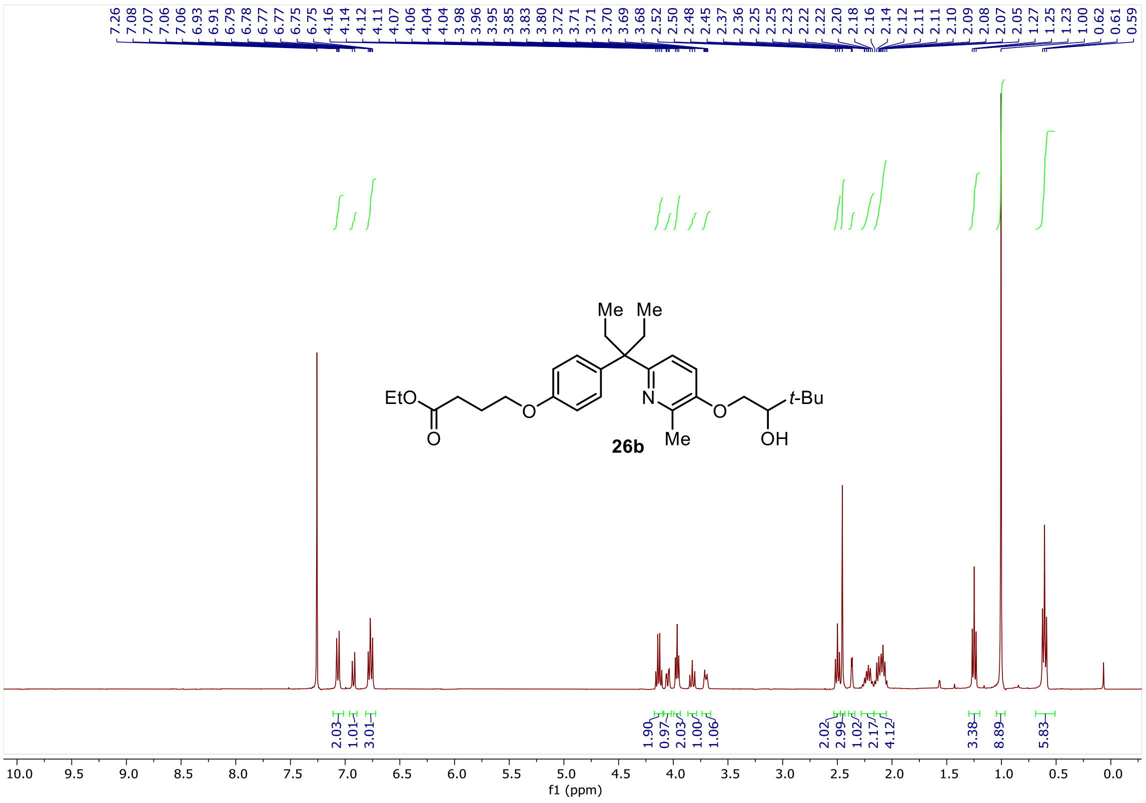

EtO
O
O
Me Me
N
O
t-Bu
OH
Me
26b
2.03
1.01
3.01
1.90
0.97
2.03
1.00
1.06
2.02
2.99
1.02
2.17
4.12
3.38
8.89
5.83
f1 (ppm)

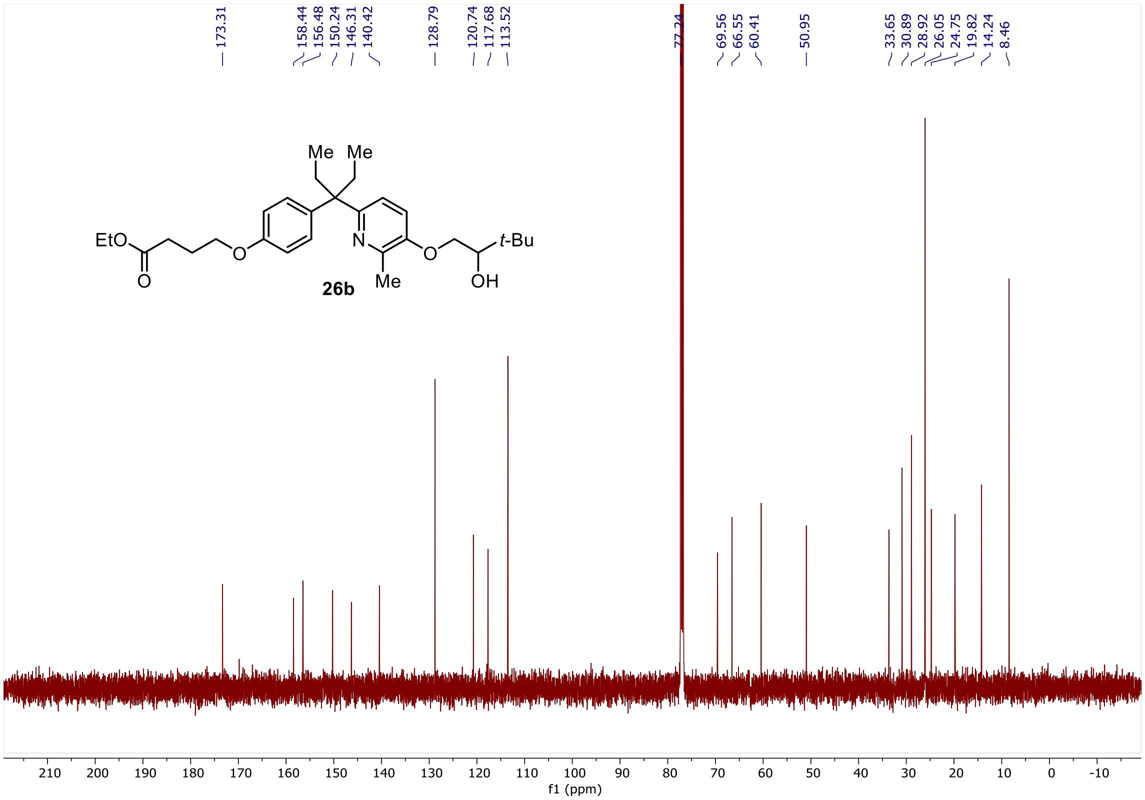
173.31
158.44
156.48
150.24
146.31
140.42
128.79
120.74
117.68
113.52
77.24
69.56
66.55
60.41
50.95
33.65
30.89
28.92
26.05
24.75
19.82
14.24
8.46
Me Me
EtO
O
O
N
O
t-Bu
OH
Me
26b
210 200 190 180 170 160 150 140 130 120 110 100 90 80 70 60 50 40 30 20 10 0 -10
f1 (ppm)

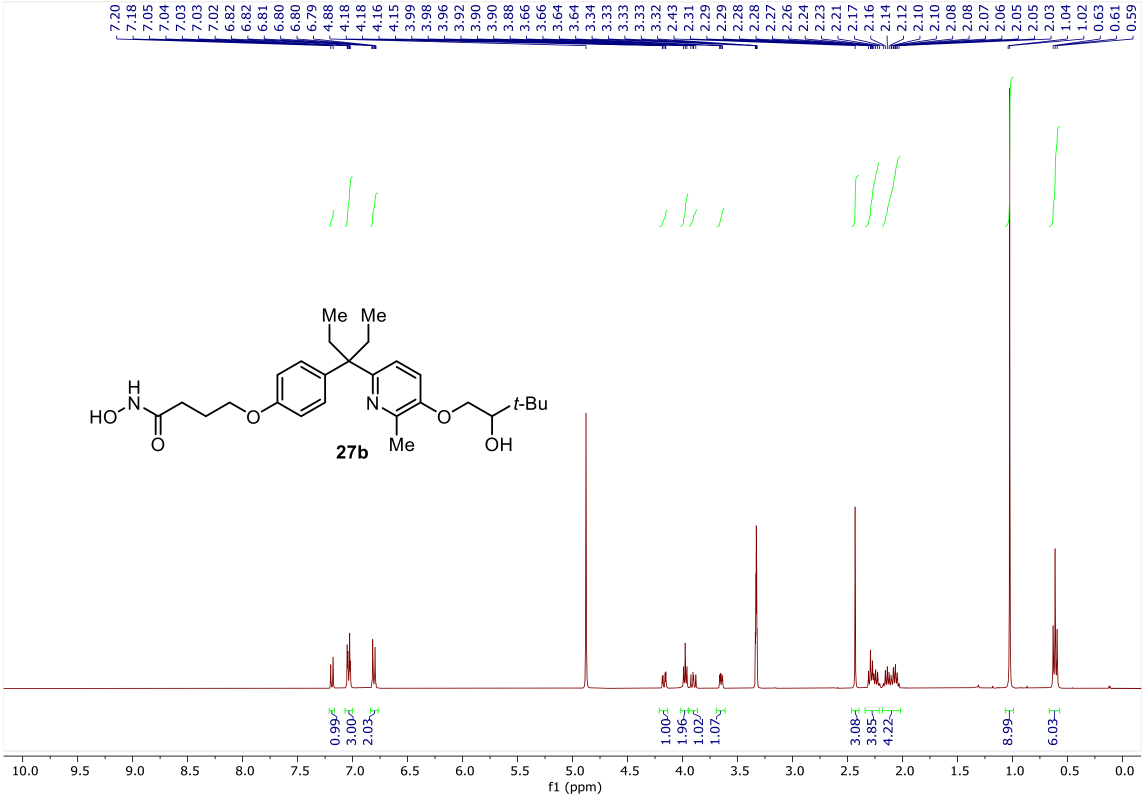
7.20
7.18
7.05
7.04
7.03
7.03
7.02
6.82
6.82
6.81
6.80
6.80
6.79
4.88
4.18
4.18
4.16
4.15
3.99
3.98
3.96
3.92
3.90
3.88
3.66
3.66
3.64
3.64
3.34
3.33
3.33
3.33
3.32
2.43
2.31
2.29
2.29
2.28
2.27
2.26
2.24
2.23
2.21
2.17
2.16
2.14
2.12
2.10
2.08
2.07
2.06
2.05
2.03
1.04
1.02
0.63
0.61
0.59
Me Me
H
N
HO
O
O
N
O
t-Bu
OH
Me
27b
0.99
3.00
2.03
1.00
1.96
1.02
1.07
3.08
3.85
4.22
8.99
6.03
10.0 9.5 9.0 8.5 8.0 7.5 7.0 6.5 6.0 5.5 5.0 4.5 4.0 3.5 3.0 2.5 2.0 1.5 1.0 0.5 0.0
f1 (ppm)

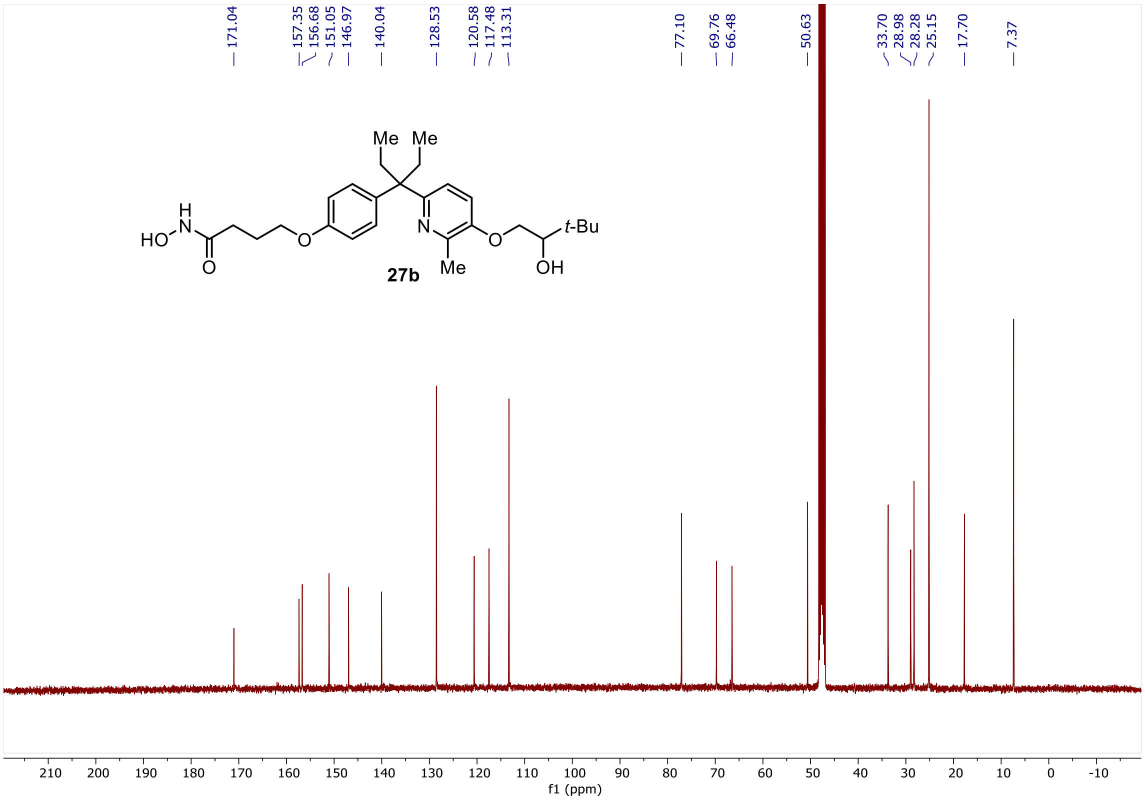
171.04
157.35
156.68
151.05
146.97
140.04
128.53
120.58
117.48
113.31
77.10
69.76
66.48
50.63
33.70
28.98
28.28
25.15
17.70
7.37
Me Me
HO
H N
O
O
N
Me
O
t-Bu
OH
27b
210 200 190 180 170 160 150 140 130 120 110 100 90 80 70 60 50 40 30 20 10 0 -10
f1 (ppm)

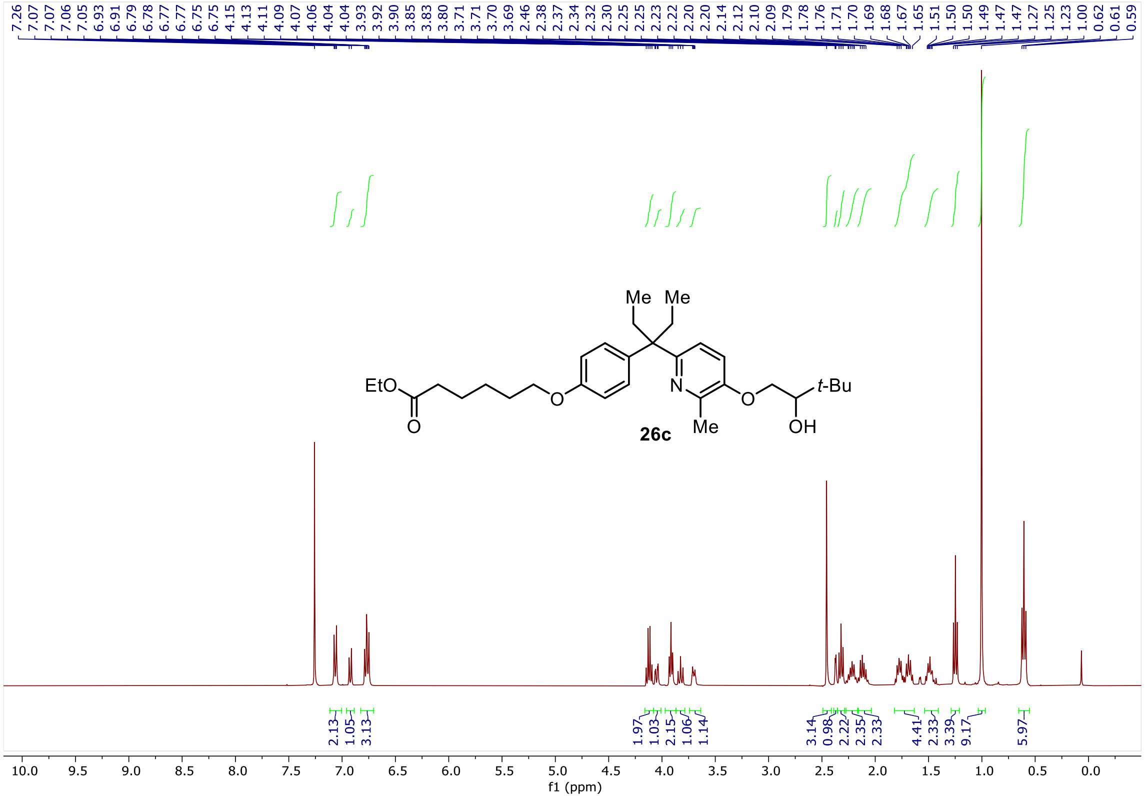
7.26 7.07 7.07 7.06 7.05 6.93 6.91 6.79 6.78 6.77 6.77 6.75 6.75 4.15 4.13 4.11 4.09 4.07 4.06 4.04 4.04 3.93 3.92 3.90 3.85 3.83 3.80 3.71 3.70 3.69 2.46 2.38 2.37 2.34 2.32 2.30 2.25 2.25 2.23 2.22 2.20 2.20 2.14 2.12 2.10 2.09 1.79 1.78 1.76 1.71 1.70 1.69 1.68 1.67 1.65 1.51 1.50 1.50 1.49 1.47 1.47 1.27 1.25 1.23 1.00 0.62 0.61 0.59
Me Me
EtO
O
O
N
Me
O
t-Bu
OH
26c
2.13 1.05 3.13 1.97 1.03 2.15 1.06 1.14 3.14 0.98 2.22 2.35 2.33 4.41 2.33 3.39 9.17 5.97
10.0 9.5 9.0 8.5 8.0 7.5 7.0 6.5 6.0 5.5 5.0 4.5 4.0 3.5 3.0 2.5 2.0 1.5 1.0 0.5 0.0
f1 (ppm)

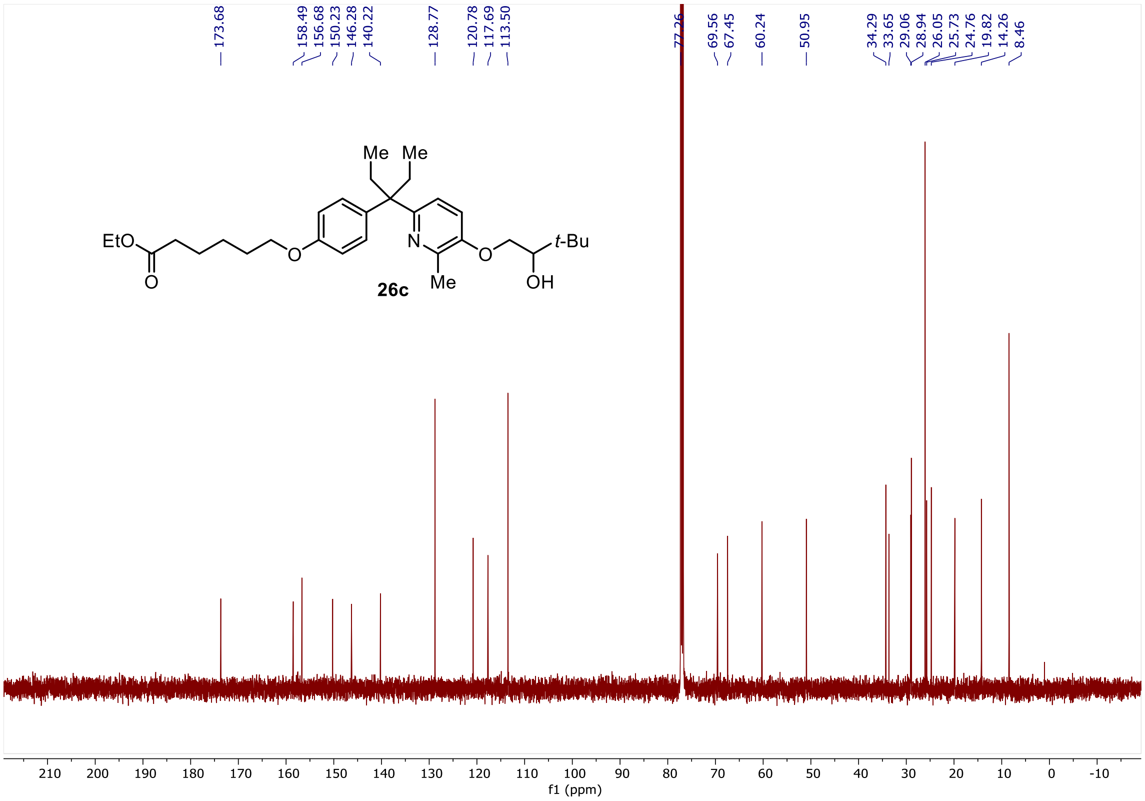
26c
f1 (ppm)

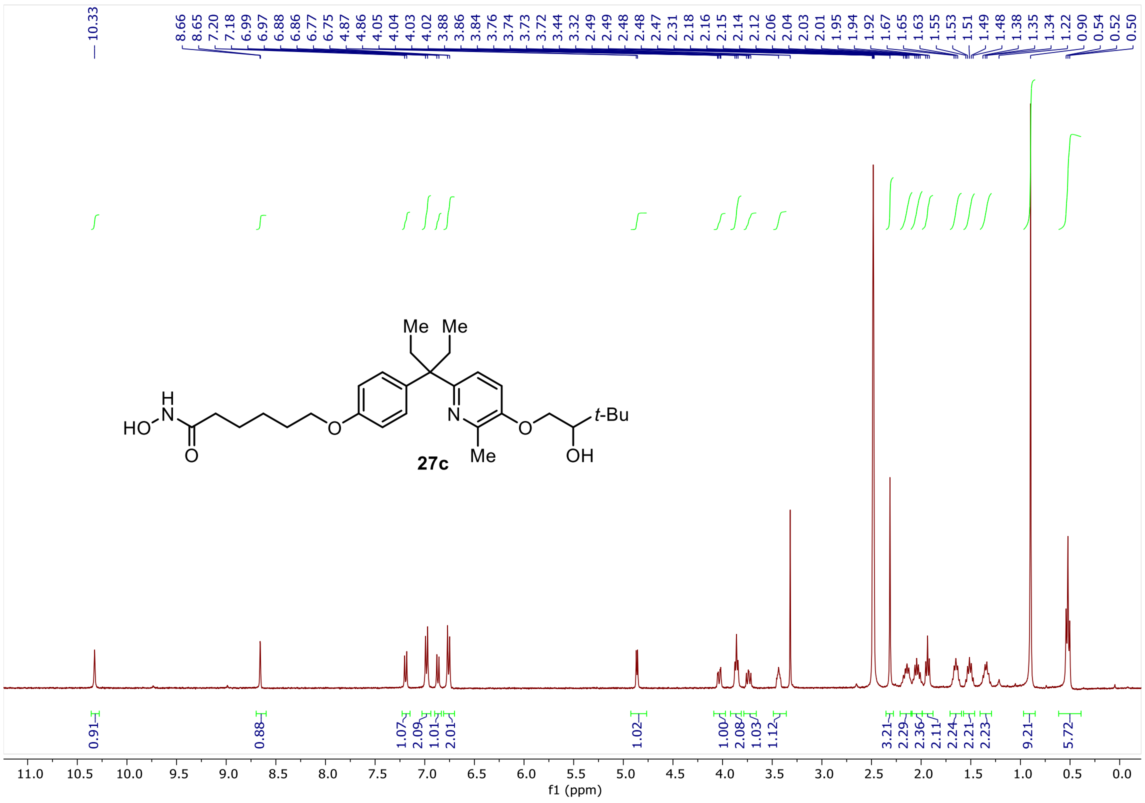
27c
f1 (ppm)

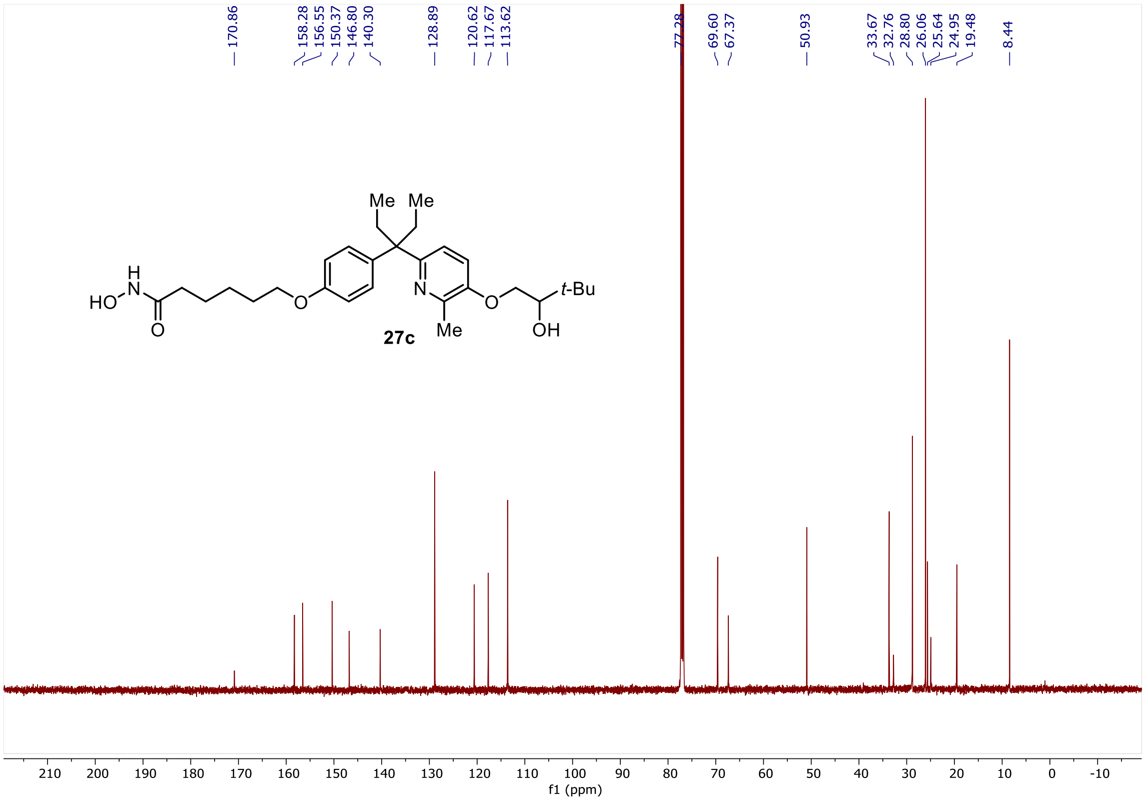

170.86
158.28
156.55
150.37
146.80
140.30
128.89
120.62
117.67
113.62
77.28
69.60
67.37
50.93
33.67
32.76
28.80
26.06
25.64
24.95
19.48
8.44
Me
Me
HO
H
N
O
O
N
O
t-Bu
Me
OH
27c
210 200 190 180 170 160 150 140 130 120 110 100 90 80 70 60 50 40 30 20 10 0 -10
f1 (ppm)
