# Supplementary material for: Molecular mechanisms of bifunctional vitamin D receptor agonist-histone deacetylase inhibitor hybrid molecules in triple-negative breast cancer
Source: Sci Rep. 2022 Apr 25;12:6745. doi: 10.1038/s41598-022-10740-9 (PMC9038752; doi:10.1038/s41598-022-10740-9)
Supplement: Supplementary file 4 — Supplementary Information 4. [file 41598_2022_10740_MOESM4_ESM.pdf]

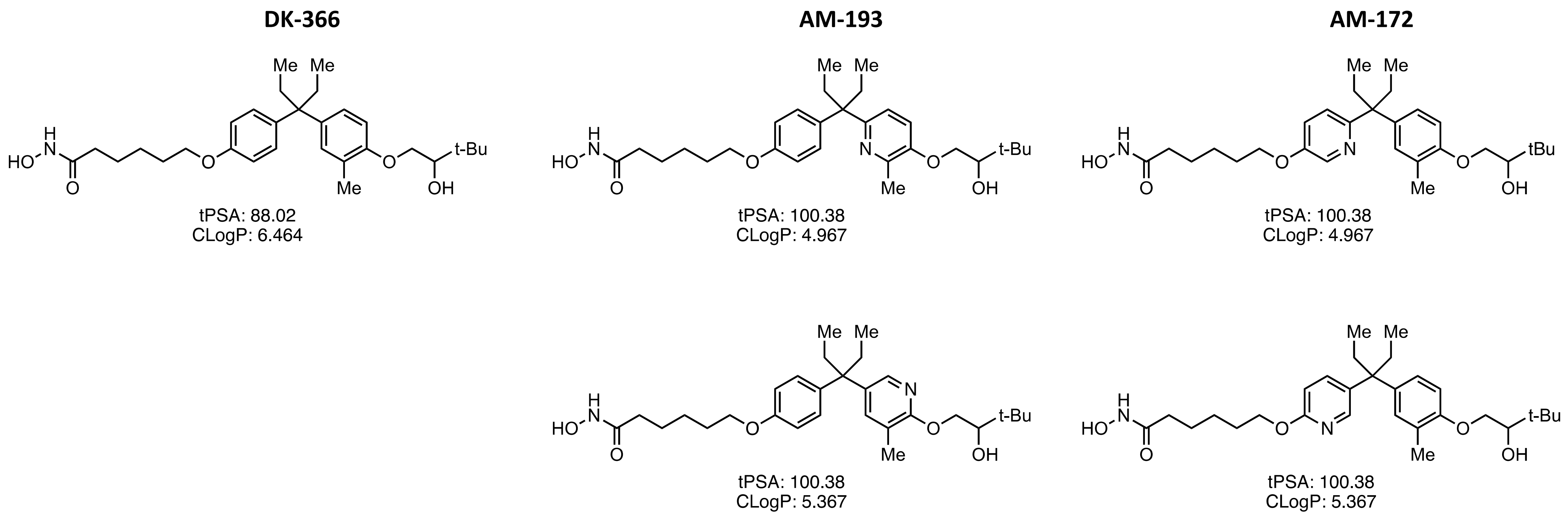

Fig. S1. Computed log*P* values of different heterocyclic VDR agonist/HDACi hybrids compared to related carbocyclic hybrid DK-366. CLog*P* values were computed using ChemDraw. Dk-366 and heterocycles AM-193 and AM-172, tested in this study, are indicated.

Western-Blot Fig 2.b

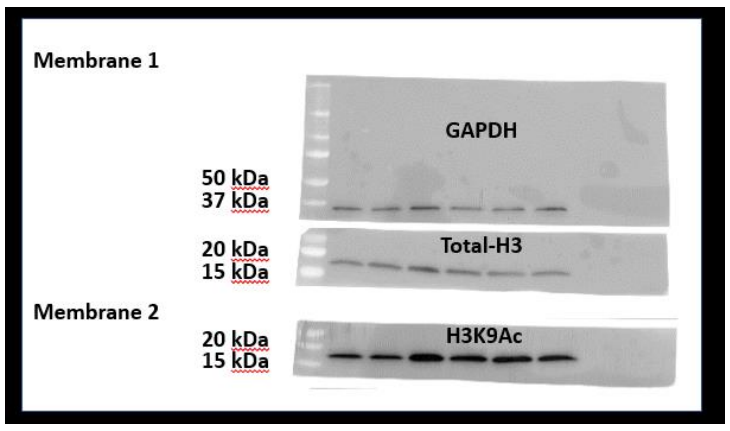

Western-Blot Fig 2.c

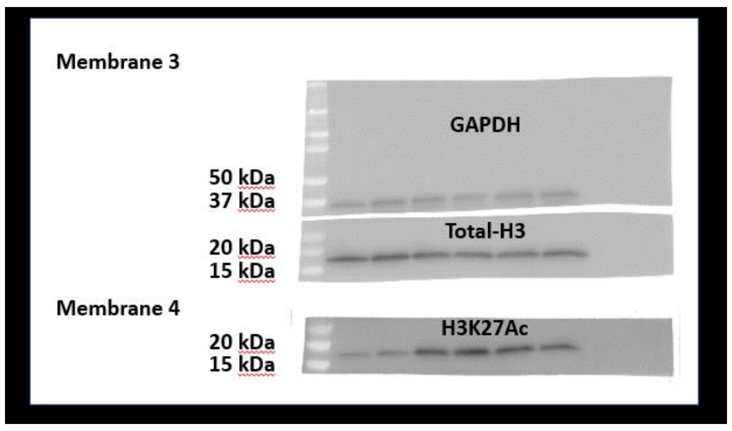

Western-Blot Fig 2.d

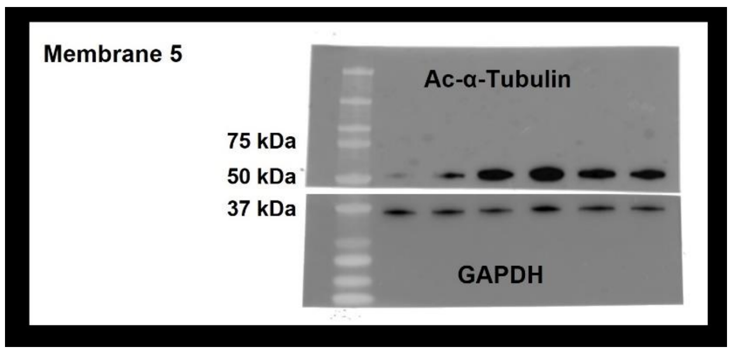

Western-Blot Fig 2.d

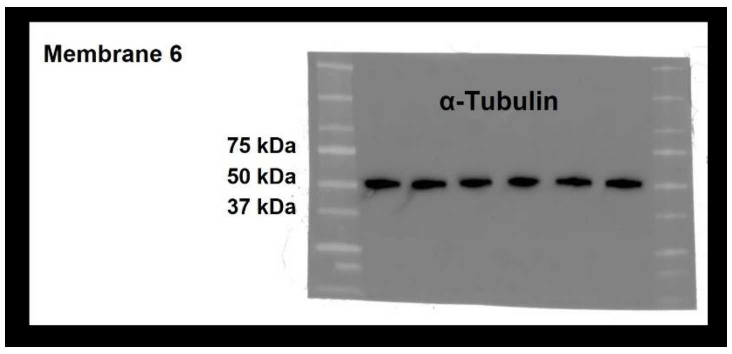

Fig. S2. Full-sized versions of western blots shown in Fig. 2. Membranes **1** and **3** were cut between 25 kDa and 37 kDa before hybridization. Membrane 5 was cut between 37 kDa and 50 kDa prior to hybridization.

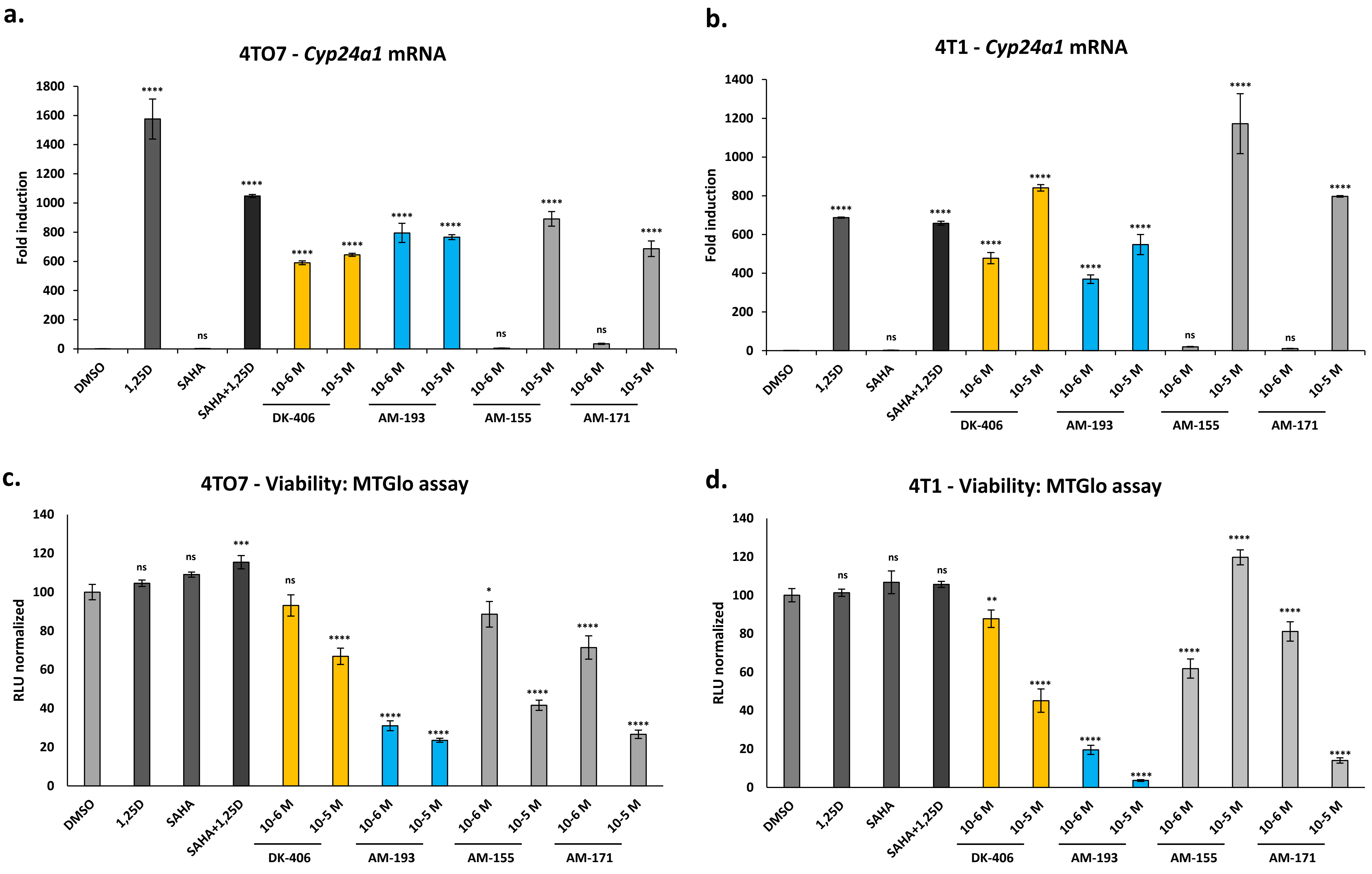

Fig. S3. a. b. Comparison of induction of expression of the VDR target gene *Cyp24a1* in 4TO7 (**a**) and 4T1 (**b**) cells by 1,25D and novel hybrid molecules. **c. d.** Assessment of cytotoxicity by MTGlo assay of 1,25D, and hybrids DK-406, AM-193, AM-155 and AM-171 in 4TO7 (**c**) and 4T1 (**d**) cells.

a.

b.

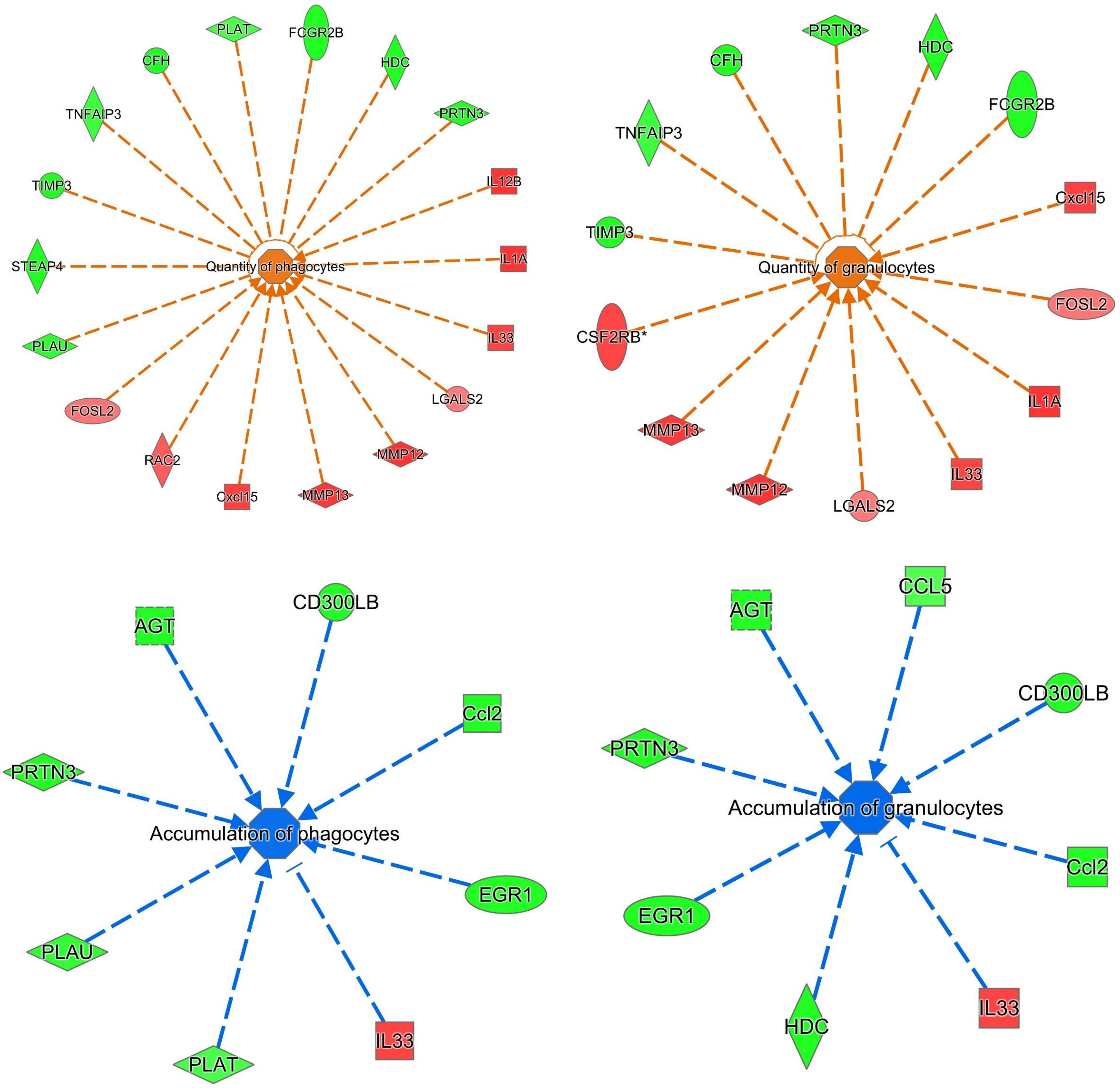

Fig S4. Selected diseases and biological functions that are predicted to be activated (**a.**) and inhibited (**b.**) following 24h treatment with AM-193. Functions are displayed as nodes (genes) and edges (biological relationship between nodes). Colour intensity of each node represents log fold change expression; red and green indicate upregulated and downregulated genes, respectively. Edges connecting the genes to the respective functions indicate the predicted relationships. The colour of the central function and edges indicate activation (orange) or inhibition (blue) of the different functions.

Table S1 and Table S2 : See Excel files with RNAseq results and analysis

Supplemental Table 1.

RNAseq results and statistical analyses from D, S, S+D, and DK-406 conditions measured relative to control. 4TO7 cells were either treated with 1,25D (D), SAHA (S), SAHA in the presence of 1,25D (S+D), or DK-406 for 6 or 48 hours prior to RNA extraction. Samples were prepared in triplicate and run on Illumina NovaSeq 6000 S4 PE100. For assessing differential gene expression for RNAseq data, the LIMMA package in R was used.

Supplemental Table 2.

RNAseq results and statistical analyses from D, S, S+D, DK-406, and AM-193 conditions measured relative to control. 4TO7 cells were treated with either 1,25D (D), SAHA (S), SAHA in the presence of 1,25D (S+D), DK-406, or AM-193 for 6 or 24 hours prior to RNA extraction. Samples were prepared in triplicate and run on Illumina NovaSeq 6000 S4 PE100. For assessing differential gene expression for RNAseq data, the LIMMA package in R was used.

Primers RT-qPCR

Targeted gene	Sequence forward	Sequence reverse
Cyp24a1	GAAGCTGTGAACTTCATCAT	CTGCACTAGGCTGCTGAGAA
Il1α	TCAAGCAACGGGAAGATTCTGA	CTCTGGTAGGTGTAAGGTGCTG
Il12β	CACGGCAGCAGAATAAATATGA	GACCTCCACCTGTGAGTTCTTC
Mmp12	GTGAAGAAGGTTGATGCAGCTG	TGTGGAAATCAGCTTGGGGTAA
Mmp13	AAAGAGGTGAAGAGACTGAGCG	TGAGGCGGGGATAATCTTTGTC
Fosl2	AGGGAGAGAAACAAGCTAGCTG	GCCACCAACATGAACTCTAGCT
Ccl20	ACATACAGACGCCTCTTCCTTC	GCGCACACAGATTTTCTTTTCT
Ccl5	AGCAGCAAGTGCTCCAATCT	CTTGAACCCACTTCTTCTCTGG
Cxcl10	GCCATAGGGAAGCTTGAAATC	TCAGACATCTCTGCTCATCATTC
Cxcl11	AGCTGCTCAAGGCTTCCTTA	GGCACCTTTGTCGTTTATGAG
Ccl2	AGGTCCCTGTCATGCTTCTG	TCTCTTGAGCTTGGTGACAAAA
18S	GCAATTATTCCCCATGAACG	GGGACTTAATCAACGCAAGC

Supplemental Table S3. Primer sequences for RT-qPCR.
